# Supplementary material for: Synthesis of 3-(2-Alkylthio-4-chloro-5-methylbenzenesulfonyl)-2-(1-phenyl-3-arylprop-2-enylideneamino)guanidine Derivatives with Pro-Apoptotic Activity against Cancer Cells
Source: Int J Mol Sci. 2023 Feb 23;24(5):4436. doi: 10.3390/ijms24054436 (PMC10002375; doi:10.3390/ijms24054436)
Supplement: Supplementary file 1 [file ijms-24-04436-s001.zip › ijms-2146604-supplementary.pdf]

Supplementary material

Synthesis of 3-(2-Alkylthio-4-chloro-5-methylbenzenesulfonyl)-2-(1-phenyl-3-arylprop-2-enylideneamino)guanidine Derivatives with Pro-Apoptotic Activity against Cancer Cells

Aneta Pogorzelska [1,*], Jarosław Sławiński [1,*], Anna Kawiak [2], Grzegorz Stasiłojć [3] and Jarosław Chojnacki [4]

[1] Department of Organic Chemistry, Medical University of Gdańsk, Al. Gen. J. Hallera 107, 80-416 Gdańsk, Poland
[2] Department of Biotechnology, Intercollegiate Faculty of Biotechnology, University of Gdańsk and Medical University of Gdańsk, Abrahama 58, 80-307 Gdańsk, Poland
[3] Department of Cell Biology and Immunology, Intercollegiate Faculty of Biotechnology of UG and MUG, Medical University of Gdańsk, Dębinki 1, 80-211 Gdańsk, Poland
[4] Department of Inorganic Chemistry, Gdańsk University of Technology, Narutowicza 11/12, 80-233 Gdańsk, Poland
* Correspondence: aneta.pogorzelska@gumed.edu.pl (A.P.); jaroslaw.slawinski@gumed.edu.pl (J.S.)

Table of contents

S.1. Materials and methods **1**

S.1.1. General information **1**

S.1.2. Synthesis **2**

Spectra 1-52. ^{1}H NMR and ^{13}C NMR spectra of compds **8-33** **10-61**

S.2. Experimental details of crystallographic analysis **62**

S.3. Hydrogen-bond geometry (Å, º) for 24 and 31 **63**

S.1. Materials and methods

S.1.1. General information

Melting points of the compounds were determined with a Boethius apparatus. Infrared (IR) spectra were obtained by using Thermo Mattson Satellite FTIR spectrophotometer. Varian Gemini 200 apparatus or Varian Unity 500 Plus apparatus were used to record ^{1}H nuclear magnetic resonance (NMR) spectra at 200 MHz or 500 MHz, respectively. The chemical shifts were expressed in parts per million (ppm). PerkinElmer 2400 Series II CHN Elemental Analyzer was used to obtain the elemental analyses and the results were in agreement with the theoretical values within ±0.4% range.

The LC-MS analyses of the synthetized compounds were performed on QTRAP 6500 mass spectrometer (SCIEX) and coupled in line with Eksigent microLC 200 liquid chromatography system (Eksigent). Five μL of the analytes' solutions (in acetonitrile) were loaded using PAL DLW autosampler (PAL system) onto the HALO C18 column (0.5 x 50 mm, 2,7 μm, 90A; from Eksigent) and separated with the use of mobile phase A (0.1% formic acid in water) and B (0.1% formic acid in acetonitrile) and the following gradient: 0-1 min: 98% A and 2%B; 1-8min 2-98%B; 8-8.5 min: 2%A and 98%B, 8.5-9min: 98-2%B; 9-10min: 98%A and 2%B. All solvents were LC-MS grade and purchased at Merck. The column was equilibrated with the gradient starting conditions (98%A and 2%) 5 min pre-

and 1 min post- analysis. The eluate from the column was ionized by electrospray ionization in the TurboV Ion Source (SCIEX) operated in positive ion mode at ISFV 5500 V; 30 psi of CUR, GS1 and GS2 gas flow; at TEM 300C, 110 V DP and 10 V EP potentials; and analyzed in Q1 scan mode at 200 Da/s, in the range of 100-700 Da, at unit resolution.

The starting aminoguanidines **1-7** were obtained by the methods described previously [17, 21-23].

S.1.2. Synthesis

*General procedure for the preparation of 3-(2-alkylthio-4-chloro-5-methylbenzenesulfonyl)-2-(1-phenyl-3-arylprop-2-enylideneamino)guanidine **8-33***

The appropriate *N*-amino-*N'*-(2-alkylthio-4-chloro-5-methylbenzenesulfonyl)guanidine (**1**-**7**) (0.5 mmol) and PTSA (0.05 mmol, 9.5 mg) were suspended in ethanol (3 mL) and the chalcone derivative (0.5 mmol) was added. The reaction mixture was stirred at reflux for 1−8 h and the obtained precipitate was filtered off, dried, and crystallized from the appropriate solvent to afford the desired pure product.

*3-(2-Benzylthio-4-chloro-5-methylbenzenesulfonyl)-2-(1,3-diphenylprop-2-enylideneamino)guanidine (**8**)*

Starting from *N*-amino-*N*'-(2-benzylthio-4-chloro-5-methylbenzenesulfonyl)guanidine **1** (0.192 g) and *trans*-chalcone (0.104 g) with stirring for 8 h, the title compound **8** was obtained after crystallization from iPrOH (0.127 g, 44%): m.p. 194–197 °C, IR (KBr): 3444, 3334, 3304 (NH), 1636 (NH), 1522, 1492 (C=N, C=C), 1340, 1128 (SO_2) cm^{-1}, ^1H NMR (200 MHz, DMSO-d_6): δ 2.28 (s, 3H, CH_3), 4.28 (s, 2H, CH_2), 6.38 (d, 1H, *J* = 16.4 Hz, CH=C), 7.11–7.20 (m, 4H, C=CH, arom.), 7.21–7.49 (m, 10H, H-3, arom.), 7.56–7.74 (m, 5H, H-6, NH, arom.), 7.75 (br. s, 1H, NH), 9.92 (s, 1H, NH) ppm. ^{13}C NMR (125 MHz, DMSO-d_6): δ 19.45, 36.79, 127.36, 27.63, 128.33, 128.37, 128.67, 128.92, 129.35, 129.40, 129.65, 130.04, 130.33, 130.39, 130.96, 132.42, 135.91, 136.06, 136.47, 136.99, 137.03, 139.85, 153.99, 154.50 ppm. ESI-MS *m/z* calcd for $C_{30}H_{27}ClN_4O_2S_2$ [M+H$^+$] 575.1 found 575.0. Anal. calcd. for $C_{30}H_{27}ClN_4O_2S_2$ (575.14); C, 62.65; H, 4.73; N, 9.74. Found: C, 62.96; H, 4.88; N, 10.01.

*3-(2-Benzylthio-4-chloro-5-methylbenzenesulfonyl)-2-[3-(2-hydroxyphenyl)-1-phenylprop-2-enylideneamino]guanidine (**9**)*

Starting from **1** (0.192 g) and 2-hydroxychalcone (0.112 g) with stirring for 1 h, the title compound **9** was obtained after crystallization from EtOH/toluene (0.109 g, 37%): m.p. 188–193 °C; IR (KBr): 3466, 3275, 3200 (OH, NH), 1628 (NH), 1568, 1549, 1494, 1460 (C=N, C=C), 1321, 1160 (SO_2) cm^{-1}; ^1H NMR (500 MHz, DMSO-d_6): δ 2.26 (s, 3H, CH_3), 4.27 (s, 2H, CH_2), 6.56 (d, *J* = 16.6 Hz,1H, CH=C), 6.79–6.83 (m, 2H, C=CH, arom.), 7.10–7.15 (m, 3H, arom.), 7.18–7.24 (m, 2H, arom.), 7.30 (t, 2H, arom.), 7.38–7.42 (m, 3H, arom.), 7.44 (s, 1H, H-3), 7.52–7.55 (m, 4H, H-6, 3arom.), 7.58 (br. s, 1H, NH), 7.70 (br. s, 1H, NH), 9.82 (s, 2H, OH, NH) ppm. ^{13}C NMR (125 MHz, DMSO-d_6): δ 19.46, 36.77, 116.41, 119.91, 122.89, 127.40, 127.66, 128.01, 128.30, 128.42, 128.92, 129.64, 129.97, 130.23, 130.38, 130.44, 131.21, 132.37, 133.10, 135.90, 136.48, 136.95, 139.89, 154.41, 155.11, 155.81 ppm. ESI-MS *m/z* calcd for $C_{30}H_{27}ClN_4O_3S_2$ [M+H$^+$] 591.1 found 590.9. Anal. calcd. for $C_{30}H_{27}ClN_4O_3S_2$ (591.14); C, 60.95; H, 4.60; N, 9.48. Found: C, 60.74; H, 4.36; N, 9.61

*3-(2-Benzylthio-4-chloro-5-methylbenzenesulfonyl)-2-[3-(4-hydroxyphenyl)-1-phenylprop-2-enylideneamino]guanidine (**10**)*

Starting from **1** (0.192 g) and 4-hydroxychalcone (0.112 g) with stirring for 1 h, the title compound **10** was obtained after crystallization from EtOH/toluene (0.199 g, 30%): m.p. 213–216 °C; IR (KBr): 3413, 3228 (OH, NH), 1651 (NH), 1608, 1545, 1513, 1146 (C=N, C=C), 1339, 1167 (SO_2) cm^{-1}; ^1H NMR (500 MHz, DMSO-d_6): δ 2.26 (s, 3H, CH_3), 4.27 (s, 2H, CH_2), 6.24 (d, *J* = 16.6 Hz, 1H, CH=C), 6.73 (d, *J* = 8.3 Hz, 2H, arom.), 6.94 (d, *J* = 16.6 Hz, 1H, C=CH), 7.09–7.10 (m, 2H, arom.), 7.27–7.31 (m, 4H,

arom.), 7.38–7.44 (m, 4H, H-3, arom.), 7.51–7.54 (m, 5H, NH, H-6, arom.), 7.70 (br. s, 1H, NH), 9.80 (s, 2H, OH, NH) ppm. ^{13}C NMR (125 MHz, DMSO-*d$_6$*): δ 19.46, 36.75, 116.24, 125.30, 127.14, 127.63, 128.27, 128.36, 128.91, 129.03, 129.64, 129.98, 130.21, 130.37, 131.25, 132.35, 135.90, 136.47, 136.94, 137.50, 139.88, 154.41, 154.68, 158.88 ppm. ESI-MS *m/z* calcd for $C_{30}H_{27}ClN_4O_3S_2$ [M+H$^+$] 591.1 found 591.0. Anal. calcd. for $C_{30}H_{27}ClN_4O_3S_2$ (591.14); C, 60.95; H, 4.60; N, 9.48. Found: C, 60.85; H, 4.57; N, 9.57.

*3-(2-Benzylthio-4-chloro-5-methylbenzenesulfonyl)-2-[3-(4-methoxyphenyl)-1-phenylprop-2-enylideneamino]guanidine (**11**)*

Starting from **1** (0.192 g) and 4-methoxychalcone (0.119 g) with stirring for 6 h, the title compound **11** was obtained after crystallization from EtOH/*p*-dioxane 2:1 (0.197 g, 65%): m.p. 178−182 °C; IR (KBr): 3448, 3254 (NH), 1630 (NH), 1605, 1545, 1510 (C=N, C=C), 1336, 1173 (SO_2) cm^{-1}; ^{1}H NMR (200 MHz, DMSO-*d$_6$*): δ 2.29 (s, 3H, CH_3), 3.78 (s, 3H, OCH_3), 4.30 (s, 2H, CH_2), 6.32 (d, *J* = 16.4 Hz, 1H, CH=C), 6.93–7.09 (m, 2H, C=CH, arom.), 7.13–7.21 (m, 3H, arom.), 7.24–7.47 (m, 8H, H-3, arom.), 7.56 (m, 5H, H-6, NH, arom.), 7.74 (br. s, 1H, NH), 9.87 (s, 1H, NH) ppm; ^{13}C NMR (50 MHz, DMSO-*d$_6$*): δ 19.28, 36.64, 55.5, 114.68, 126.21, 127.46, 128.21, 128.57, 128.74, 129.47, 129.80, 129.91, 130.05, 130.23, 131.04, 132.21, 135.71, 136.33, 136.68, 136.75, 139.81, 154.24, 154.34, 160.15 ppm. ESI-MS *m/z* calcd for $C_{31}H_{29}ClN_4O_3S_2$ [M+H$^+$] 605.1 found 605.0. Anal. calcd. for $C_{31}H_{29}ClN_4O_3S_2$ (605.17); C, 61.53; H, 4.83; N, 9.26. Found: C, 61.19; H, 4.5.; N, 8.96.

*3-(2-Benzylthio-4-chloro-5-methylbenzenesulfonyl)-2-[3-(4-chlorophenyl)-1-phenylprop-2-enylideneamino]guanidine (**12**)*

Starting from **1** (0.192 g) and 4-chlorochalcone (0.121 g) with stirring for 1 h, the title compound **12** was obtained after crystallization from EtOH (0.229 g, 75%): m.p. 157−162 °C; IR (KBr): 3462, 3441, 3353, 3335, 3297 (NH), 1609 (NH), 1526, 1492, 1449 (C=N, C=C), 1329, 1170 (SO_2) cm^{-1}, ^{1}H NMR (500 MHz, DMSO-*d$_6$*): δ 2.26 (s, 3H, CH_3), 4.27 (s, 2H, CH_2), 6.36 (d, *J* = 16.6 Hz, 1H, CH=C), 7.11–7.14 (m, 3H, C=CH, arom.), 7.17–7.19 (m, 2H, arom.), 7.28–7.31 (t, 2H, arom.), 7.38 (d, *J* = 7.0 Hz, 2H, arom.), 7.42 (d, *J* = 8.8 Hz, 2H, arom.), 7.45 (s, 1H, H-3), 7.50 (d, *J* = 8.3 Hz, 2H, arom.), 7.55-7.57 (m, 3H, H-6, arom.), 7.61 (br. s, 1H, NH), 7.77 (br. s, 1H, NH), 9.94 (s, 1H, NH) ppm. ^{13}C NMR (125 MHz, DMSO-*d$_6$*): δ 19.45, 36.80, 127.63, 128.34, 128.37, 128.92, 129.04, 129.39, 129.48, 129.65, 130.04, 130.36, 130.39, 130.84, 132.43, 133.64, 135.07, 135.48, 135.90, 136.45, 137.00, 139.83, 153.70, 154.51 ppm. ESI-MS *m/z* calcd for $C_{30}H_{26}Cl_2N_4O_2S_2$ [M+H$^+$] 609.1 found 609.0. Anal. calcd. for $C_{30}H_{26}Cl_2N_4O_2S_2$ (609.59); C, 59.11; H, 4.30; N, 9.19. Found: C, 59.55; H, 4.33; N, 9.28.

*3-(2-Benzylthio-4-chloro-5-methylbenzenesulfonyl)-2-[3-(4-nitrophenyl)-1-phenylprop-2-enylideneamino]guanidine (**13**)*

Starting from **1** (0.192 g) and 4-nitrochalcone (0.127 g) with stirring for 1 h, the title compound **13** was obtained after crystallization from EtOH (0.239 g, 77%): m.p. 198−201 °C; IR (KBr): 3475, 3341, 3235 (NH), 1627 (NH), 1596, 1563 (C=N, C=C), 1522 (NO_2), 1341 (NO_2, SO_2), 1125 (SO_2) cm^{-1}, ^{1}H NMR (200 MHz, DMSO-*d$_6$*): δ 2.30 (s, 3H, CH_3), 4.31 (s, 2H, CH_2), 6.54 (d, *J* = 16.2 Hz, 1H, CH=C), 7.14–7.29 (m, 3H, C=CH, arom.), 7.33–7.40 (m, 2H, arom.), 7.42–7.43 (m, 4H, arom.), 7.49 (s, 1H, H-3), 7.57–7.60 (m, 4H, NH, H-6, arom.), 7.72–7.84 (m, 3H, NH, arom.), 8.24 (d, *J* = 8.67 Hz, 2H, arom) ppm. ^{13}C NMR (125 MHz, DMSO-*d$_6$*): δ 19.45, 36.83, 124.57, 127.63, 128.31, 128.38, 128.40, 128.93, 129.66, 130.08, 130.41, 130.48, 130.55, 132.49, 132.96, 134.22, 135.89, 136.42, 137.05, 139.76, 142.79, 147.31, 153.10, 154.54 ppm. ESI-MS *m/z* calcd for $C_{30}H_{26}ClN_5O_4S_2$ [M+H$^+$] 620.1 found 619.9. Anal. calcd. for $C_{30}H_{26}ClN_5O_4S_2$ (620.14) ; C, 58.10; H, 4.23; N, 11.29. Found: C, 58.43; H, 4.57; N, 11.55.

*3-{4-Chloro-2-[(2-methylphenyl)methylthio]-5-methylbenzenesulfonyl}-2-[3-(2-hydroxyphenyl)-1-phenylprop-2-enylideneamino]guanidine (**14**)*

Starting from **2** (0.200 g) and 2-hydroxychalcone (0.112 g) with stirring for 2 h, the title compound **14** was obtained after crystallization from MeCN (0.076 g, 25%): m.p. 123–128 °C; IR (KBr):

3462, 3271, 3343 (OH, NH), 1635 (NH), 1541, 1498 (C=C, C=N), 1330, 1161 (SO_2) cm^{-1}; ^1H NMR (500 MHz, DMSO-d_6): δ 2.30 (s, 3H, CH_3), 2.36 (s, 3H, CH_3), 4.24 (s, 2H, CH_2), 6.54 (d, *J* = 16.6 Hz, 1H, CH=C), 6.81–6.85 (m, 2H, C=CH, arom.), 6.94–6.96 (m, 2H, arom.), 7.09–7.18 (m, 5H, arom.), 7.31 (d, *J* = 7.3 Hz, 1H, arom.), 7.42 (d, *J* = 7.4 Hz, 1H, arom.), 7.46 (s, 1H, H-3), 7.49–7.53 (m, 4H, H-6, arom.), 7.57 (br. s, 1H, NH), 7.66 (br. s, 1H, NH), 9.75 (br. s, 1H, OH), 9.81 (s, 1H, NH) ppm. ^{13}C NMR (125 MHz, DMSO-d_6): δ 19.17, 19.46, 35.41, 116.40, 119.91, 122.93, 126.49, 127.36, 127.96, 128.08, 128.39, 129.88, 130.14, 130.41, 130.47, 130.62, 130.79, 130.91, 131.13, 132.29, 132.99, 134.00, 136.57, 137.02, 137.68, 139.69, 154.34, 155.07, 155.79 ppm. ESI-MS *m/z* calcd for $C_{31}H_{29}ClN_4O_3S_2$ [M+H$^+$] 605.1 found 604.9. Anal. calcd. for $C_{31}H_{29}ClN_4O_3S_2$ (605.17); C, 61.53; H, 4.83; N, 9.26. Found: C, 61.24; H, 4.65; N, 9.21.

*3-{4-Chloro-2-[(2-methylphenyl)methylthio]-5-methylbenzenesulfonyl}-2-[3-(4-hydroxyphenyl)-1-phenylprop-2-enylideneamino]guanidine (**15**)*

Starting from **2** (0.200 g) and 4-hydroxychalcone (0.112 g) with stirring for 2.5 h, the title compound **15** was obtained after crystallization from MeCN (0.166 g, 55%): m.p. 220–225 °C; IR (KBr): 3415, 3301, 3262 (OH, NH), 1652 (NH), 1608, 1558, 1513, 1447 (C=C, C=N), 1340, 1167 (SO_2) cm^{-1}; ^1H NMR (500 MHz, DMSO-d_6): δ 2.30 (s, 3H, CH_3), 2.36 (s, 3H, CH_3), 4.23 (s, 2H, CH_2), 6.20 (d, *J* = 16.1 Hz, 1H, CH=C), 6.76 (d, *J* = 8.3 Hz, 2H, arom.), 6.90 (s, 1H, arom.), 6.94–6.95 (m, 2H, C=CH, arom.), 7.08–7.16 (m, 3H, arom.), 7.28–7.32 (m, 3H, arom.), 7.46 (s, 1H, H-3), 7.49–7.54 (m, 4H, H-6, arom.), 7.57 (br. s, 1H, NH), 7.66 (br. s, 1H, NH), 9.77 (br. s, 1H, OH), 9.80 (s, 1H, NH) ppm. ^{13}C NMR (125 MHz, DMSO-d_6): δ 19.16, 19.46, 35.39, 116.24, 125.27, 126.49, 127.17, 128.01, 128.04, 128.32, 129.00, 129.89, 130.13, 130.41, 130.63, 130.78, 131.16, 132.26, 133.98, 136.57, 137.02, 137.39, 137.70, 139.66, 154.34, 154.6, 158.85 ppm. ESI-MS *m/z* calcd for $C_{31}H_{29}ClN_4O_3S_2$ [M+H$^+$] 605.1 found 605.0. Anal. calcd. for $C_{31}H_{29}ClN_4O_3S_2$ (605.17); C, 61.53; H, 4.83; N, 9.26. Found: C, 61.38; H, 4.79; N, 9.19.

*3-{4-Chloro-2-[(3-methylphenyl)methylthio]-5-methylbenzenesulfonyl}-2-[3-(2-hydroxyphenyl)-1-phenylprop-2-enylideneamino]guanidine (**16**)*

Starting from **3** (0.200 g) and 2-hydroxychalcone (0.112 g) with stirring for 3.5 h, the title compound **16** was obtained after crystallization from EtOH (0.118 g, 39%): m.p. 143–148 °C; IR (KBr): 3536, 3455, 3344 (OH, NH), 1636 (NH), 1546, 1510, 1456, 1422 (C=C, C=N), 1333, 1169 (SO_2) cm^{-1}; ^1H NMR (500 MHz, DMSO-d_6): δ 2.27 (s, 3H, CH_3), 2.28 (s, 3H, CH_3), 4.23 (s, 2H, CH_2), 6.58 (d, *J* = 16.1 Hz, 1H, CH=C), 6.80–6.85 (m, 2H, C=CH, arom.), 7.03 (m, 1H, arom.), 7.10–7.15 (m, 3H, arom.), 7.19–7.22 (m, 4H, arom.), 7.42 (d, *J* = 7.9 Hz, 1H, arom.), 7.45 (s, 1H, H-3), 7.54–7.56 (m, 5H, NH, H-6, arom.), 7.70 (br. s, 1H, NH), 9.82 (s, 1H, NH), 9.84 (br. s, 1H, OH) ppm. ^{13}C NMR (125 MHz, DMSO-d_6): δ 19.46, 21.43, 36.85, 116.42, 119.90, 122.89, 126.77, 127.35, 127.98, 128.27, 128.39, 128.43, 128.48, 128.82, 129.96, 130.23, 130.38, 130.43, 131.20, 132.34, 133.06, 136.06, 136.30, 136.96, 138.05, 139.83, 154.38, 155.11, 155.81 ppm. ESI-MS *m/z* calcd for $C_{31}H_{29}ClN_4O_3S_2$ [M+H$^+$] 605.1 found 604.9. Anal. calcd. for $C_{31}H_{29}ClN_4O_3S_2$ (605.17); C, 61.53; H, 4.83; N, 9.26. Found: C, 61.85; H, 4.71; N, 9.35.

*3-{4-Chloro-2-[(3-methylphenyl)methylthio]-5-methylbenzenesulfonyl}-2-[3-(4-hydroxyphenyl)-1-phenylprop-2-enylideneamino]guanidine (**17**)*

Starting from **3** (0.200 g) and 4-hydroxychalcone (0.112 g) with stirring for 1.5 h, the title compound **17** was obtained after crystallization from EtOH (0.206 g, 68%): m.p. 124–128 °C; IR (KBr): 3417, 3316, 3258 (OH, NH), 1648 (NH), 1607, 1585, 1546, 1513, 1444 (C=C, C=N), 1339, 1168 (SO_2) cm^{-1}; ^1H NMR (500 MHz, DMSO-d_6): δ 2.26 (s, 3H, CH_3), 2.28 (s, 3H, CH_3), 4.22 (s, 2H, CH_2), 6.25 (d, *J* = 16.1 Hz, 1H, CH=C), 6.75 (d, *J* = 8.8 Hz, 2H, arom.), 6.95 (d, *J* = 16.6 Hz, 1H, C=CH), 7.02 (s, 1H, arom.), 7.10–7.11 (m, 2H, arom.), 7.19–7.20 (m, 3H, arom.), 7.29 (d, *J* = 8.8 Hz, 2H, arom.), 7.52 (br. s, 1H, NH), 7.54–7.55 (m, 4H, H-6, arom.), 7.69 (br. s, 1H, NH), 9.80 (s, 1H, NH), 9.82 (br. s, 1H, OH) ppm. ^{13}C NMR (125 MHz, DMSO-d_6): δ 19.45, 21.42, 36.84, 116.24, 125.29, 126.78, 127.14, 127.17, 128.25, 128.36, 128.45, 128.81, 129.01, 129.97, 130.23, 130.38, 131.24, 132.32, 136.06, 136.30, 136.95, 137.45, 138.04, 139.83, 154.39, 154.67, 158.87 ppm. ESI-MS *m/z* calcd for $C_{31}H_{29}ClN_4O_3S_2$ [M+H$^+$] 605.1 found 604.9. Anal. calcd. for $C_{31}H_{29}ClN_4O_3S_2$ (605.17); C, 61.53; H, 4.83; N, 9.26. Found: C, 61.79; H, 4.53; N, 9.54.

*3-{4-Chloro-2-[(4-methylphenyl)methylthio]-5-methylbenzenesulfonyl}-2-(1,3-diphenylprop-2-enylideneamino)guanidine (**18**)*

Starting from **4** (0.200 g) and *trans*-chalcone (0.104 g) with stirring for 3 h, the title compound **18** was obtained after crystallization from MeCN (0.194 g, 66%): m.p. 172–176 °C; IR (KBr): 3441, 3316 (NH), 1610 (NH), 1522, 1493, 1448 (C=N, C=C), 1340, 1167 (SO_2) cm^{-1}; ^1H NMR (500 MHz, DMSO-d_6): δ 2.10 (s, 3H, CH_3), 2.27 (s, 3H, CH_3), 4.22 (s, 2H, CH_2), 6.33 (d, *J* = 16.3 Hz, 1H, CH=C), 7.03–7.04 (m, 2H, arom.), 7.10 (d, *J* = 7.8 Hz, 2H, arom.), 7.17 (d, *J* =16.3 Hz, 1H, C=CH), 7.26 (d, *J* = 7.8 Hz, 2H, arom.), 7.30–7.32 (m, 1H, arom), 7.35–7.80 (t, 2H, arom.), 7.45–7.47 (m, 3H, H-3, arom.), 7.53-7.60 (m, 5H, H-6, NH, arom.), 7.74 (br. s, 1H, NH), 9.92 (s, 1H, NH) ppm; ^{13}C NMR (125 MHz, DMSO-d_6): δ 19.69, 21.30, 36.87, 127.58, 128.44, 128.58, 128.99, 129.57, 129.63, 129.74, 129.83, 130.22, 130.53, 130.60, 131.18, 132.58, 133.43, 136.32, 136.41, 137.12, 137.15, 137.22, 140.05, 154.19, 154.79 ppm. ESI-MS *m*/*z* calcd for $C_{31}H_{29}ClN_4O_2S_2$ [M+H$^+$] 589.1 found 589.2. Anal. calcd. for $C_{31}H_{29}ClN_4O_2S_2$ (589.17); C, 63.20; H, 4.96; N, 9.51. Found: C, 62.92; H, 5.07; N, 9.37.

*3-{4-Chloro-2-[(4-methylphenyl)methylthio]-5-methylbenzenesulfonyl}-2-[3-(2-hydroxyphenyl)-1-phenylprop-2-enylideneamino]guanidine (**19**)*

Starting from **4** (0.200 g) and 2-hydroxychalcone (0.112 g) with stirring for 2 h, the title compound **19** was obtained after crystallization from EtOH (0.106 g, 35%): m.p. 120–125 °C; IR (KBr): 3453, 3263, 3183 (OH, NH), 1628 (NH), 1600, 1569, 1549, 1459 (C=C, C=N), 1321, 1158 (SO_2) cm^{-1}; ^1H NMR (500 MHz, DMSO-d_6): δ 2.14 (s, 3H, CH_3), 2.28 (s, 3H, CH_3), 4.23 (s, 2H, CH_2), 6.56 (d, *J* = 16.6 Hz, 1H, CH=C), 6.82–6.85 (m, 2H, arom.), 7.03–7.04 (m, 2H, arom.), 7.11–7.13 (m, 3H, arom.), 7.22 (d, *J* = 16.6 Hz, 1H, C=CH), 7.28 (d, *J* = 7.8 Hz, 2H, arom.), 7.43–7.46 (m, 2H, H-3, arom.), 7.53–7.55 (m, 4H, H-6, arom.), 7.58 (br. s, 1H, NH), 7.69 (br. s, 1H, NH), 9.82 (s, 1H, NH), 9.83 (br. s, 1H, OH) ppm. ^{13}C NMR (125 MHz, DMSO-d_6): δ 19.45, 21.03, 36.59, 116.39, 119.88, 122.90, 127.37, 128.08, 128.14, 128.40, 129.52, 129.57, 129.91, 130.17, 130.35, 130.41, 131.20, 132.29, 132.97, 133.18, 136.16, 136.90, 136.94, 139.84, 154.46, 155.07, 155.81 ppm. ESI-MS *m*/*z* calcd for $C_{31}H_{29}ClN_4O_3S_2$ [M+H$^+$] 605.1 found 604.9. Anal. calcd. for $C_{31}H_{29}ClN_4O_3S_2$ (605.17); C, 61.53; H, 4.83; N, 9.26. Found: C, 61.28; H, 4.61; N, 9.18.

*3-{4-Chloro-2-[(4-methylphenyl)methylthio]-5-methylbenzenesulfonyl}-2-[3-(4-hydroxyphenyl)-1-phenylprop-2-enylideneamino]guanidine (**20**)*

Starting from **4** (0.200 g) and 4-hydroxychalcone (0.112 g) with stirring for 1 h, the title compound **20** was obtained after crystallization from MeCN (0.139 g, 46%): m.p. 159–164 °C; IR (KBr): 3343, 3321 (OH, NH), 1608 (NH), 1550, 1513, 1446 (C=N, C=C), 1340, 1169 (SO_2) cm^{-1}; ^1H NMR (500 MHz, DMSO-d_6): δ 2.12 (s, 3H, CH_3-Ph), 2.26 (s, 3H, CH_3), 4.21 (s, 2H, CH_2), 6.22 (d, *J* = 16.5 Hz, 1H, CH=C), 6.73–6.75 (m, 2H, arom.), 6.94–6.98 (d, *J* = 16.5 Hz, 1H, C=CH), 7.01 (m, 2H, arom.), 7.08–7.10 (d, *J* = 7.6 Hz, 2H, arom.), 7.25–7.29 (m, 4H, arom.), 7.44 (s, 1H, H-3), 7.52 (m, 5H, H-6, arom., NH), 7.68 (br. s, 1H, NH), 9.79 (s, 2H, OH, NH) ppm. ^{13}C NMR (125 MHz, DMSO-d_6): δ 19.44, 21.07, 36.57, 116.22, 125.39, 127.16, 128.14, 128.33, 129.01, 129.49, 129.57, 129.92, 130.17, 130.33, 131.23, 132.27, 133.20, 136.15, 136.87, 136.92, 137.37, 139.85, 154.46, 154.63, 158.84 ppm. ESI-MS *m*/*z* calcd for $C_{31}H_{29}ClN_4O_3S_2$ [M+H$^+$] 605.1 found 605.0. Anal. calcd. for $C_{31}H_{29}ClN_4O_3S_2$ (605.17); C, 61.53; H, 4.83; N, 9.26. Found: C, 61.88; H, 4.93; N, 9.39.

*3-{4-Chloro-2-[(4-methylphenyl)methylthio]-5-methylbenzenesulfonyl}-2-[3-(4-methoxyphenyl)-1-phenylprop-2-enylideneamino]guanidine (**21**)*

Starting from **4** (0.200 g) and 4-methoxychalcone (0.119 g) with stirring for 1 h, the title compound **21** was obtained after crystallization from MeCN (0.158 g, 51%): m.p. 198–202 °C; IR (KBr): 3476, 3245 (NH), 1632 (NH), 1605, 1570, 1550, 1510, 1441 (C=N, C=C), 1342, 1170 (SO_2) cm^{-1}; ^1H NMR (500 MHz, DMSO-d_6): δ 2.12 (s, 3H, CH_3), 2.27 (s, 3H, CH_3), 3.76 (s, 3H, OCH_3), 4.22 (s, 2H, CH_2), 6.28 (d, *J* = 16.1 Hz, 1H, CH=C), 6.92 (d, *J* = 8.7 Hz, 2H, arom.), 7.01–7.05 (m, 3H, C=CH, arom.), 7.10 (d, *J* =

7.8 Hz, 2H, arom.), 7.26 (d, *J* = 7.3 Hz, 2H, arom.), 7.41 (d, *J* = 8.8 Hz, 2H, arom.), 7.45 (s, 1H, H-3), 7.52–7.54 (m, 5H, H-6, NH, arom.), 7.70 (br. s, 1H, NH), 9.84 (s, 1H, NH) ppm. ^{13}C NMR (125 MHz, DMSO-*d*$_6$): δ 19.44, 21.09, 36.60, 55.66, 114.83, 126.41, 128.16, 128.33, 128.73, 128.88, 129.50, 129.58, 129.94, 130.20, 130.35, 131.14, 132.29, 133.20, 136.17, 136.81, 136.88, 136.95, 139.84, 154.39, 154.49, 160.30 ppm. ESI-MS *m/z* calcd for $C_{32}H_{31}ClN_4O_3S_2$ [M+H$^+$] 619.2 found 619.0. Anal. calcd. for $C_{32}H_{31}ClN_4O_3S_2$ (619.20); C, 62.07; H, 5.05; N, 9.05. Found: C, 61.91; H, 4.87; N, 8.79.

*3-{4-Chloro-2-[(4-methylphenyl)methylthio]-5-methylbenzenesulfonyl}-2-[3-(4-chlorophenyl)-1-phenylprop-2-enylideneamino]guanidine (**22**)*

Starting from **4** (0.200 g) and 4-chlorochalcone (0.121 g) with stirring for 4 h, the title compound **22** was obtained after crystallization from EtOH (0.122 g, 39%): m.p. 172–177 °C; IR (KBr): 3266 (NH), 1661, 1633 (NH), 1561, 1491, 1449 (C=C, C=N), 1328, 1167 (SO$_2$) cm^{-1}; ^{1}H NMR (500 MHz, DMSO-*d*$_6$): δ 2.28 (s, 3H, CH$_3$), 2.29 (s, 3H, CH$_3$), 4.23 (s, 2H, CH$_2$), 6.35 (d, *J* = 16.6 Hz, 1H, CH=C), 7.10–7.12 (m, 3H, arom.), 7.19 (d, *J* = 16.6 Hz, 1H, C=CH), 7.27 (d, *J* = 7.8 Hz, 2H, arom.), 7.42 (d, *J* = 8.3 Hz, 2H, arom.), 7.47–7.48 (m, 3H, H-3, arom.), 7.52 (s, 1H, arom.), 7.53–7.55 (m, 3H, arom.), 7.58 (s, 1H, H-6), 7.60 (br. s, 1H, NH), 7.76 (br. s, 1H, NH), 9.95 (br. s, 1H, NH) ppm. ^{13}C NMR (125 MHz, DMSO-*d*$_6$): δ 19.44, 21.08, 36.64, 125.95, 128.21, 128.33, 128.59, 129.02, 129.38, 129.49, 129.59, 129.98, 130.81, 132.35, 133.18, 133.62, 135.09, 135.35, 136.16, 136.87, 137.00, 138.33, 153.66, 154.55 ppm. ESI-MS *m/z* calcd for $C_{31}H_{28}Cl_2N_4O_2S_2$ [M+H$^+$] 623.1 found 623.0. Anal. calcd. for $C_{31}H_{28}Cl_2N_4O_2S_2$ (623.62); C, 59.71; H, 4.53; N, 8.98. Found: C, 59.62; H, 4.38; N, 9.18.

*3-{4-Chloro-2-[(4-methylphenyl)methylthio]-5-methylbenzenesulfonyl}-2-[3-(4-nitrophenyl)-1-phenylprop-2-enylideneamino]guanidine (**23**)*

Starting from **4** (0.200 g) and 4-nitrochalcone (0.126 g) with stirring for 3 h, the title compound **23** was obtained after crystallization from MeCN (0.209 g, 66%): m.p. 152–155 °C; IR (KBr): 3493, 3345, 3223 (NH), 1630 (NH), 1596, 1562, 1491 (C=N, C=C), 1525 (NO$_2$ asym.), 1342 (NO$_2$, SO$_2$), 1126 (SO$_2$) cm^{-1}; ^{1}H NMR (500 MHz, DMSO-*d*$_6$): δ 2.10 (s, 3H, CH$_3$), 2.27 (s, 3H, CH$_3$), 4.22 (s, 2H, CH$_2$), 6.50 (d, *J* = 16.4 Hz, 1H, CH=C), 7.04-7.10 (m, 4H, arom.), 7.25 (d, *J* = 7.3 Hz, 2H, arom.), 7.36 (d, *J* = 16.4 Hz, 1H, C=CH), 7.46 (s, 1H, H-3), 7.53–7.54 (m, 2H, arom.), 7.58 (s, 1H, H-6), 7.67 (br. s, 1H, NH), 7.76-7.77 (m, 3H, arom.), 7.80 (br. s, 1H, NH), 8.21 (d, *J* = 8.3 Hz, 2H, arom.), 10.06 (s, 1H, NH) ppm. ^{13}C NMR (125 MHz, DMSO-*d*$_6$): δ 19.44, 21.09, 36.64, 124.57, 128.24, 128.30, 128.36, 129.49, 129.61, 130.04, 130.38, 130.45, 130.51, 132.41, 133.07, 133.18, 134.10, 136.16, 136.88, 137.05, 139.70, 142.82, 147.29, 153.06, 154.56 ppm. ESI-MS *m/z* calcd for $C_{31}H_{28}ClN_5O_4S_2$ [M+H$^+$] 634.1 found 633.9. Anal. calcd. for $C_{31}H_{28}ClN_5O_4S_2$ (634.17); C, 58.71; H, 4.45; N, 11,04. Found: C, 58.85; H, 4.60; N, 11.04.

*3-{4-Chloro-2-[(2-chlorophenyl)methylthio]-5-methylbenzenesulfonyl}-2-[3-(2-hydroxyphenyl)-1-phenylprop-2-enylideneamino]guanidine (**24**)*

Starting from **5** (0.210 g) and 2-hydroxychalcone (0.112 g) with stirring for 4 h, the title compound **24** was obtained after crystallization from acetone (0.118 g, 38%): m.p. 123–127 °C; IR (KBr): 3460, 3340, 3271 (OH, NH), 1639 (NH), 1605, 1541, 1501, 1458 (C=C, C=N), 1332, 1161 (SO$_2$) cm^{-1}; ^{1}H NMR (500 MHz, DMSO-*d*$_6$): δ 2.30 (s, 3H, CH$_3$), 4.33 (s, 2H, CH$_2$), 6.58 (d, *J* = 16.6 Hz, 1H, CH=C), 6.80–6.85 (m, 2H, arom.), 7.10–7.15 (m, 3H, arom.), 7.18 (d, *J* = 16.6 Hz, 1H, C=CH), 7.25–7.32 (m, 2H, arom.), 7.39 (s, 1H, H-3), 7.41–7.49 (m, 3H, arom.), 7.55–7.57 (m, 5H, H-6, NH, arom.), 7.68 (br. s, 1H, NH), 9.79 (br. s, 1H, OH), 9.82 (s, 1H, NH) ppm. ^{13}C NMR (125 MHz, DMSO-*d*$_6$): δ 19.49, 35.01, 116.42, 119.91, 122.89, 127.41, 127.89, 127.97, 128.43, 128.71, 129.77, 129.92, 129.97, 130.23, 130.44, 130.53, 131.19, 132.01, 132.90, 133.09, 133.99, 134.12, 135.31, 137.00, 140.27, 154.37, 155.06, 155.81 ppm. ESI-MS *m/z* calcd for $C_{31}H_{28}ClN_5O_4S_2$ [M+H$^+$] 625.1 found 625.1. Anal. calcd. for $C_{31}H_{28}ClN_5O_4S_2$ (625.59); C, 57.60; H, 4.19; N, 8.96. Found: C, 57.32; H, 3.95; N, 8.82.

*3-{4-Chloro-2-[(2-chlorophenyl)methylthio]-5-methylbenzenesulfonyl}-2-[3-(4-hydroxyphenyl)-1-phenylprop-2-enylideneamino]guanidine (**25**)*

Starting from **5** (0.210 g) and 4-hydroxychalcone (0.112 g) with stirring for 2 h, the title compound **25** was obtained after crystallization from MeCN (0.128 g, 41%): m.p. 207–212 °C; IR (KBr): 3410, 3319, 3284 (OH, NH), 1651 (NH), 1607, 1548, 1513, 1444 (C=C, C=N), 1340, 1167 (SO_2) cm^{-1}; ^{1}H NMR (500 MHz, DMSO-d_6): δ 2.30 (s, 3H, CH_3), 4.33 (s, 2H, CH_2), 6.25 (d, *J* = 16.6 Hz, 1H, CH=C), 6.75 (d, *J* = 8.3 Hz, 2H, arom.), 6.94 ((d, *J* = 16.1 Hz, 1H, C=CH), 7.09–7.11 (m, 2H, arom.), 7.24–7.31 (m, 4H, arom.), 7.39 (s, 1H, H-3), 7.44–7.48 (m, 3H, arom.), 7.54–7.55 (m, 3H, NH, arom.), 7.57 (s, 1H, H-6), 7.68 (br. s, 1H, NH), 9.78 (br. s, 1H, OH), 9.80 (s, 1H, NH) ppm. ^{13}C NMR (125 MHz, DMSO-d_6): δ 19.49, 35.00, 125.25, 127.14, 127.88, 128.36, 128.67, 129.03, 129.75, 129.91, 129.97, 130.20, 130.52, 131.23, 132.00, 132.88, 133.99, 134.10, 135.30, 136.99, 137.47, 140.26, 154.37, 158.87 ppm. ESI-MS *m/z* calcd for $C_{31}H_{28}ClN_5O_4S_2$ [M+H$^+$] 625.1 found 624.9. Anal. calcd. for $C_{30}H_{26}Cl_2N_4O_3S_2$ (625.59); C, 57.60; H, 4.19; N, 8.96. Found: C, 57.84; H, 4.40; N, 8.68.

*3-{4-Chloro-2-[(3-chlorophenyl)methylthio]-5-methylbenzenesulfonyl}-2-[3-(2-hydroxyphenyl)-1-phenylprop-2-enylideneamino]guanidine (**26**)*

Starting from **6** (0.210 g) and 2-hydroxychalcone (0.112 g) with stirring for 5 h, the title compound **26** was obtained after crystallization from MeCN (0.118 g, 38%): m.p. 104–108 °C; IR (KBr): 3461, 3342 (OH, NH), 1618 (NH), 1545, 1456 (C=C, C=N), 1339, 1162 (SO_2) cm^{-1}; ^{1}H NMR (500 MHz, DMSO-d_6): δ 2.28 (s, 3H, CH_3), 4.31 (s, 2H, CH_2), 6.60 (d, *J* = 16.6 Hz, 1H, CH=C), 6.80– 6.84 (m, 2H, arom.), 7.12–7.15 (t, 1H, arom.), 7.18–7.23 (m, 3H, C=CH, arom.), 7.28 (d, *J* = 7.3 Hz, 1H, arom.), 7.32–7.37 (m, 2H, arom.), 7.41–7.46 (m, 3H, H-3, arom.), 7.53 (s, 1H, H-6), 7.57–7.59 (m, 4H, NH, arom.), 7.71 (br. s, 1H, NH), 9.82 (m, 2H, OH, NH) ppm. ^{13}C NMR (125 MHz, DMSO-d_6): δ 19.48, 35.97, 116.41, 119.89, 122.88, 127.37, 127.66, 127.94, 128.26, 128.44, 128.54, 129.37, 130.00, 130.27, 130.33, 130.45, 130.76, 131.23, 132.68, 133.16, 133.42, 135.22, 136.98, 139.33, 140.08, 154.35, 155.20, 155.82ppm. ESI-MS *m/z* calcd for $C_{31}H_{28}ClN_5O_4S_2$ [M+H$^+$] 625.1 found 624.8. Anal. calcd. for $C_{30}H_{26}Cl_2N_4O_3S_2$ (625.59); C, 57.60; H, 4.19; N, 8.96. Found: C, 57.52; H, 4.29; N, 9.02.

*3-{4-Chloro-2-[(3-chlorophenyl)methylthio]-5-methylbenzenesulfonyl}-2-[3-(4-hydroxyphenyl)-1-phenylprop-2-enylideneamino]guanidine (**27**)*

Starting from **6** (0.210 g) and 4-hydroxychalcone (0.112 g) with stirring for 7 h, the title compound **27** was obtained after crystallization from MeCN (0.166 g, 53%): m.p. 196–211 °C; IR (KBr): 3414, 3313 (OH, NH), 1647 (NH), 1585, 1546, 1491, 1443 (C=C, C=N), 1339, 1168 (SO_2) cm^{-1}; ^{1}H NMR (500 MHz, DMSO-d_6): δ 2.28 (s, 3H, CH_3), 4.31 (s, 2H, CH_2), 6.27 (d, *J* = 16.1 Hz, 1H, CH=C), 6.75 (d, *J* = 8.8 Hz, 2H, arom.), 6.96 (d, *J* = 16.6 Hz, 1H, C=CH), 7.17–7.18 (m, 2H, arom.), 7.27–7.30 (m, 3H, arom.), 7.32–7.37 (m, 2H, arom.), 7.44–7.45 (m, 2H, H-3, arom.), 7.52–7.53 (m, 2H, NH, arom.), 7.57–7.58 (m, 3H, H-6, arom.), 7.71 (br. s, 1H, NH), 9.81 (m, 2H, OH, NH) ppm. ^{13}C NMR (125 MHz, DMSO-d_6): δ 19.47, 35.97, 116.22, 125.24, 127.13, 127.65, 128.26, 128.36, 128.49, 129.04, 129.37, 130.00, 130.26, 130.42, 130.75, 131.26, 132.66, 133.41, 135.22, 136.96, 137.57, 139.31, 140.08, 154.35, 154.78, 158.88 ppm. ESI-MS *m/z* calcd for $C_{31}H_{28}ClN_5O_4S_2$ [M+H$^+$] 625.1 found 625.0. Anal. calcd. for $C_{30}H_{26}Cl_2N_4O_3S_2$ (625.59); C, 57.60; H, 4.19; N, 8.96. Found: C, 57.29; H, 3.82; N, 8.64.

*3-{4-Chloro-2-[(4-chlorophenyl)methylthio]-5-methylbenzenesulfonyl}-2-(1,3-diphenylprop-2-enylideneamino]guanidine (**28**)*

Starting from **7** (0.210 g) and *trans*-chalcone (0.104 g), with stirring for 2 h, the title compound **28** was obtained after crystallization from MeCN (0.167 g, 55%): m.p. 184–188 °C; IR (KBr): 3444, 3336, 3313 (NH), 1610 (NH), 1521, 1491, 1448 (C=N, C=C), 1341, 1129 (SO_2) cm^{-1}; ^{1}H NMR (500 MHz, DMSO-d_6): δ 2.26 (s, 3H, CH_3), 4.29 (s, 2H, CH_2), 6.36 (d, *J* = 16.6 Hz, 1H, CH=C), 7.13–7.19 (m, 3H, C=CH, arom.), 7.29–7.31 (m, 1H, arom.), 7.34–7.37 (m, 4H, arom.), 7.38 (d, *J* = 8.3 Hz, 2H, arom.), 7.46–7.47 (m, 3H, H-3, arom.), 7.53–7.57 (m, 4H, H-6, arom.), 7.63 (br. s, 1H, NH), 7.75 (br. s, 1H, NH), 9.93 (s, 1H, NH) ppm. ^{13}C NMR (125 MHz, DMSO-d_6): δ 19.46, 35.93, 127.40, 128.36, 128.61, 128.77, 128.86, 128.92, 129.35, 130.04, 130.36, 130.39, 130.98, 131.42 132.29, 132.69, 135.33, 135.74, 136.06, 136.99, 137.15, 140.12,

154.04, 154.51ppm. ESI-MS *m/z* calcd for $C_{30}H_{26}Cl_2N_4O_2S_2$ [M+H$^+$] 609.1 found 608.9. Anal. calcd. for $C_{30}H_{26}Cl_2N_4O_2S_2$ (609.59); C, 59.11; H, 4.30; N, 9.19. Found: C, 59.39; H, 3.99; N, 9.49.

*3-{4-Chloro-2-[(4-chlorophenyl)methylthio]-5-methylbenzenesulfonyl}-2-[3-(2-hydroxyphenyl)-1-phenylprop-2-enylideneamino]guanidine (**29**)*

Starting from **7** (0.210 g) and 2-hydroxychalcone (0.112 g) with stirring for 5 h, the title compound **29** was obtained after crystallization from EtOH (0.113 g, 36%): m.p. 126–131 °C; IR (KBr): 3456, 3414, 3346, 3285 (NH), 1653 (NH), 1567, 1549, 1457 (C=C, C=N), 1333, 1160 (SO_2) cm^{-1}; ^{1}H NMR (500 MHz, DMSO-*d*$_6$): δ 2.28 (s, 3H, CH_3), 4.30 (s, 2H, CH_2), 6.60 (d, *J* = 16.6 Hz, 1H, CH=C), 6.80–6.84 (m, 2H, arom.), 7.11– 7.17 (m, 3H, arom.), 7.22 (d, *J* = 16.1 Hz, 1H, C=CH), 7.36 (d, *J* = 8.3 Hz, 2H, arom.), 7.40–7.44 (m, 3H, arom.), 7.46 (s, 1H, H-3), 7.52 (m, 1H, arom.), 7.57–7.58 (m, 3H, H-6, arom.), 7.61 (br. s, 1H, NH), 7.71 (br. s, 1H, NH), 9.81 (s, 1H, NH), 9.84 (br. s, 1H, OH) ppm. ^{13}C NMR (125 MHz, DMSO-*d*$_6$): δ 19.47, 35.85, 116.38 119.86, 122.89, 127.43, 127.92, 128.43, 128.59, 128.75, 128.85, 130.00, 130.26, 130.38, 130.43, 131.27, 131.39, 132.27, 132.64, 135.27, 135.80, 136.94, 140.17, 154.41, 155.17, 155.83 ppm. ESI-MS *m/z* calcd for $C_{31}H_{28}ClN_5O_4S_2$ [M+H$^+$] 625.1 found 624.9. Anal. calcd. for $C_{30}H_{26}Cl_2N_4O_3S_2$ (625.59); C, 57.60; H, 4.19; N, 8.96. Found: C, 57.91; H, 4.33; N, 9.20.

*3-{4-Chloro-2-[(4-chlorophenyl)methylthio]-5-methylbenzenesulfonyl}-2-[3-(4-hydroxyphenyl)-1-phenylprop-2-enylideneamino]guanidine (**30**)*

Starting from **7** (0.210 g) and 4-hydroxychalcone (0.112 g) with stirring for 2 h, the title compound **30** was obtained after crystallization from MeCN (0.175 g, 56%): m.p. 198–203 °C; IR (KBr): 3417, 3266 (OH, NH), 3022 (CH), 1649 (NH), 1607, 1545, 1512, 1492 (C=N, C=C), 1339, 1167 (SO_2) cm^{-1}; ^{1}H NMR (500 MHz, DMSO-*d*$_6$): δ 2.26 (s, 3H, CH_3), 4.28 (s, 2H, CH_2), 6.25 (d, *J* = 16.4 Hz, 1H, CH=C), 6.73 (d, *J* = 8.3 Hz, 2H, arom.), 6.95 (d, *J* = 16.4 Hz, 1H, H-5 pirazolina), 7.11–7.13 (m, 2H, arom.), 7.28 (d, *J* = 8.3 Hz, 2H, arom.), 7.33 (d, *J* = 8.3 Hz, 2H, arom.), 7.40 (d, *J* = 8.3 Hz, 2H, arom.), 7.45 (s, 1H, H-3), 7.51–7.55 (m, 6H, H-6, NH, arom.), 7.70 (br. s, 1H, NH), 9.78 (br. s, 1H, OH), 9.82 (s, 1H, NH) ppm. ^{13}C NMR (125 MHz, DMSO-*d*$_6$): δ 19.47, 35.87, 116.19, 125.24, 127.15, 128.35, 128.57, 128.85, 129.07, 130.00, 130.24, 130.38, 131.27, 131.40, 132.27, 132.63, 135.30, 135.77, 136.94 137.62, 140.18, 154.43, 154.73, 158.86 ppm. ESI-MS *m/z* calcd for $C_{31}H_{28}ClN_5O_4S_2$ [M+H$^+$] 625.1 found 625.0. Anal. calcd. for $C_{30}H_{26}Cl_2N_4O_3S_2$ (625.59); C, 57.60; H, 4.19; N, 8.96. Found: C, 57.46; H, 4.21; N, 8.74.

*3-{4-Chloro-2-[(4-chlorophenyl)methylthio]-5-methylbenzenesulfonyl}-2-[3-(4-methoxyphenyl)-1-phenylprop-2-enylideneamino]guanidine (**31**)*

Starting from **7** (0.210 g) and 4-methoxychalcone (0.119 g) with stirring for 1 h, the title compound **31** was obtained after crystallization from MeCN (0.208 g, 68%): m.p. 197–202 °C; IR (KBr): 3456, 3340, 3235 (NH), 1616 (NH), 1565, 1510, 1491 (C=N, C=C), 1341, 1174 (SO_2) cm^{-1}; ^{1}H NMR (500 MHz, DMSO-*d*$_6$): δ 2.26 (s, 3H, CH_3), 3.75 (s, 3H, OCH_3), 4.29 (s, 2H, CH_2), 6.31 (d, *J* = 16.6 Hz, 1H, CH=C), 6.91 (d, *J* = 8.3 Hz, 2H, arom.), 7.03 (d, *J* = 16.6 Hz, 1H, C=CH), 7.12 (m, 2H, arom.), 7.34 (d, *J* = 8.3 Hz, 2H, arom.), 7.39–7.41 (m, 4H, arom.), 7.45 (s, 1H, H-3), 7.52–7.58 (m, 5H, H-6, NH, arom.), 7.73 (br. s, 1H, NH), 9.86 (s, 1H, NH) ppm; ^{13}C NMR (125 MHz, DMSO-*d*$_6$): δ 19.71, 36.16, 55.90, 115.03, 126.51, 128.59, 128.82, 128.96, 129.09, 129.16, 130.25, 130.51, 130.62, 131.42, 131.65, 132.53, 132.89, 135.22, 135.55, 136.00, 137.19, 137.29, 140.41, 154.70, 154.72, 160.56 ppm. ESI-MS *m/z* calcd for $C_{31}H_{28}Cl_2N_4O_3S_2$ [M+H$^+$] 639.1 found 639.0. Anal. calcd. for $C_{31}H_{28}Cl_2N_4O_3S_2$ (639.61); C, 58.21; H, 4.41; N, 8.76. Found: C, 58.50; H, 4.73; N, 9.05.

*3-{4-Chloro-2-[(4-chlorophenyl)methylthio]-5-methylbenzenesulfonyl}-2-[5-(4-chlorophenyl)-1-phenylprop-2-enylideneamino]guanidine (**32**)*

Starting from from **7** (0.210 g) and 4-chlorochalcone (0.121 g) with stirring for 2.5 h, the title compound **32** was obtained after crystallization from MeCN (0.071 g, 22%): m.p. 162–167 °C; IR (KBr): 3462, 3442, 3335, 3298 (NH), 1610 (NH), 1523, 1489, 1450 (C=C, C=N), 1342, 1176 (SO_2) cm^{-1}; ^{1}H NMR

(500 MHz, DMSO-d_6): δ 2.28 (s, 3H, CH_3), 4.30 (s, 2H, CH_2), 6.38 (d, *J* = 16.6 Hz, 1H, CH=C), 7.12–7.14 (m, 2H, arom.), 7.18 (d, *J* = 16.6 Hz, 1H, C=CH), 7.35 (d, *J* = 8.3 Hz, 2H, arom.), 7.40–7.43 (m, 4H, arom.), 7.47 (s, 1H, H-3), 7.50 (d, *J* = 8.3 Hz, 2H, arom.), 7.50–7.57 (m, 4H, H-6, arom.), 7.63 (br. s, 1H, NH), 7.78 (br. s, 1H, NH), 9.97 (br. s, 1H, NH) ppm. ^{13}C NMR (125 MHz, DMSO-d_6): δ 19.46, 35.95, 128.36, 128.59, 128.87, 129.07, 129.33, 129.43, 130.05, 130.39, 130.44, 130.84, 131.43, 132.31, 132.71, 133.63, 135.08, 135.35, 135.60, 135.70, 137.01, 140.09, 153.74, 154.53 ppm. ESI-MS *m/z* calcd for $C_{30}H_{25}Cl_3N_4O_2S_2$ [M+H$^+$] 643.1 found 643.0. Anal. calcd. for $C_{30}H_{25}Cl_3N_4O_2S_2$ (644.03); C, 55.95; H, 3.91; N, 8.70. Found: C, 55.68; H, 4.04; N, 9.03.

*3-{4-Chloro-2-[(4-chlorophenyl)methylthio]-5-methylbenzenesulfonyl}-2-[3-(4-nitrophenyl)-1-phenylprop-2-enylideneamino]guanidine (**33**)*

Starting from from **7** (0.210 g) and 4-nitrochalcone (0.127 g) with stirring for 1 h, the title compound **33** was obtained after crystallization from MeCN (0.160 g, 49%): m.p. 206–211 °C; IR (KBr): 3443, 3335, 3296 (NH), 1613 (NH), 1520 (NO_2), 1341, 1129 (NO_2, SO_2), 1129 (SO_2) cm^{-1}; ^{1}H NMR (500 MHz, DMSO-d_6): δ 2.27 (s, 3H, CH_3), 4.29 (s, 2H, CH_2), 6.51 (d, *J* = 16.6 Hz, 1H, CH=C), 7.12–7.13 (m, 2H, arom.), 7.33–7.35 (m, 2H, C=CH, arom.), 7.38–7.40 (m, 3H, arom.), 7.47 (s, 1H, H-3), 7.57 (m, 4H, H-6, arom), 7.70 (br. s, 1H, NH), 7.75 (d, *J* = 8.8 Hz, 2H, arom.), 7.82 (br. s, 1H, NH), 8.20 (d, *J* = 8.3 Hz, 2H, arom.), 10.07 (s, 1H, NH) ppm. ^{13}C NMR (125 MHz, DMSO-d_6): δ 19.45, 36.00, 124.51, 128.35, 128.38, 128.62, 128.88, 130.09, 130.41, 130.50, 130.54, 131.44, 132.33, 132.76, 132.92, 134.32, 135.37, 135.67, 137.06, 140.02, 142.81, 147.29, 153.13, 154.56 ppm. ESI-MS *m/z* calcd for $C_{30}H_{25}Cl_2N_5O_4S_2$ [M+H$^+$] 654.1 found 654.0. Anal. calcd. for $C_{30}H_{25}Cl_2N_5O_4S_2$ (654.59); C, 55.05; H, 3.85; N, 10.70. Found: C, 54.77; H, 4.02; N, 10.55.

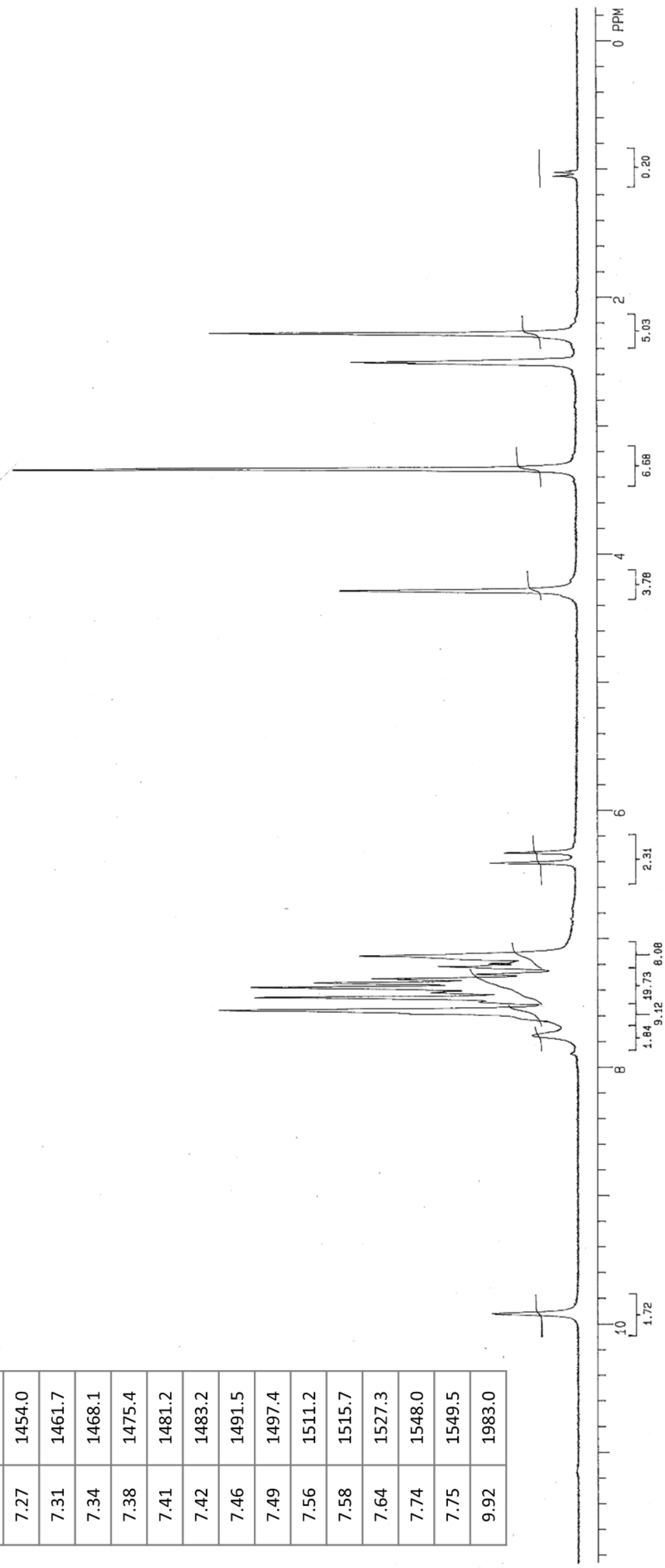

(ppm)	(Hz)
2.28	455.5
4.28	856.3
6.32	1264.9
6.41	1281.3
7.11	1422.0
7.12	1424.5
7.13	1426.0
7.15	1430.7
7.18	1435.5
7.19	1437.6
7.20	1440.7
7.21	1442.6
7.22	1444.4
7.27	1454.0
7.31	1461.7
7.34	1468.1
7.38	1475.4
7.41	1481.2
7.42	1483.2
7.46	1491.5
7.49	1497.4
7.56	1511.2
7.58	1515.7
7.64	1527.3
7.74	1548.0
7.75	1549.5
9.92	1983.0

Spectrum 1. ^{1}H NMR of compd **8** (200 MHz, DMSO-d_6).

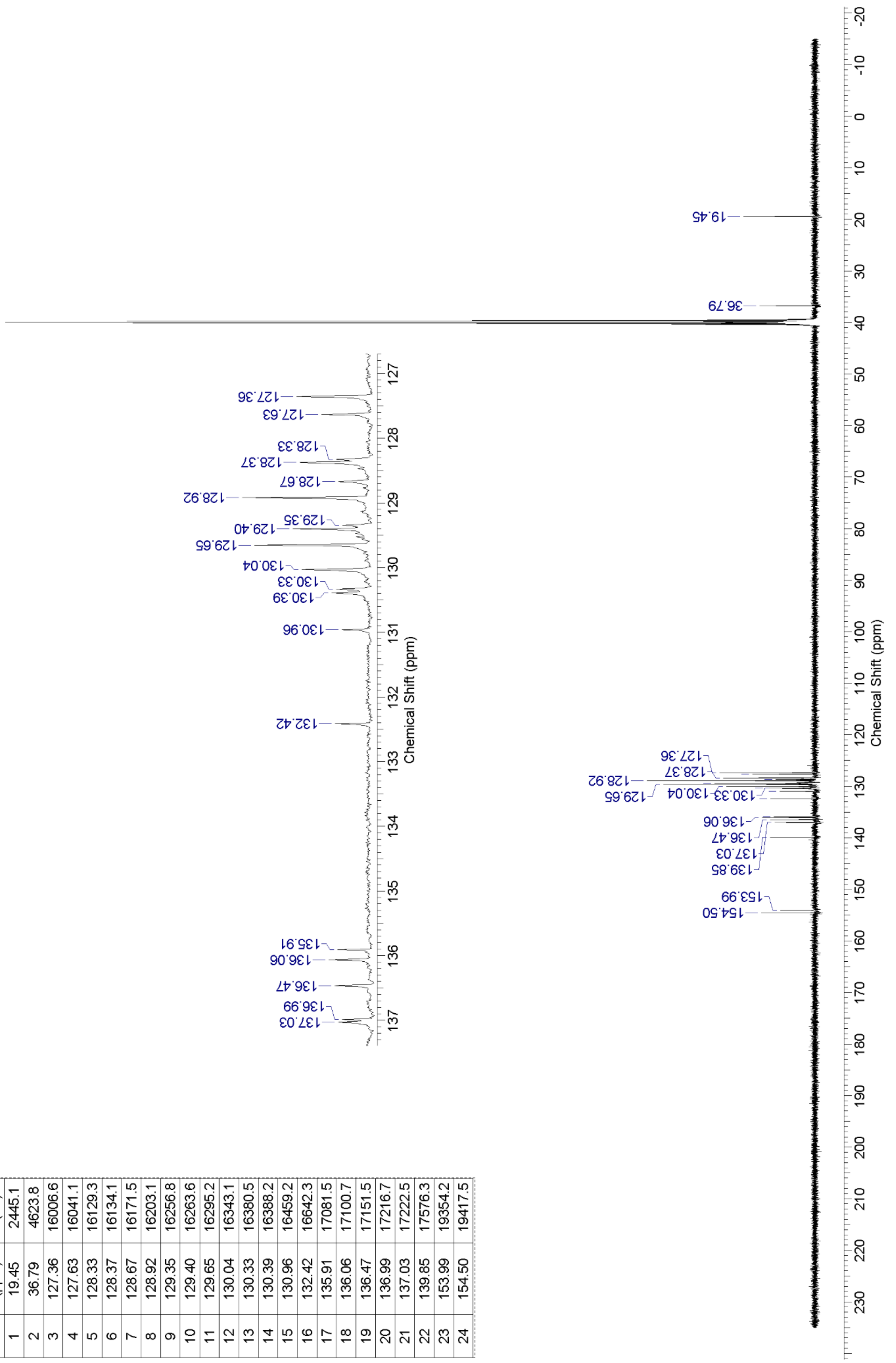

No.	(ppm)	(Hz)
1	19.45	2445.1
2	36.79	4623.8
3	127.36	16006.6
4	127.63	16041.1
5	128.33	16129.3
6	128.37	16134.1
7	128.67	16171.5
8	128.92	16203.1
9	129.35	16256.8
10	129.40	16263.6
11	129.65	16295.2
12	130.04	16343.1
13	130.33	16380.5
14	130.39	16388.2
15	130.96	16459.2
16	132.42	16642.3
17	135.91	17081.5
18	136.06	17100.7
19	136.47	17151.5
20	136.99	17216.7
21	137.03	17222.5
22	139.85	17576.3
23	153.99	19354.2
24	154.50	19417.5

Spectrum 2. ^{13}C NMR of compd **8** (125 MHz, DMSO-*d*6).

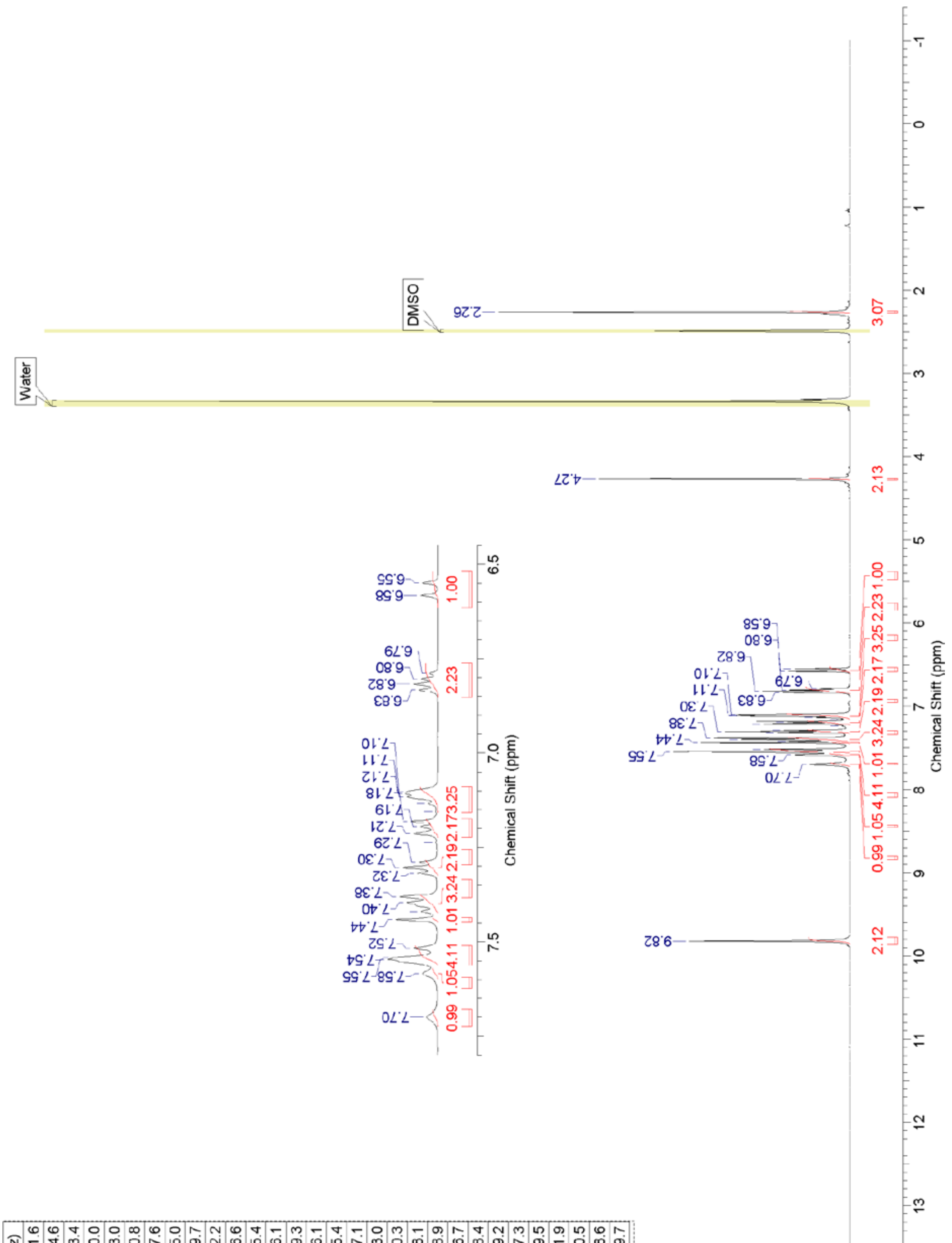

No.	(ppm)	(Hz)
1	2.26	1131.6
2	4.27	2134.6
3	6.55	3273.4
4	6.58	3290.0
5	6.79	3393.0
6	6.80	3400.8
7	6.82	3407.6
8	6.83	3415.0
9	7.10	3549.7
10	7.11	3552.2
11	7.12	3556.6
12	7.13	3565.4
13	7.15	3576.1
14	7.18	3589.3
15	7.19	3596.1
16	7.21	3605.4
17	7.24	3617.1
18	7.29	3643.0
19	7.30	3650.3
20	7.32	3658.1
21	7.38	3688.9
22	7.40	3696.7
23	7.42	3708.4
24	7.44	3719.2
25	7.52	3757.3
26	7.54	3769.5
27	7.55	3771.9
28	7.58	3790.5
29	7.70	3848.6
30	9.82	4909.7

Spectrum 3. ^{1}H NMR of compd **9** (500 MHz, DMSO-d_6).

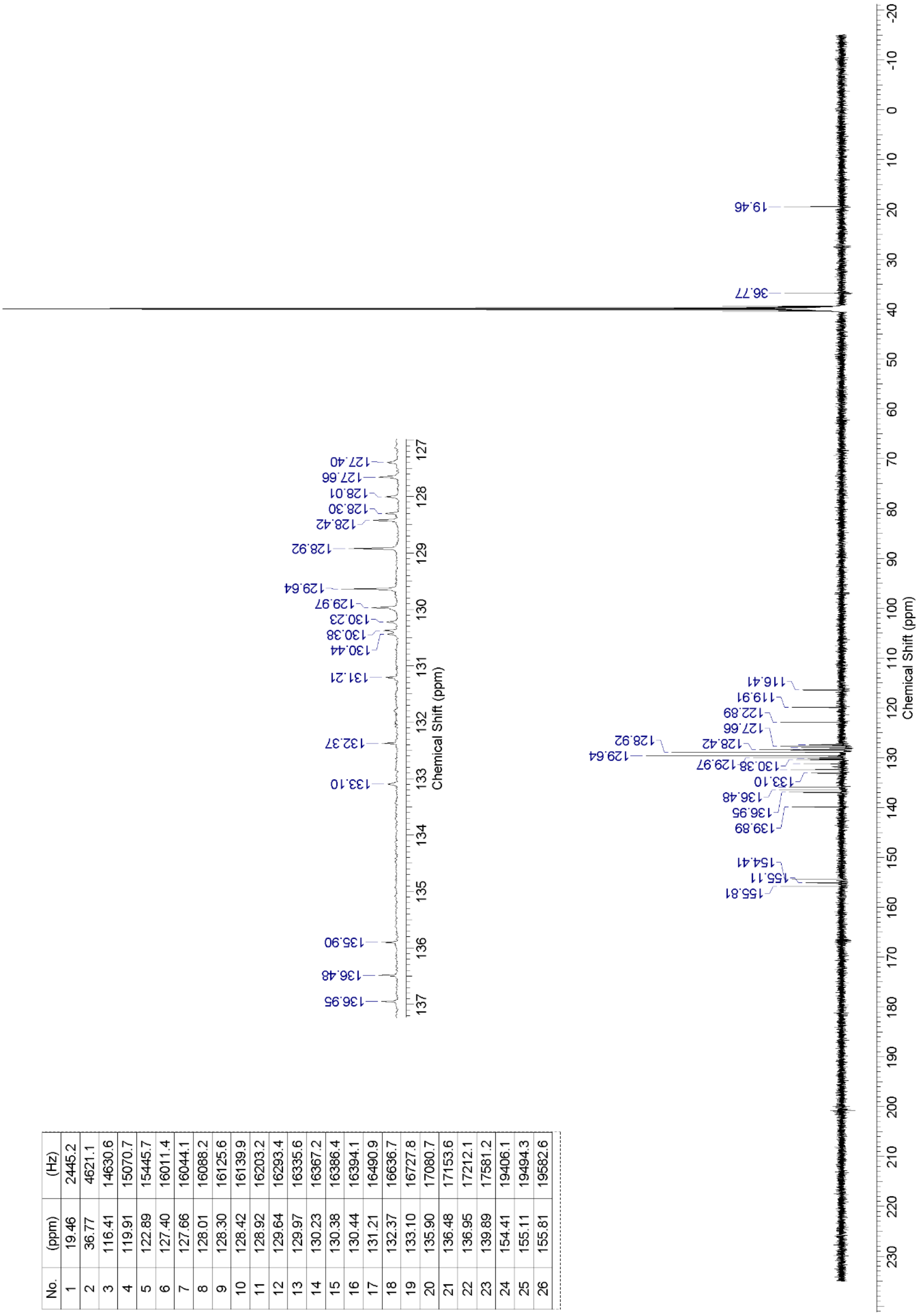

No.	(ppm)	(Hz)
1	19.46	2445.2
2	36.77	4621.1
3	116.41	14630.6
4	119.91	15070.7
5	122.89	15445.7
6	127.40	16011.4
7	127.66	16044.1
8	128.01	16088.2
9	128.30	16125.6
10	128.42	16139.9
11	128.92	16203.2
12	129.64	16293.4
13	129.97	16335.6
14	130.23	16367.2
15	130.38	16386.4
16	130.44	16394.1
17	131.21	16490.9
18	132.37	16636.7
19	133.10	16727.8
20	135.90	17080.7
21	136.48	17153.6
22	136.95	17212.1
23	139.89	17581.2
24	154.41	19406.1
25	155.11	19494.3
26	155.81	19582.6

Spectrum 4. ^{13}C NMR of compd **9** (125 MHz, DMSO-*d*$_6$).

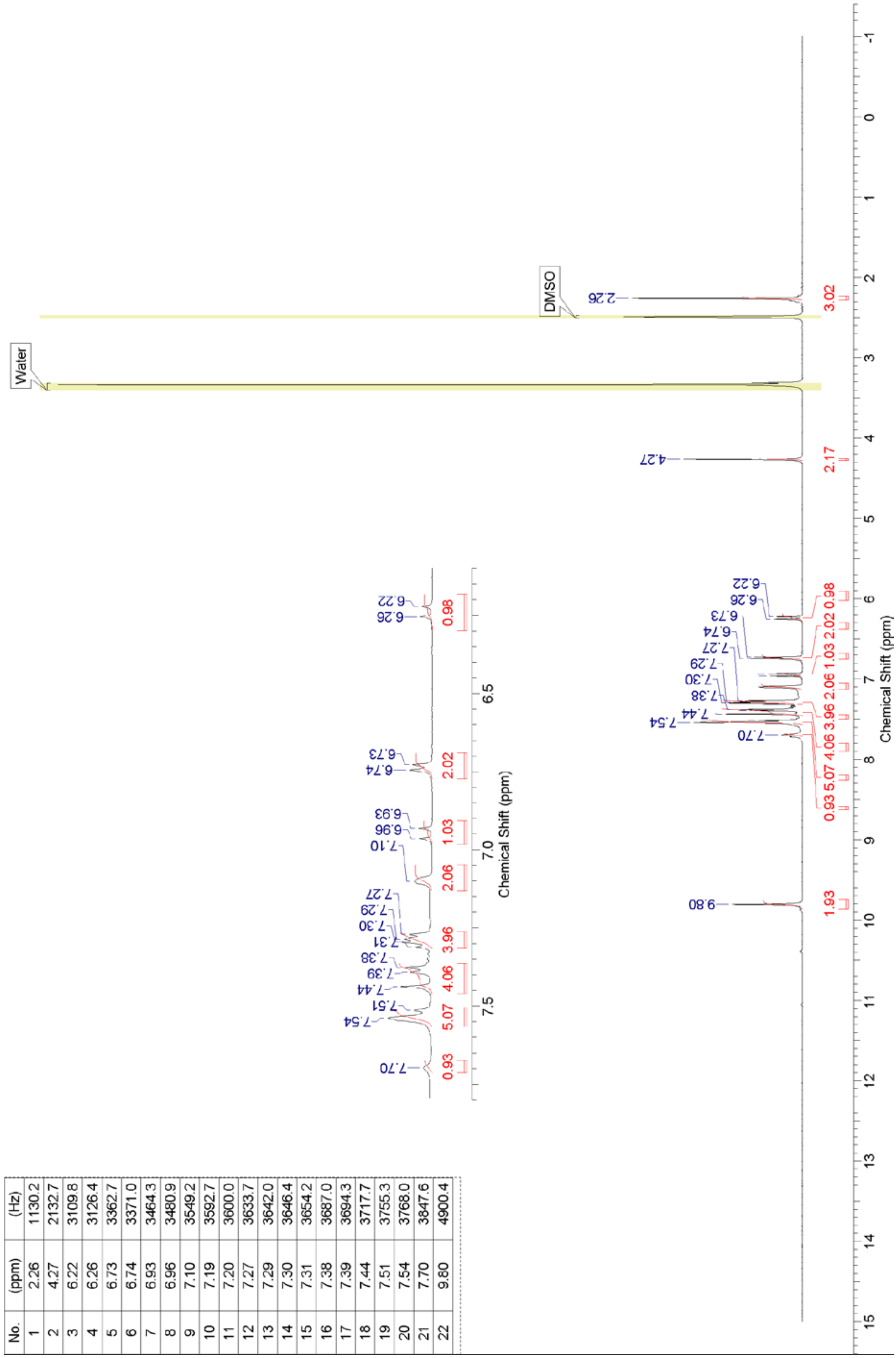

Spectrum 5. ^{1}H NMR of compd **10** (500 MHz, DMSO-d_6).

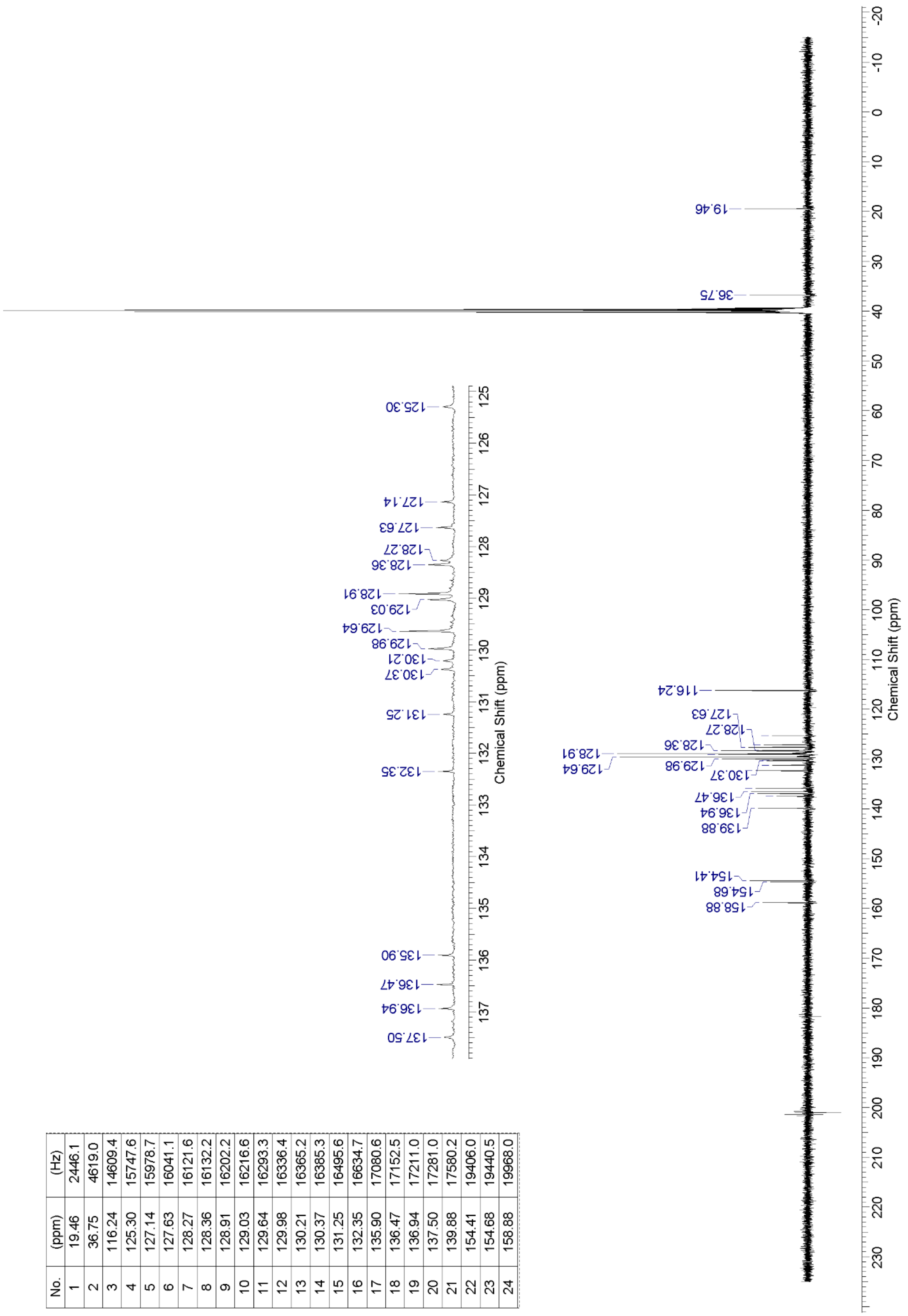

No.	(ppm)	(Hz)
1	19.46	2446.1
2	36.75	4619.0
3	116.24	14609.4
4	125.30	15747.6
5	127.14	15978.7
6	127.63	16041.1
7	128.27	16121.6
8	128.36	16132.2
9	128.91	16202.2
10	129.03	16216.6
11	129.64	16293.3
12	129.98	16336.4
13	130.21	16365.2
14	130.37	16385.3
15	131.25	16495.6
16	132.35	16634.7
17	135.90	17080.6
18	136.47	17152.5
19	136.94	17211.0
20	137.50	17281.0
21	139.88	17580.2
22	154.41	19406.0
23	154.68	19440.5
24	158.88	19968.0

Spectrum 6. ^{13}C NMR of compd **10** (125 MHz, DMSO-d_6).

(ppm)	(Hz)
2.29	458.3
3.78	756.3
4.30	859.3
6.29	1256.8
6.37	1273.2
6.93	1385.5
6.97	13694.1
7.01	1401.5
7.09	1417.9
7.13	1425.0
7.14	1428.3
7.21	1441.3
7.24	1448.3
7.29	1457.4
7.33	1464.8
7.36	1472.0
7.40	1479.9
7.41	1481.7
7.43	1486.4
7.46	1491.1
7.47	1493.6
7.42	1483.2
7.56	1511.2
7.74	1548.6
9.87	1973.1

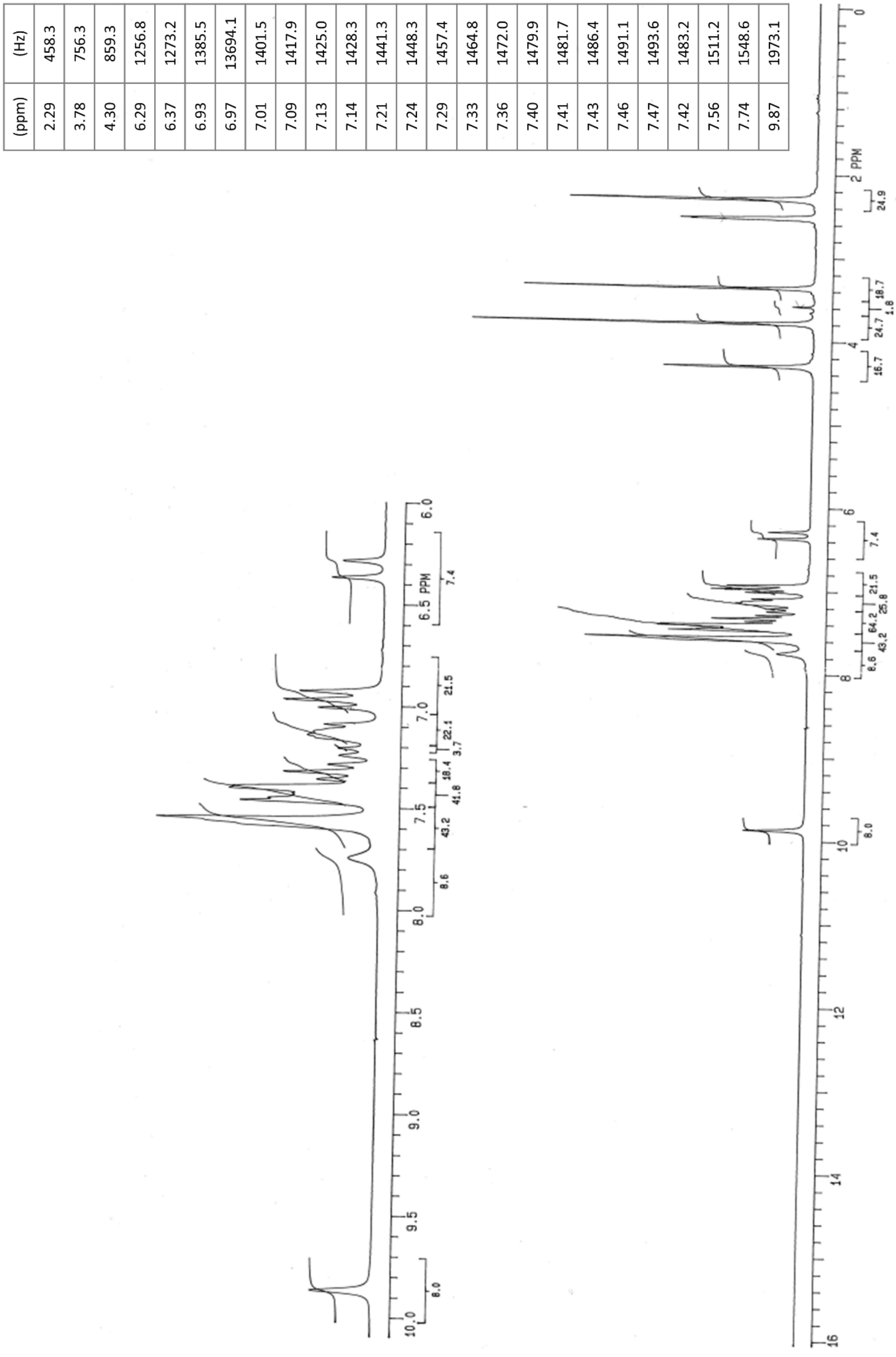

Spectrum 7. ^{1}H NMR of compd **11** (200 MHz, DMSO-d_6).

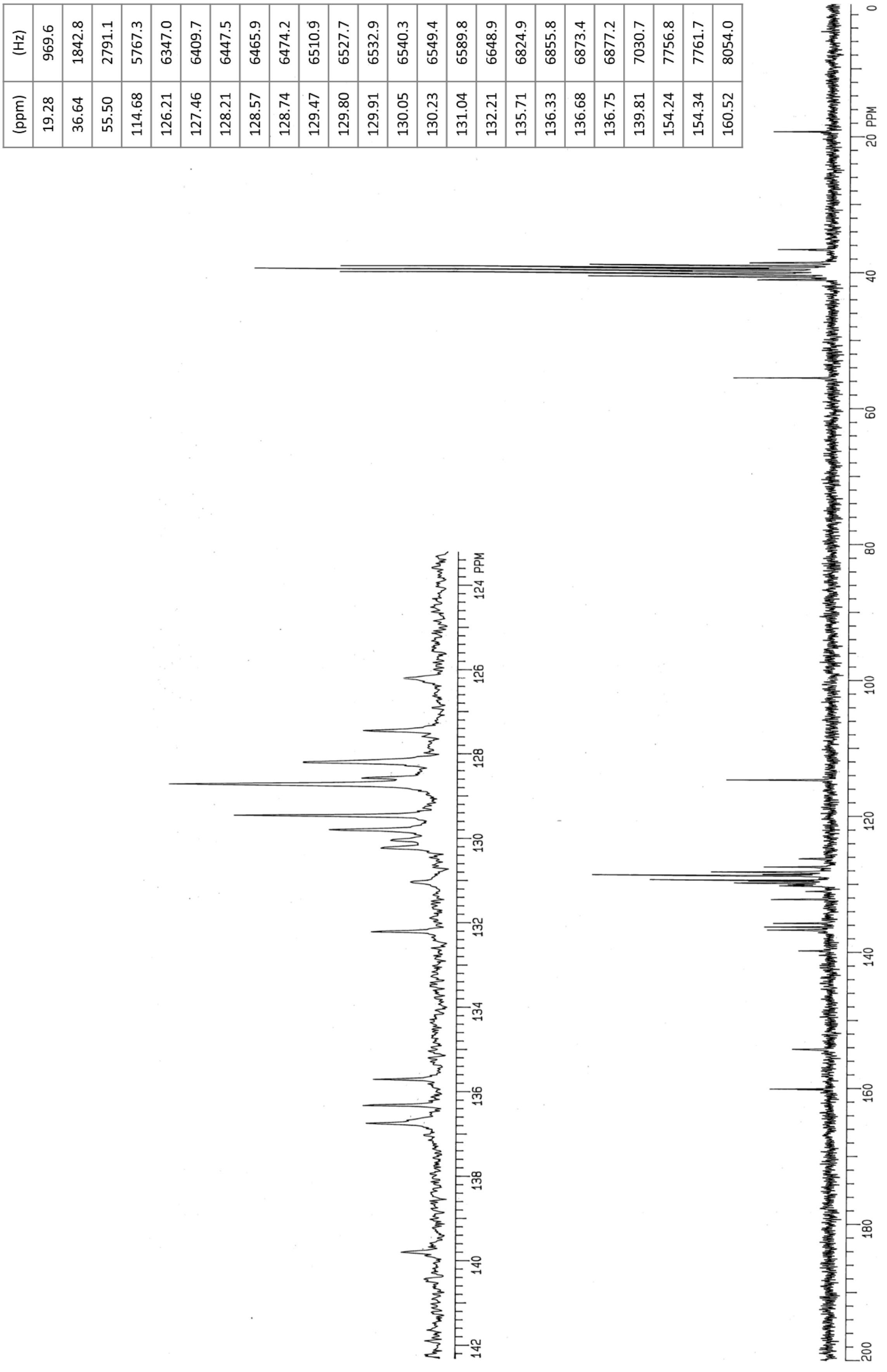

Spectrum 8. ^{13}CNMR of compd **11** (50 MHz, DMSO-d_6).

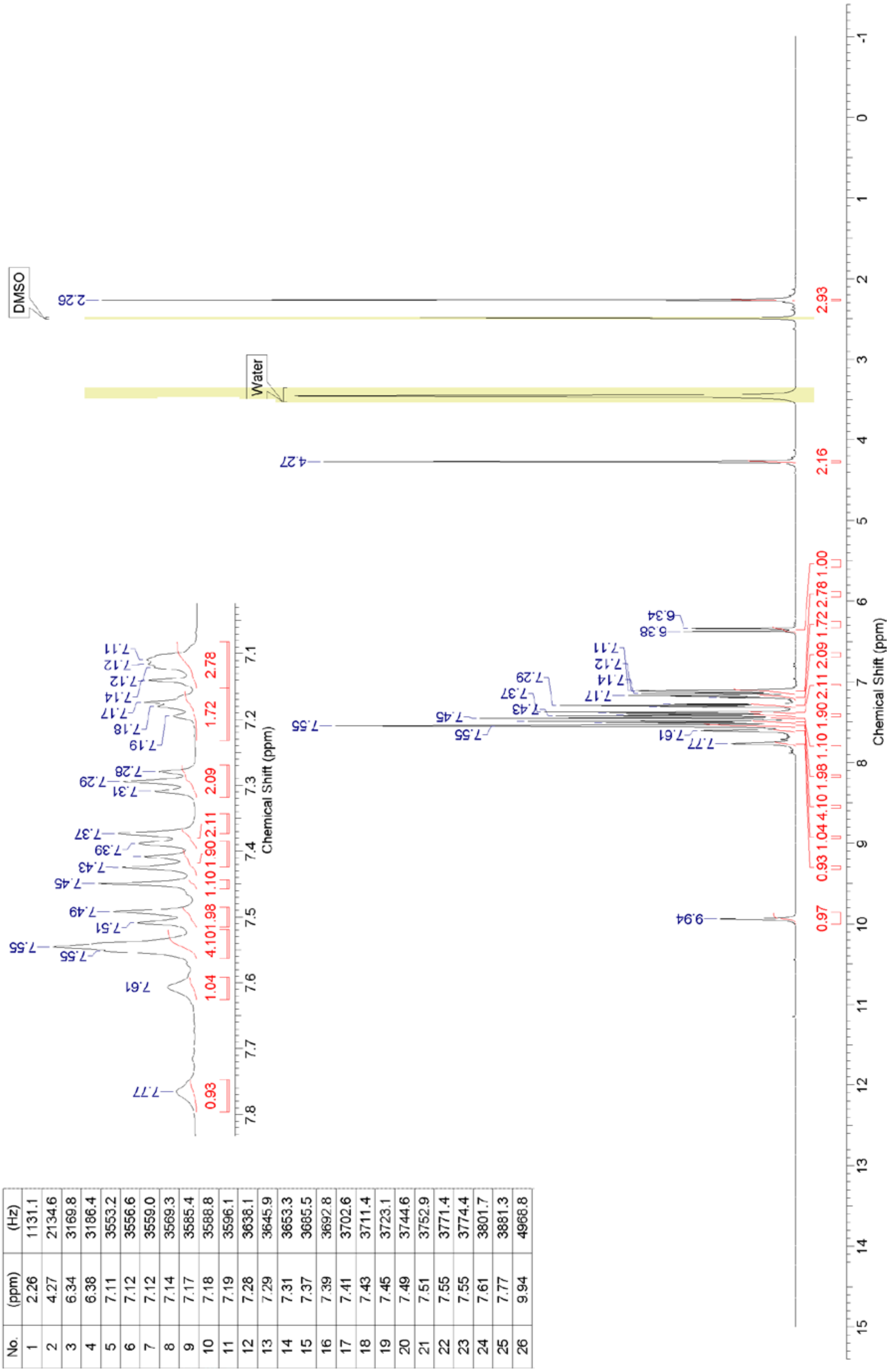

No.	(ppm)	(Hz)
1	2.26	1131.1
2	4.27	2134.6
3	6.34	3169.8
4	6.38	3186.4
5	7.11	3553.2
6	7.12	3556.6
7	7.12	3559.0
8	7.14	3569.3
9	7.17	3585.4
10	7.18	3588.8
11	7.19	3596.1
12	7.28	3638.1
13	7.29	3645.9
14	7.31	3653.3
15	7.37	3685.5
16	7.39	3692.8
17	7.41	3702.6
18	7.43	3711.4
19	7.45	3723.1
20	7.49	3744.6
21	7.51	3752.9
22	7.55	3771.4
23	7.55	3774.4
24	7.61	3801.7
25	7.77	3881.3
26	9.94	4968.8

Spectrum 9. ^{1}H NMR of compd **12** (500 MHz, DMSO-d_6).

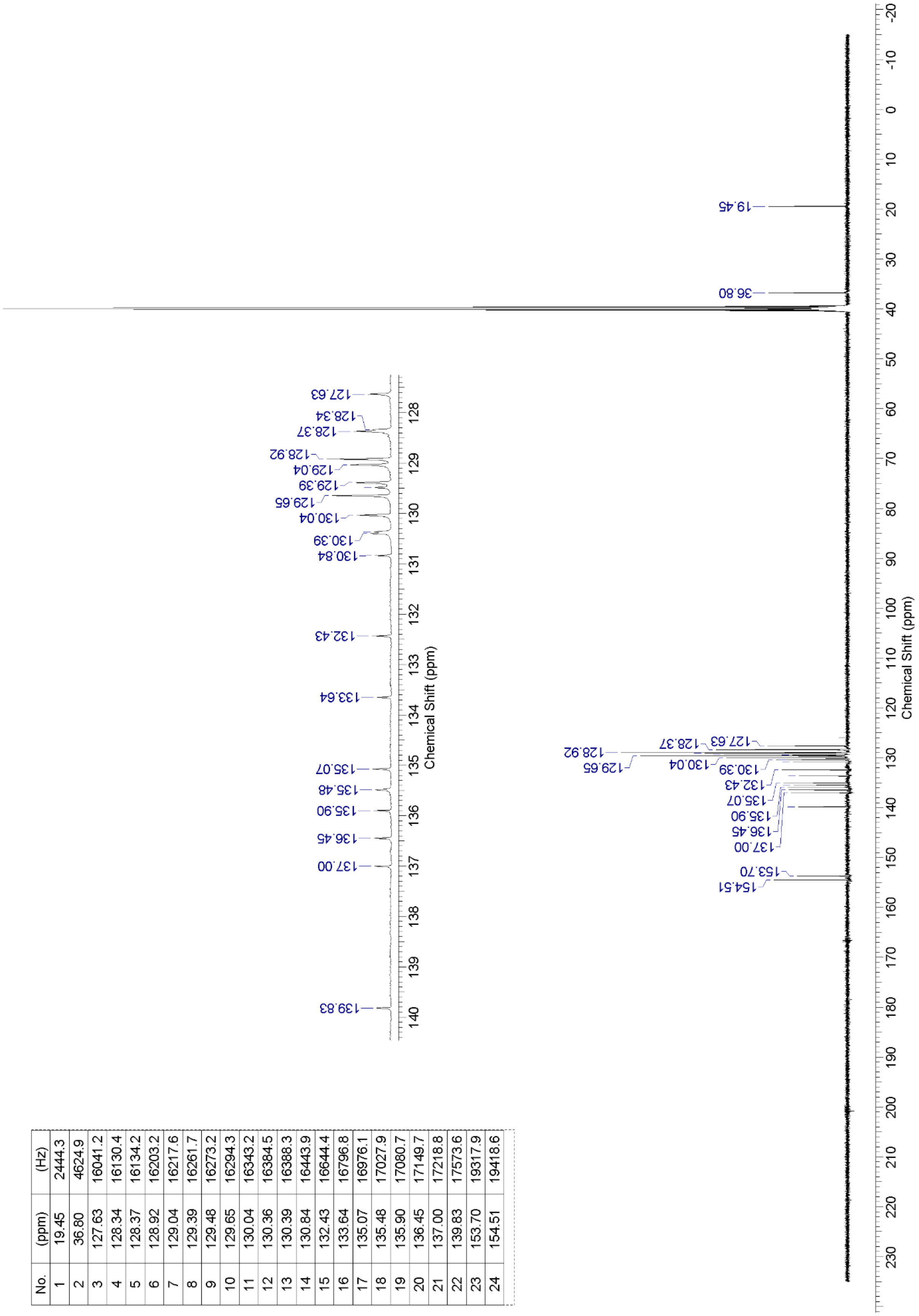

No.	(ppm)	(Hz)
1	19.45	2444.3
2	36.80	4624.9
3	127.63	16041.2
4	128.34	16130.4
5	128.37	16134.2
6	128.92	16203.2
7	129.04	16217.6
8	129.39	16261.7
9	129.48	16273.2
10	129.65	16294.3
11	130.04	16343.2
12	130.36	16384.5
13	130.39	16388.3
14	130.84	16443.9
15	132.43	16644.4
16	133.64	16796.8
17	135.07	16976.1
18	135.48	17027.9
19	135.90	17080.7
20	136.45	17149.7
21	137.00	17218.8
22	139.83	17573.6
23	153.70	19317.9
24	154.51	19418.6

Spectrum 10. ^{13}C NMR of compd **12** (125 MHz, DMSO-*d*$_6$).

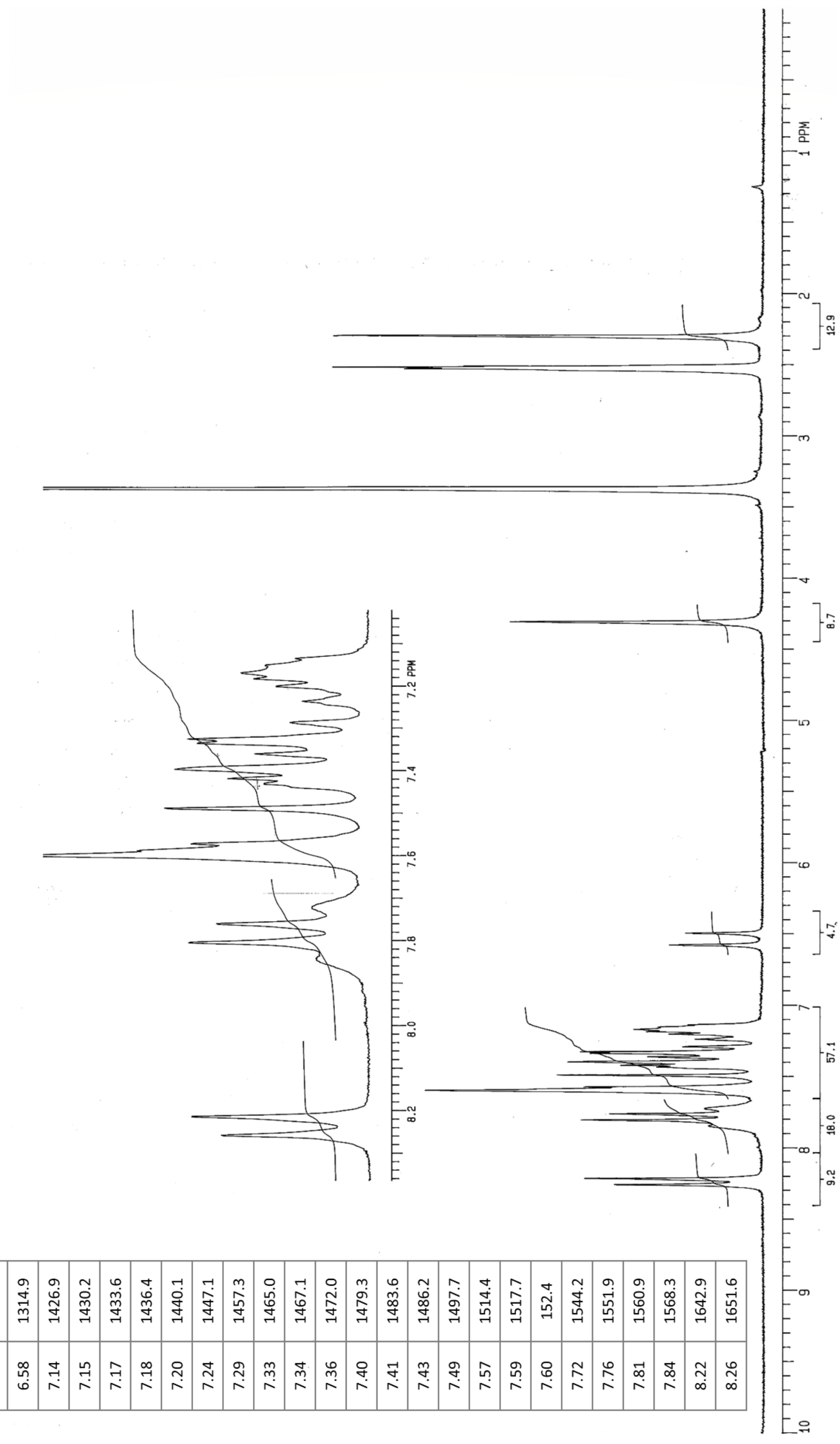

(ppm)	(Hz)
2.30	459.6
4.31	861.0
6.50	1298.7
6.58	1314.9
7.14	1426.9
7.15	1430.2
7.17	1433.6
7.18	1436.4
7.20	1440.1
7.24	1447.1
7.29	1457.3
7.33	1465.0
7.34	1467.1
7.36	1472.0
7.40	1479.3
7.41	1483.6
7.43	1486.2
7.49	1497.7
7.57	1514.4
7.59	1517.7
7.60	152.4
7.72	1544.2
7.76	1551.9
7.81	1560.9
7.84	1568.3
8.22	1642.9
8.26	1651.6

Spectrum 11. ^{1}H NMR of compd **13** (200 MHz, DMSO-d_6).

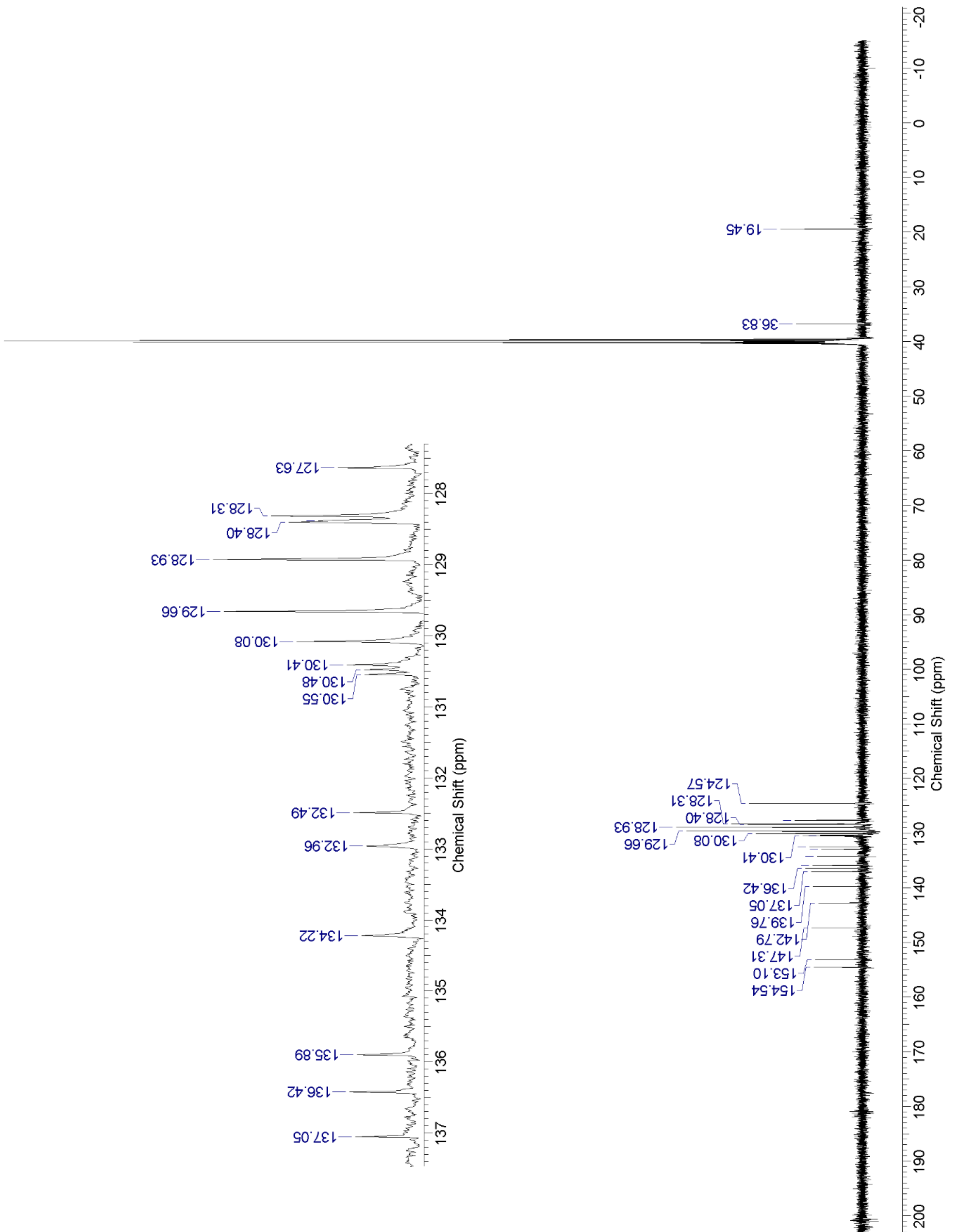

No.	(ppm)	(Hz)
1	19.45	2444.2
2	36.83	4628.6
3	124.57	15656.5
4	127.63	16041.1
5	128.31	16126.4
6	128.38	16135.1
7	128.40	16137.9
8	128.93	16204.1
9	129.66	16296.2
10	130.08	16348.9
11	130.41	16390.1
12	130.48	16398.8
13	130.55	16407.4
14	132.49	16651.9
15	132.96	16710.4
16	134.22	16869.6
17	135.89	17079.6
18	136.42	17145.8
19	137.05	17225.4
20	139.76	17565.8
21	142.79	17946.5
22	147.31	18514.2
23	153.10	19242.0
24	154.54	19423.3

Spectrum 12. ^{13}C NMR of compd **13** (125 MHz, DMSO-d_6).

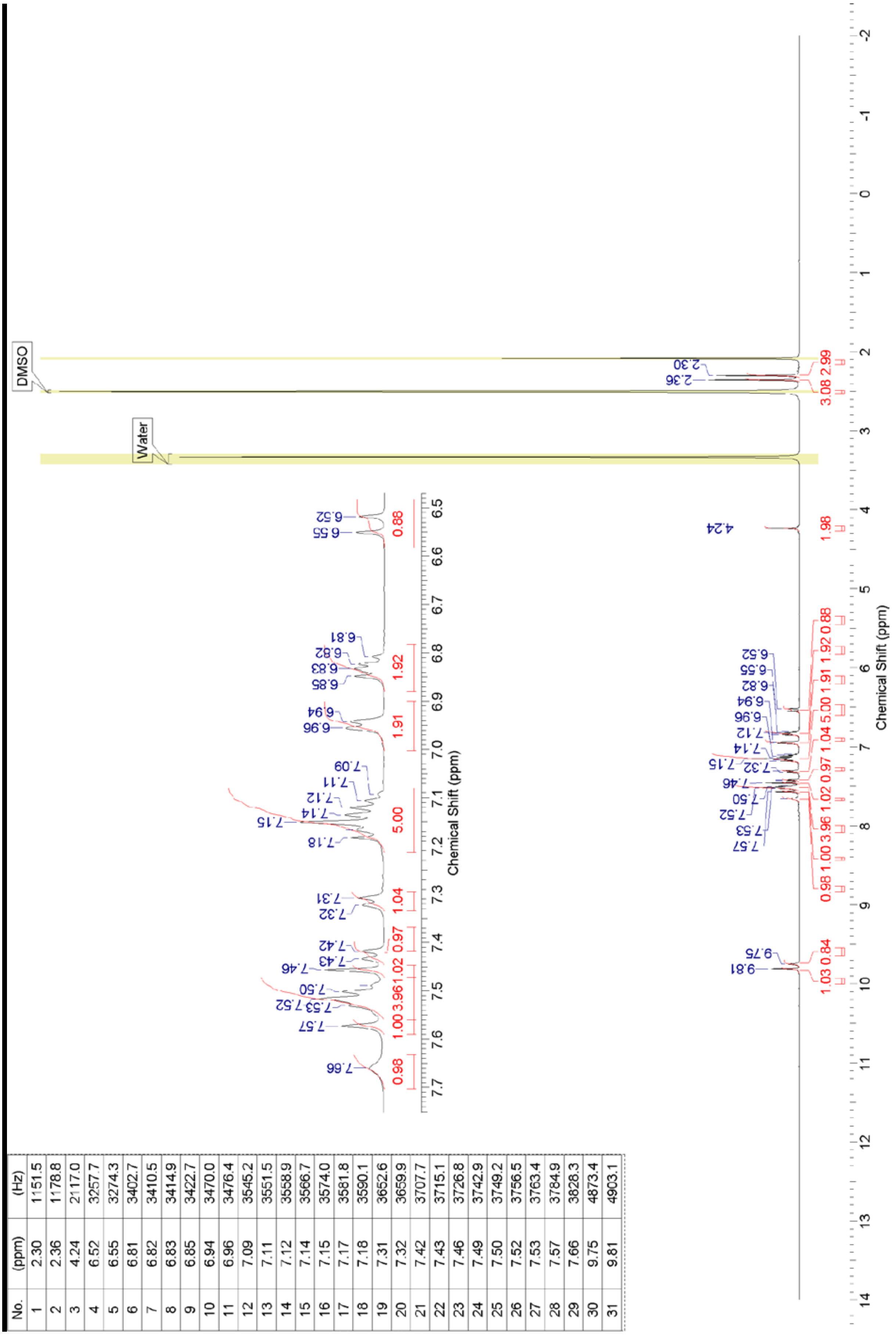

No.	(ppm)	(Hz)
1	2.30	1151.5
2	2.36	1178.8
3	4.24	2117.0
4	6.52	3257.7
5	6.55	3274.3
6	6.81	3402.7
7	6.82	3410.5
8	6.83	3414.9
9	6.85	3422.7
10	6.94	3470.0
11	6.96	3476.4
12	7.09	3545.2
13	7.11	3551.5
14	7.12	3558.9
15	7.14	3566.7
16	7.15	3574.0
17	7.17	3581.8
18	7.18	3590.1
19	7.31	3652.6
20	7.32	3659.9
21	7.42	3707.7
22	7.43	3715.1
23	7.46	3726.8
24	7.49	3742.9
25	7.50	3749.2
26	7.52	3756.5
27	7.53	3763.4
28	7.57	3784.9
29	7.66	3828.3
30	9.75	4873.4
31	9.81	4903.1

Spectrum 13. ^{1}H NMR of compd **14** (500 MHz, DMSO-d_6).

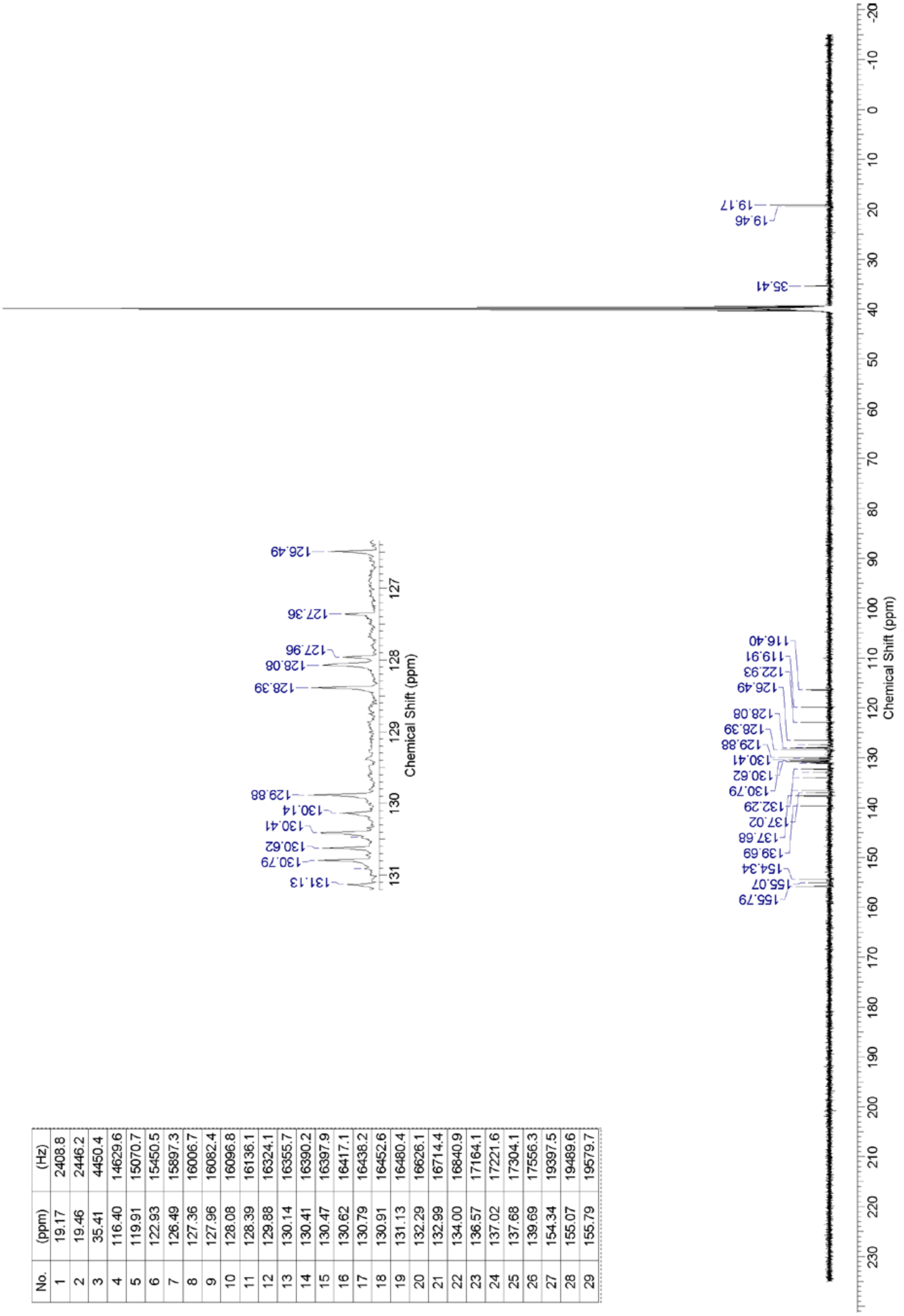

No.	(ppm)	(Hz)
1	19.17	2408.8
2	19.46	2446.2
3	35.41	4450.4
4	116.40	14629.6
5	119.91	15070.7
6	122.93	15450.5
7	126.49	15897.3
8	127.36	16006.7
9	127.96	16082.4
10	128.08	16096.8
11	128.39	16136.1
12	129.88	16324.1
13	130.14	16355.7
14	130.41	16390.2
15	130.47	16397.9
16	130.62	16417.1
17	130.79	16438.2
18	130.91	16452.6
19	131.13	16480.4
20	132.29	16626.1
21	132.99	16714.4
22	134.00	16840.9
23	136.57	17164.1
24	137.02	17221.6
25	137.68	17304.1
26	139.69	17556.3
27	154.34	19397.5
28	155.07	19489.6
29	155.79	19579.7

Spectrum 14. ^{13}C NMR of compd **14** (125 MHz, DMSO-d_6).

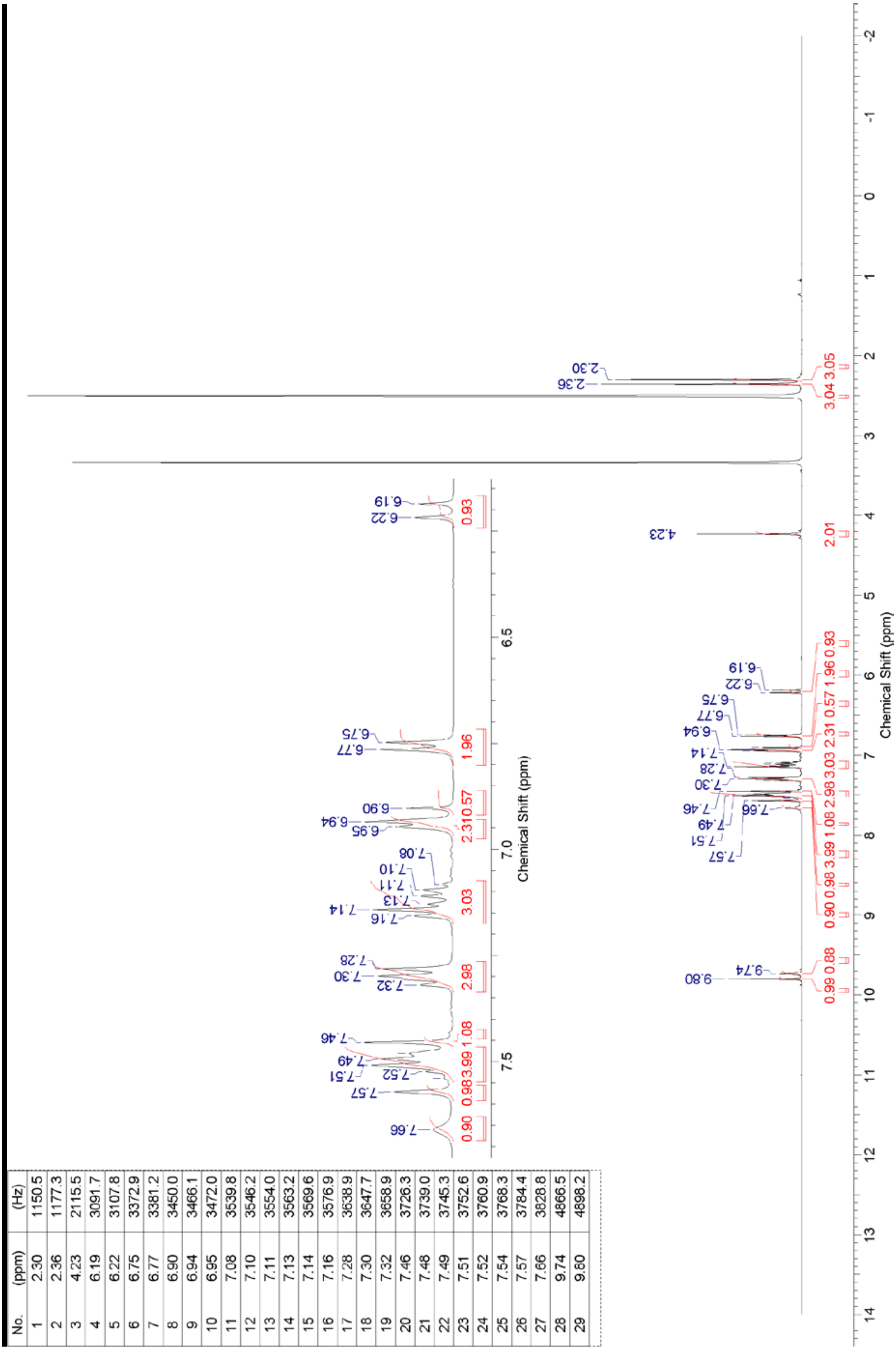

Spectrum 15. ^{1}H NMR of compd **15** (500 MHz, DMSO-d_6).

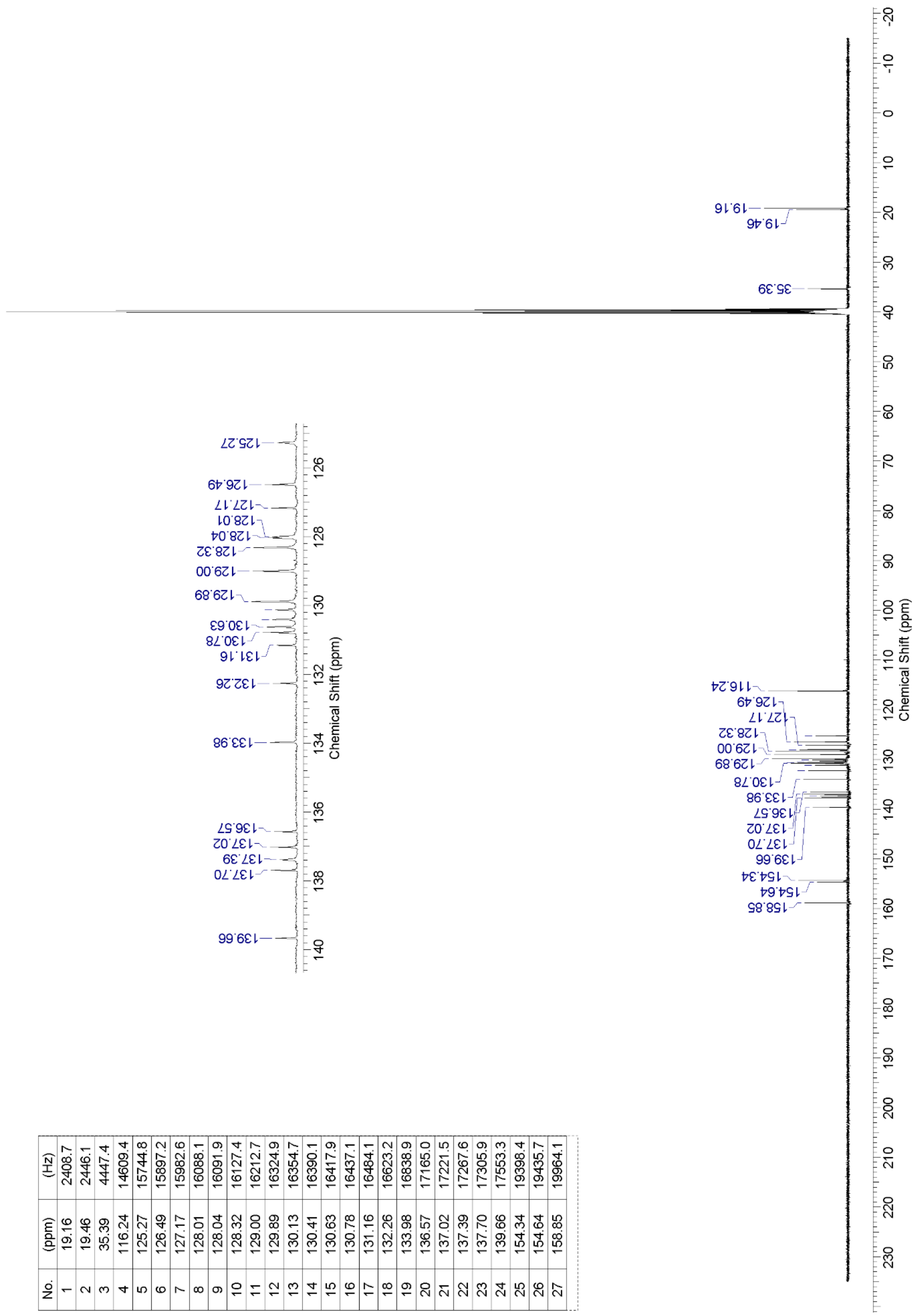

No.	(ppm)	(Hz)
1	19.16	2408.7
2	19.46	2446.1
3	35.39	4447.4
4	116.24	14609.4
5	125.27	15744.8
6	126.49	15897.2
7	127.17	15982.6
8	128.01	16088.1
9	128.04	16091.9
10	128.32	16127.4
11	129.00	16212.7
12	129.89	16324.9
13	130.13	16354.7
14	130.41	16390.1
15	130.63	16417.9
16	130.78	16437.1
17	131.16	16484.1
18	132.26	16623.2
19	133.98	16838.9
20	136.57	17165.0
21	137.02	17221.5
22	137.39	17267.6
23	137.70	17305.9
24	139.66	17553.3
25	154.34	19398.4
26	154.64	19435.7
27	158.85	19964.1

Spectrum 16. ^{13}C NMR of compd **15** (125 MHz, DMSO-*d*$_6$).

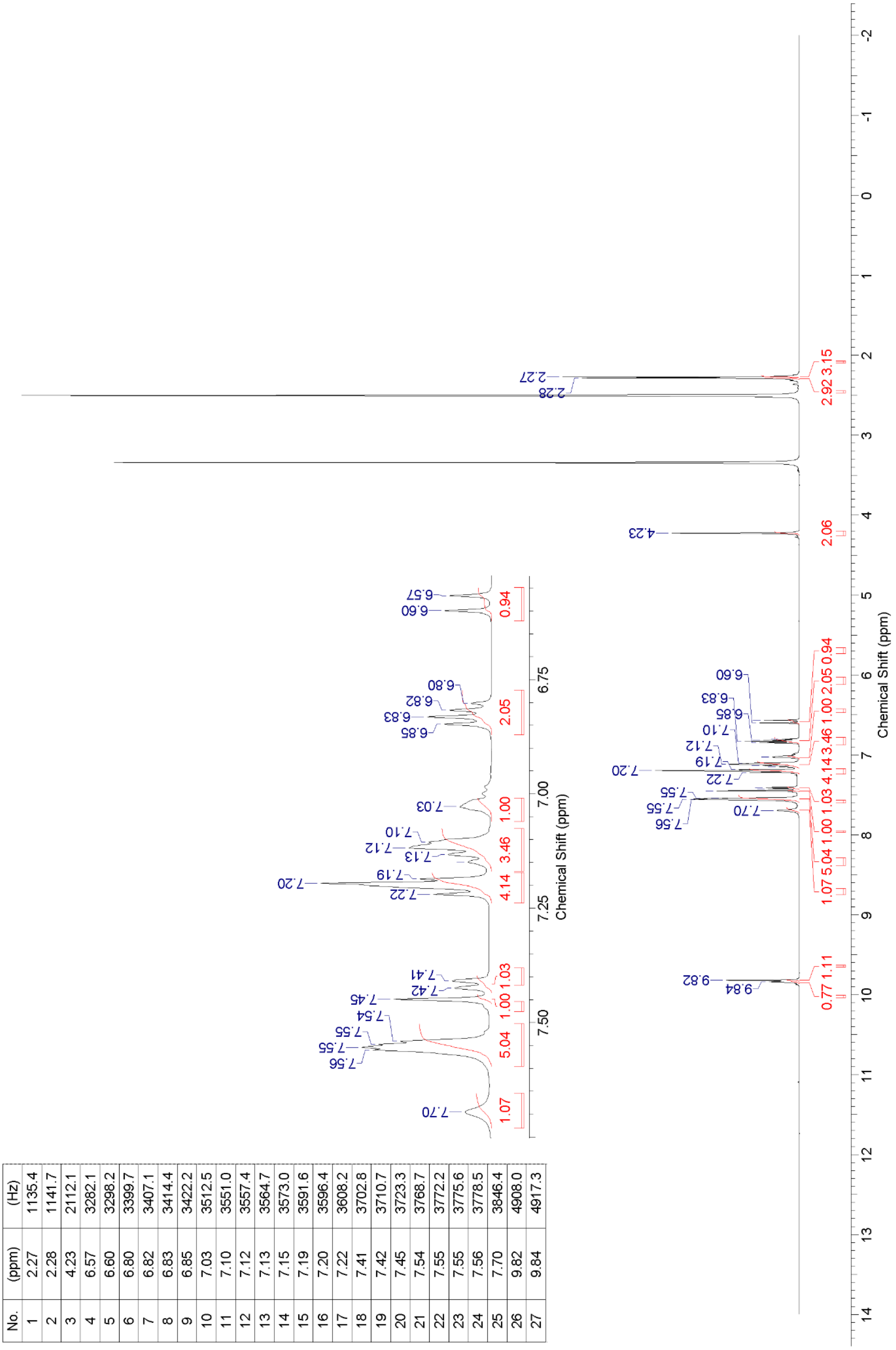

No.	(ppm)	(Hz)
1	2.27	1135.4
2	2.28	1141.7
3	4.23	2112.1
4	6.57	3282.1
5	6.60	3298.2
6	6.80	3399.7
7	6.82	3407.1
8	6.83	3414.4
9	6.85	3422.2
10	7.03	3512.5
11	7.10	3551.0
12	7.12	3557.4
13	7.13	3564.7
14	7.15	3573.0
15	7.19	3591.6
16	7.20	3596.4
17	7.22	3608.2
18	7.41	3702.8
19	7.42	3710.7
20	7.45	3723.3
21	7.54	3768.7
22	7.55	3772.2
23	7.55	3775.6
24	7.56	3778.5
25	7.70	3846.4
26	9.82	4908.0
27	9.84	4917.3

Spectrum 17. ^{1}H NMR of compd **16** (500 MHz, DMSO-d_6).

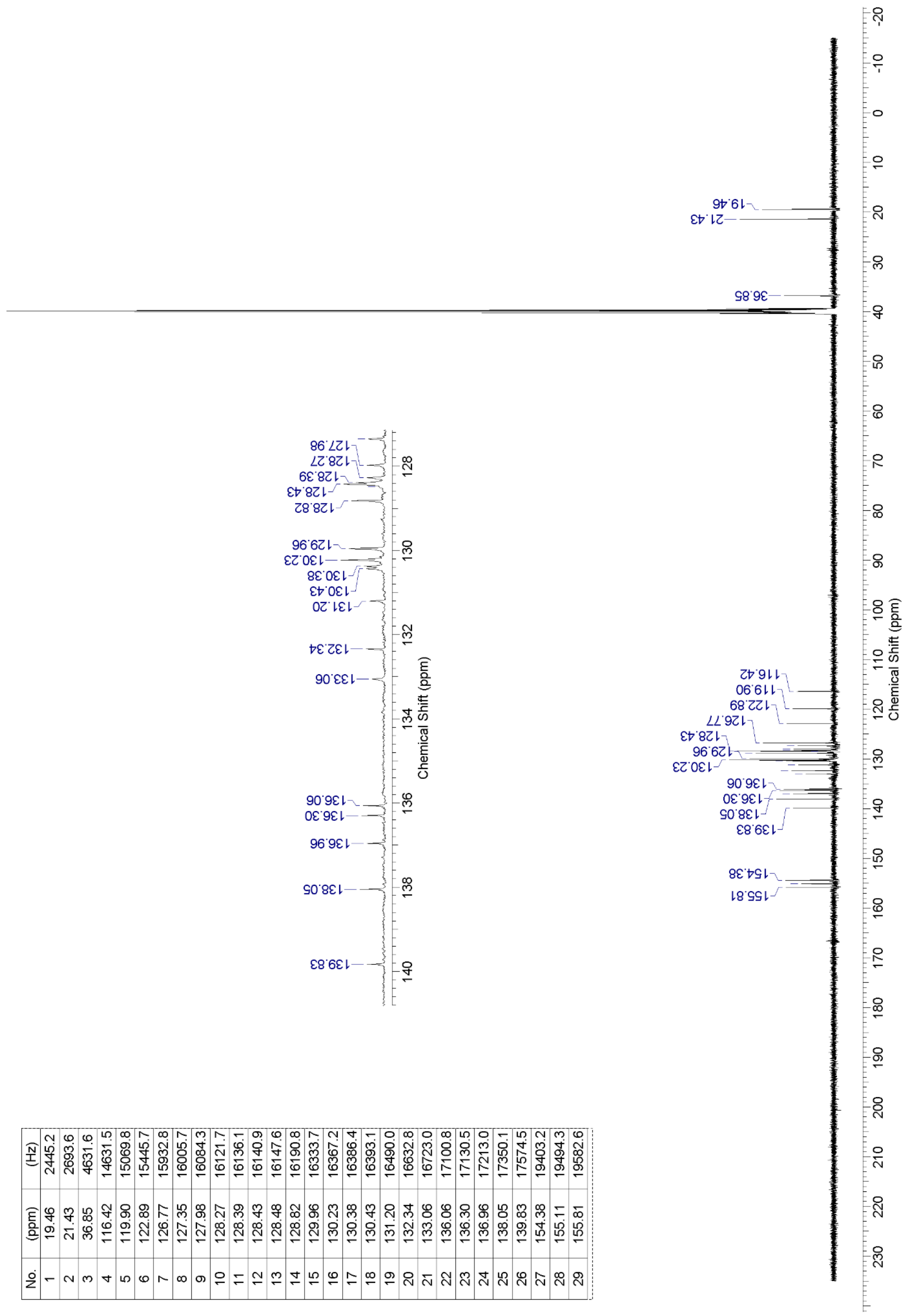

No.	(ppm)	(Hz)
1	19.46	2445.2
2	21.43	2693.6
3	36.85	4631.6
4	116.42	14631.5
5	119.90	15069.8
6	122.89	15445.7
7	126.77	15932.8
8	127.35	16005.7
9	127.98	16084.3
10	128.27	16121.7
11	128.39	16136.1
12	128.43	16140.9
13	128.48	16147.6
14	128.82	16190.8
15	129.96	16333.7
16	130.23	16367.2
17	130.38	16386.4
18	130.43	16393.1
19	131.20	16490.0
20	132.34	16632.8
21	133.06	16723.0
22	136.06	17100.8
23	136.30	17130.5
24	136.96	17213.0
25	138.05	17350.1
26	139.83	17574.5
27	154.38	19403.2
28	155.11	19494.3
29	155.81	19582.6

Spectrum 18. ^{13}C NMR of compd **16** (125 MHz, DMSO-d_6).

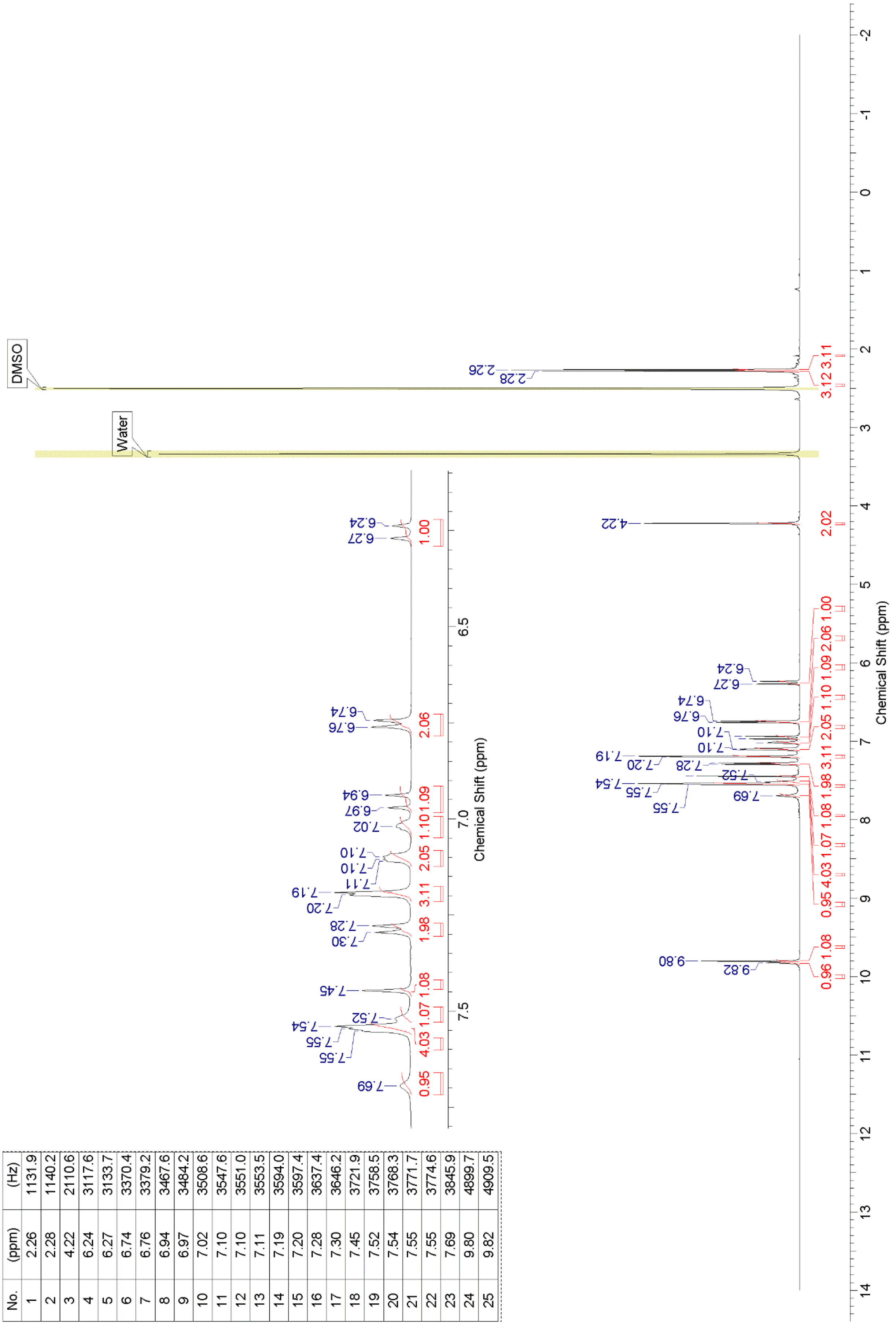

No.	(ppm)	(Hz)
1	2.26	1131.9
2	2.28	1140.2
3	4.22	2110.6
4	6.24	3117.6
5	6.27	3133.7
6	6.74	3370.4
7	6.76	3379.2
8	6.94	3467.6
9	6.97	3484.2
10	7.02	3508.6
11	7.10	3547.6
12	7.10	3551.0
13	7.11	3553.5
14	7.19	3594.0
15	7.20	3597.4
16	7.28	3637.4
17	7.30	3646.2
18	7.45	3721.9
19	7.52	3758.5
20	7.54	3768.3
21	7.55	3771.7
22	7.55	3774.6
23	7.69	3845.9
24	9.80	4899.7
25	9.82	4909.5

Spectrum 19. ^{1}H NMR of compd **17** (500 MHz, DMSO-d_6).

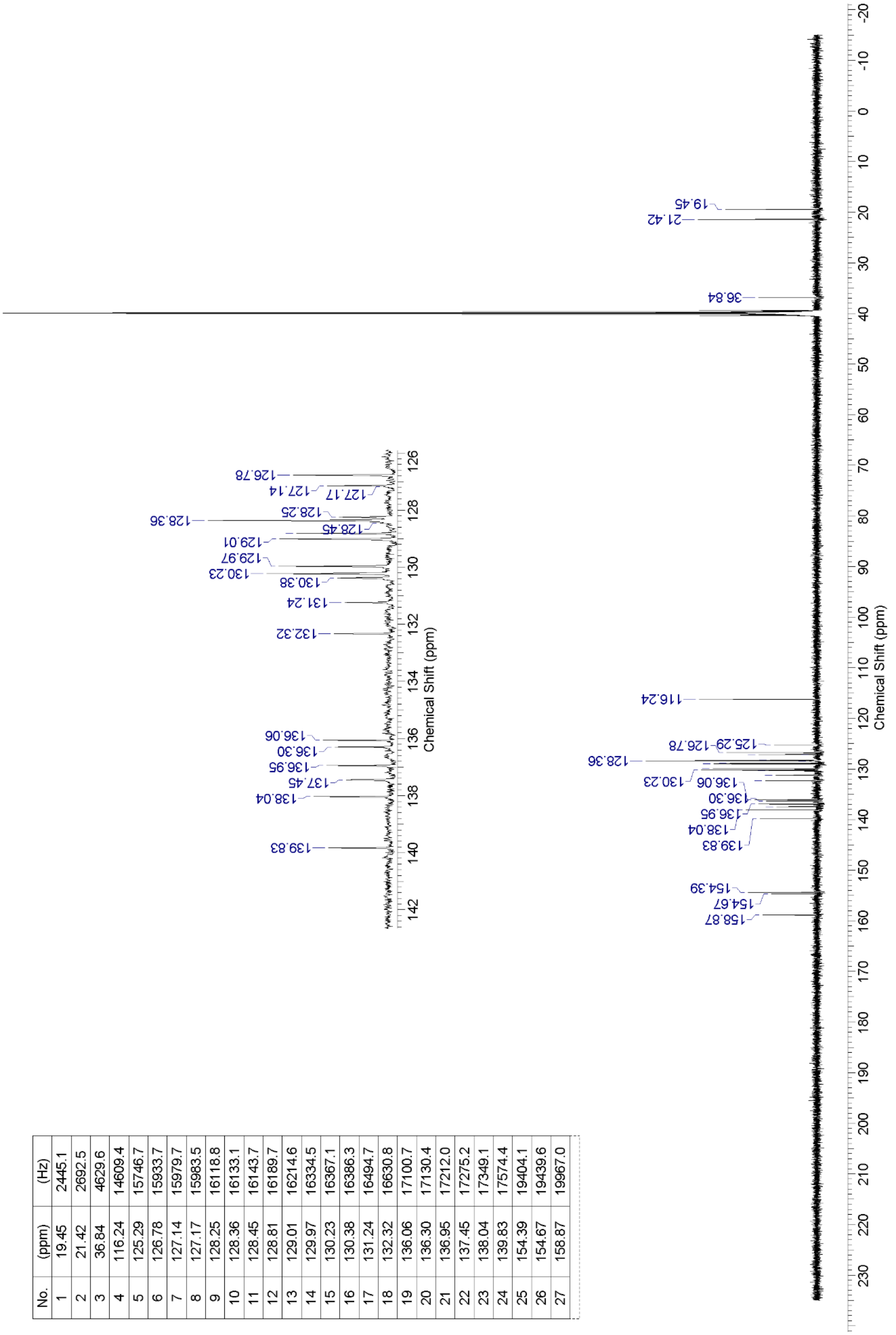

No.	(ppm)	(Hz)
1	19.45	2445.1
2	21.42	2692.5
3	36.84	4629.6
4	116.24	14609.4
5	125.29	15746.7
6	126.78	15933.7
7	127.14	15979.7
8	127.17	15983.5
9	128.25	16118.8
10	128.36	16133.1
11	128.45	16143.7
12	128.81	16189.7
13	129.01	16214.6
14	129.97	16334.5
15	130.23	16367.1
16	130.38	16386.3
17	131.24	16494.7
18	132.32	16630.8
19	136.06	17100.7
20	136.30	17130.4
21	136.95	17212.0
22	137.45	17275.2
23	138.04	17349.1
24	139.83	17574.4
25	154.39	19404.1
26	154.67	19439.6
27	158.87	19967.0

Spectrum 20. ^{13}C NMR of compd **17** (125 MHz, DMSO-d_6).

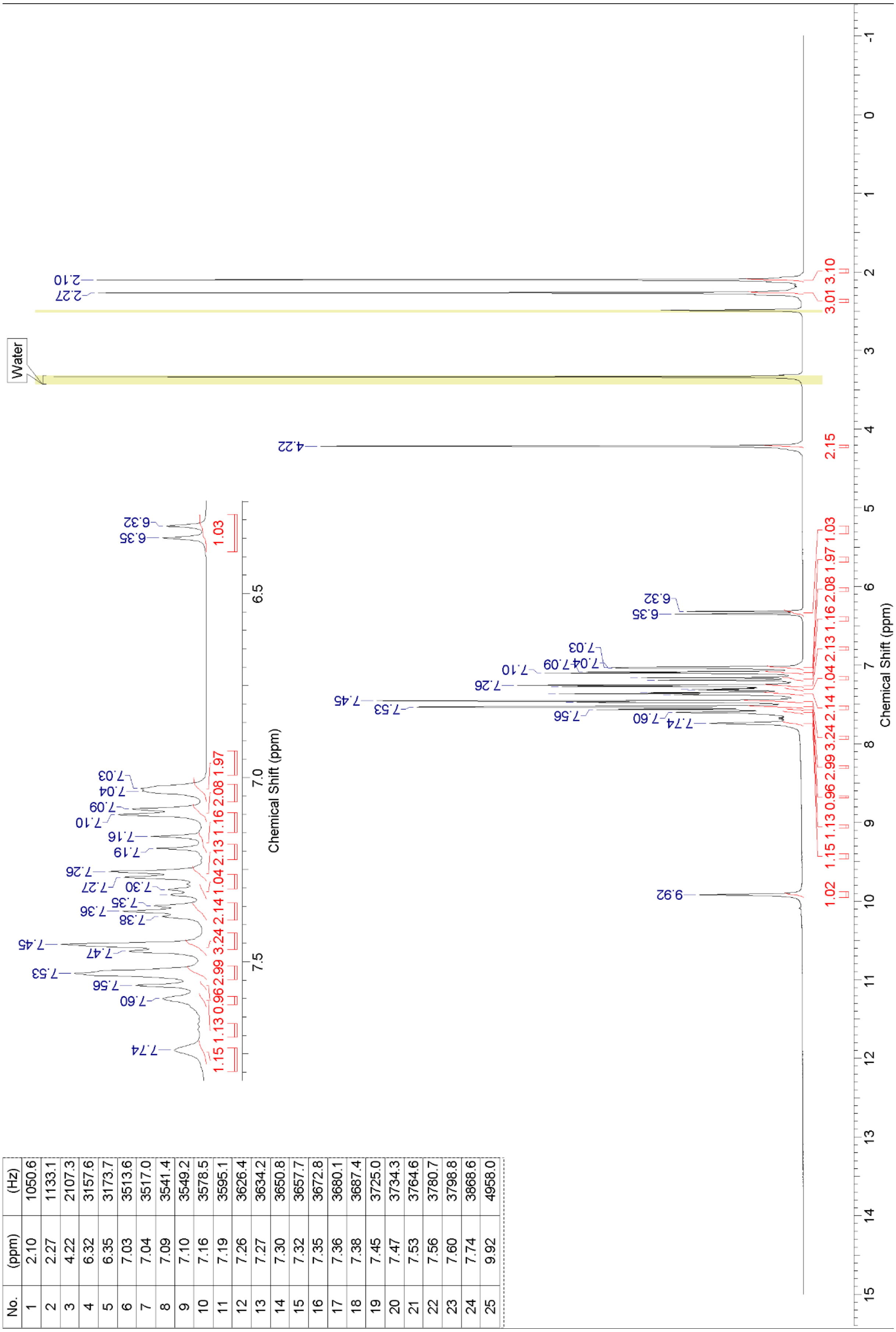

No.	(ppm)	(Hz)
1	2.10	1050.6
2	2.27	1133.1
3	4.22	2107.3
4	6.32	3157.6
5	6.35	3173.7
6	7.03	3513.6
7	7.04	3517.0
8	7.09	3541.4
9	7.10	3549.2
10	7.16	3578.5
11	7.19	3595.1
12	7.26	3626.4
13	7.27	3634.2
14	7.30	3650.8
15	7.32	3657.7
16	7.35	3672.8
17	7.36	3680.1
18	7.38	3687.4
19	7.45	3725.0
20	7.47	3734.3
21	7.53	3764.6
22	7.56	3780.7
23	7.60	3798.8
24	7.74	3868.6
25	9.92	4958.0

Spectrum 21. ^{1}H NMR of compd **18** (500 MHz, DMSO-d_6).

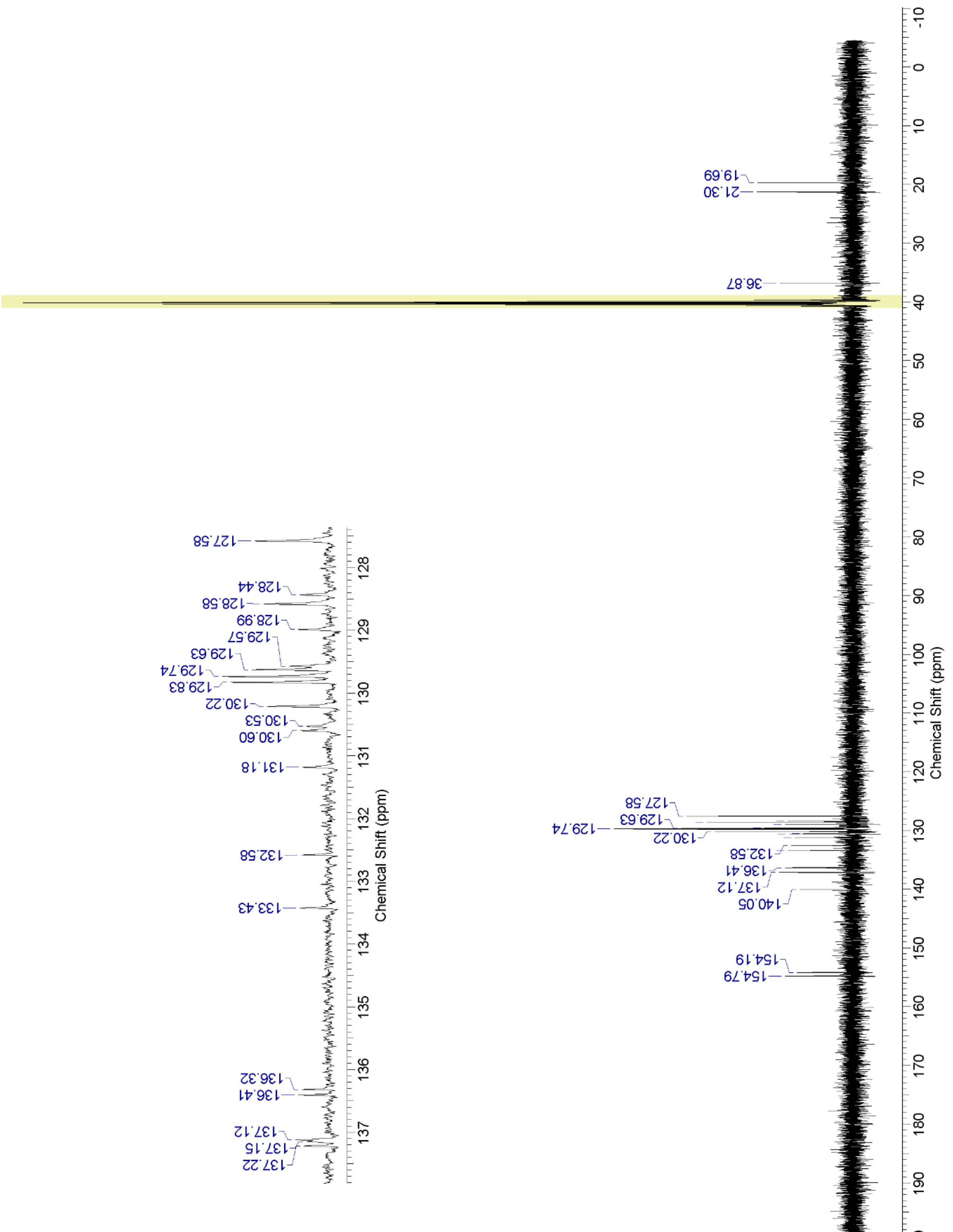

No.	(ppm)	(Hz)
1	19.69	2474.5
2	21.30	2677.3
3	36.87	4634.9
4	127.58	16035.4
5	128.44	16143.5
6	128.58	16161.9
7	128.99	16212.4
8	129.57	16286.2
9	129.63	16293.2
10	129.74	16306.8
11	129.83	16318.2
12	130.22	16367.0
13	130.53	16406.5
14	130.60	16415.3
15	131.18	16488.6
16	132.58	16664.2
17	133.43	16770.5
18	136.32	17134.1
19	136.41	17145.1
20	137.12	17235.1
21	137.15	17238.6
22	137.22	17246.9
23	140.05	17603.1
24	154.19	19380.1
25	154.79	19455.7

Spectrum 22. ^{13}C NMR of compd **18** (125 MHz, DMSO-d_6).

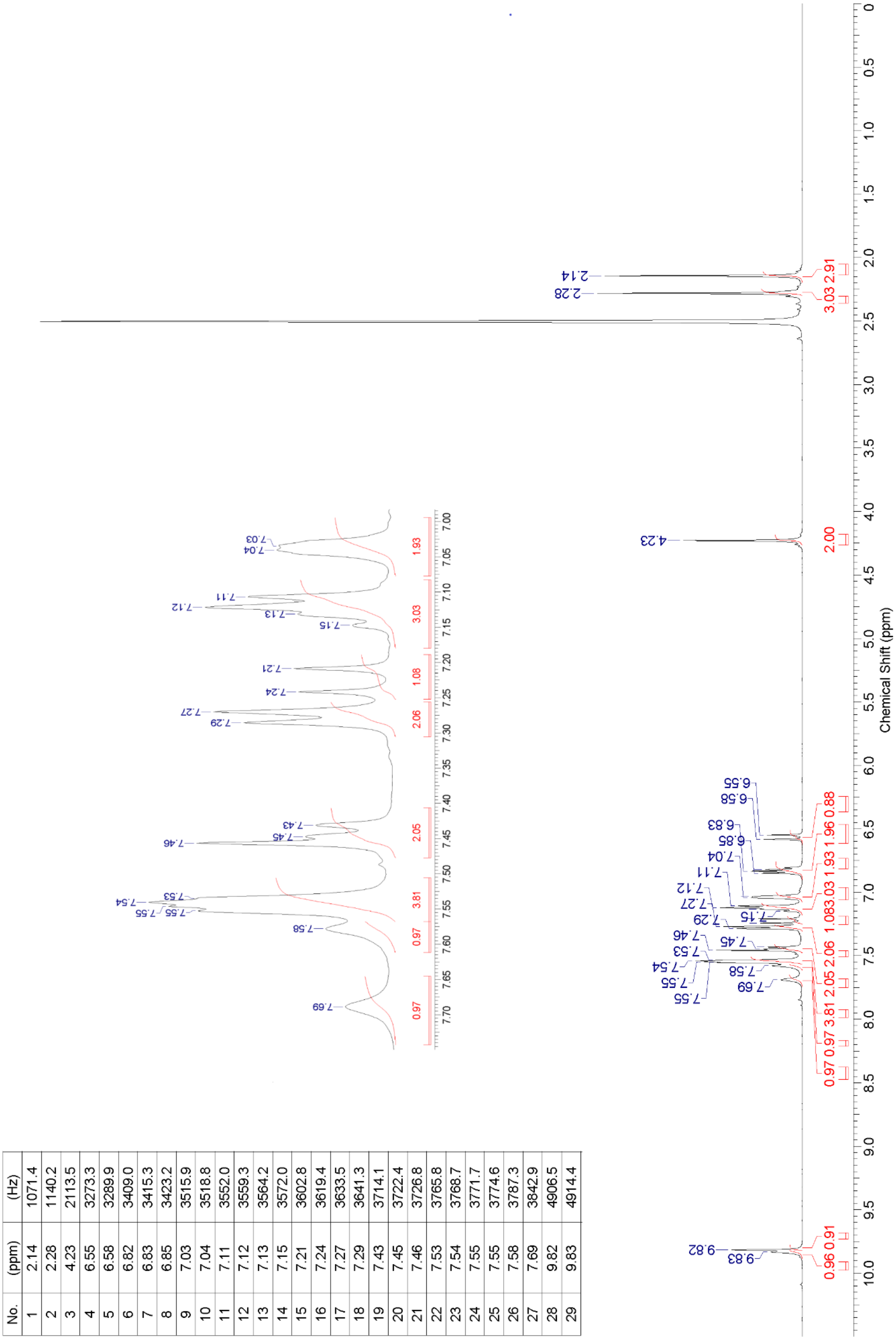

No.	(ppm)	(Hz)
1	2.14	1071.4
2	2.28	1140.2
3	4.23	2113.5
4	6.55	3273.3
5	6.58	3289.9
6	6.82	3409.0
7	6.83	3415.3
8	6.85	3423.2
9	7.03	3515.9
10	7.04	3518.8
11	7.11	3552.0
12	7.12	3559.3
13	7.13	3564.2
14	7.15	3572.0
15	7.21	3602.8
16	7.24	3619.4
17	7.27	3633.5
18	7.29	3641.3
19	7.43	3714.1
20	7.45	3722.4
21	7.46	3726.8
22	7.53	3765.8
23	7.54	3768.7
24	7.55	3771.7
25	7.55	3774.6
26	7.58	3787.3
27	7.69	3842.9
28	9.82	4906.5
29	9.83	4914.4

Spectrum 23. ^{1}H NMR of compd **19** (500 MHz, DMSO-d_6).

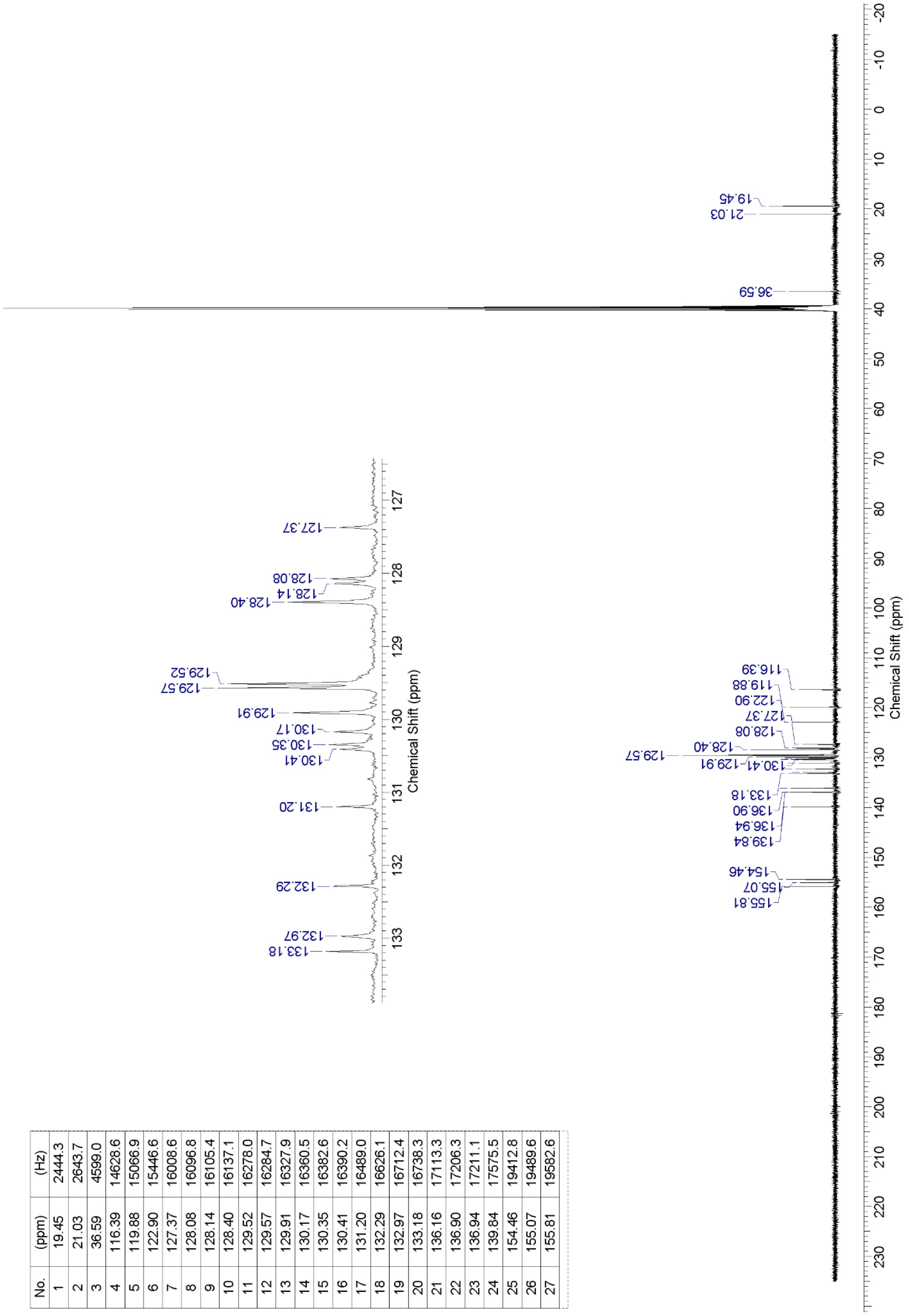

No.	(ppm)	(Hz)
1	19.45	2444.3
2	21.03	2643.7
3	36.59	4599.0
4	116.39	14628.6
5	119.88	15066.9
6	122.90	15446.6
7	127.37	16008.6
8	128.08	16096.8
9	128.14	16105.4
10	128.40	16137.1
11	129.52	16278.0
12	129.57	16284.7
13	129.91	16327.9
14	130.17	16360.5
15	130.35	16382.6
16	130.41	16390.2
17	131.20	16489.0
18	132.29	16626.1
19	132.97	16712.4
20	133.18	16738.3
21	136.16	17113.3
22	136.90	17206.3
23	136.94	17211.1
24	139.84	17575.5
25	154.46	19412.8
26	155.07	19489.6
27	155.81	19582.6

Spectrum 24. ^{13}C NMR of compd **19** (125 MHz, DMSO-*d*$_6$).

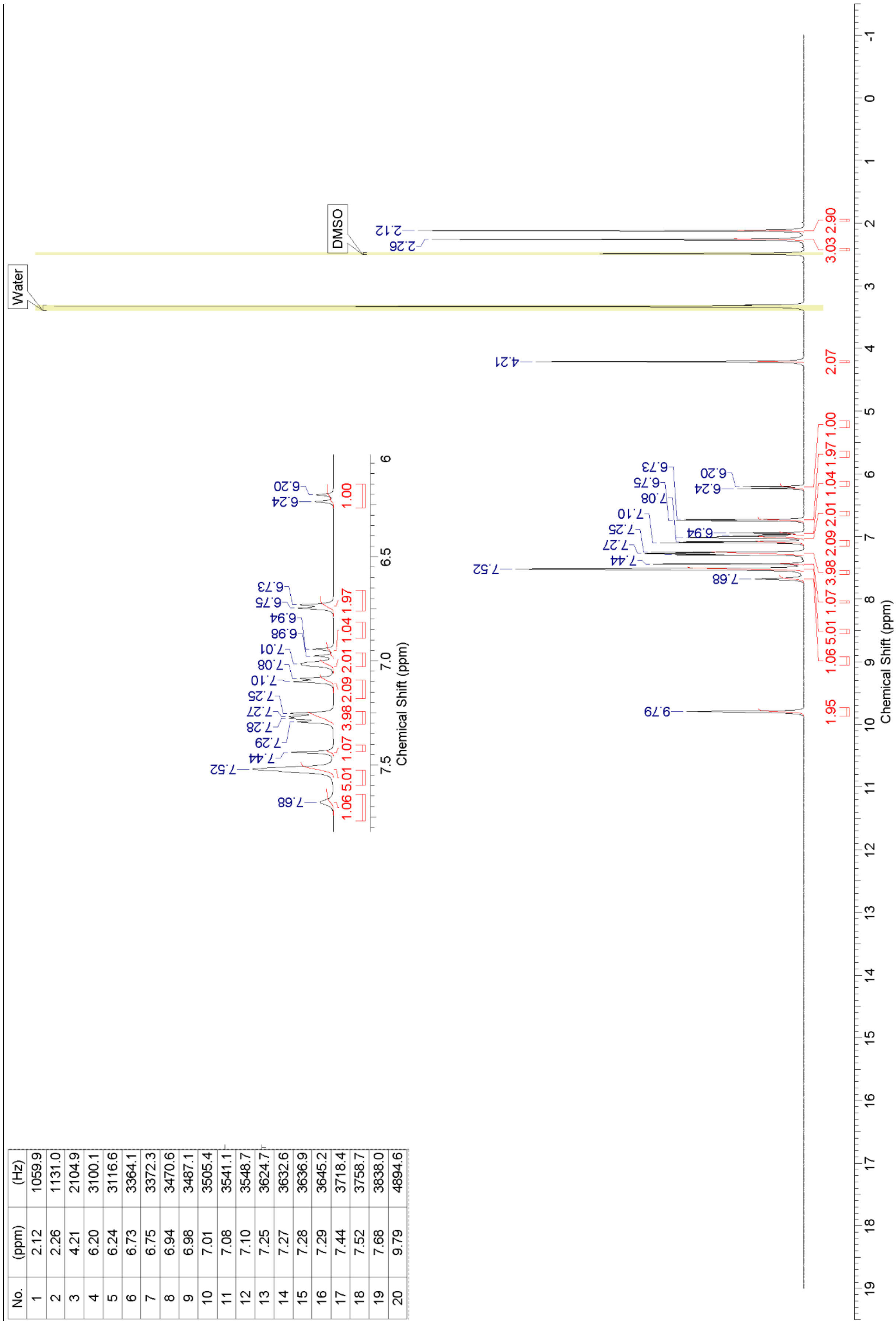

No.	(ppm)	(Hz)
1	2.12	1059.9
2	2.26	1131.0
3	4.21	2104.9
4	6.20	3100.1
5	6.24	3116.6
6	6.73	3364.1
7	6.75	3372.3
8	6.94	3470.6
9	6.98	3487.1
10	7.01	3505.4
11	7.08	3541.1
12	7.10	3548.7
13	7.25	3624.7
14	7.27	3632.6
15	7.28	3636.9
16	7.29	3645.2
17	7.44	3718.4
18	7.52	3758.7
19	7.68	3838.0
20	9.79	4894.6

Spectrum 25. ^{1}H NMR of compd **20** (500 MHz, DMSO-d_6).

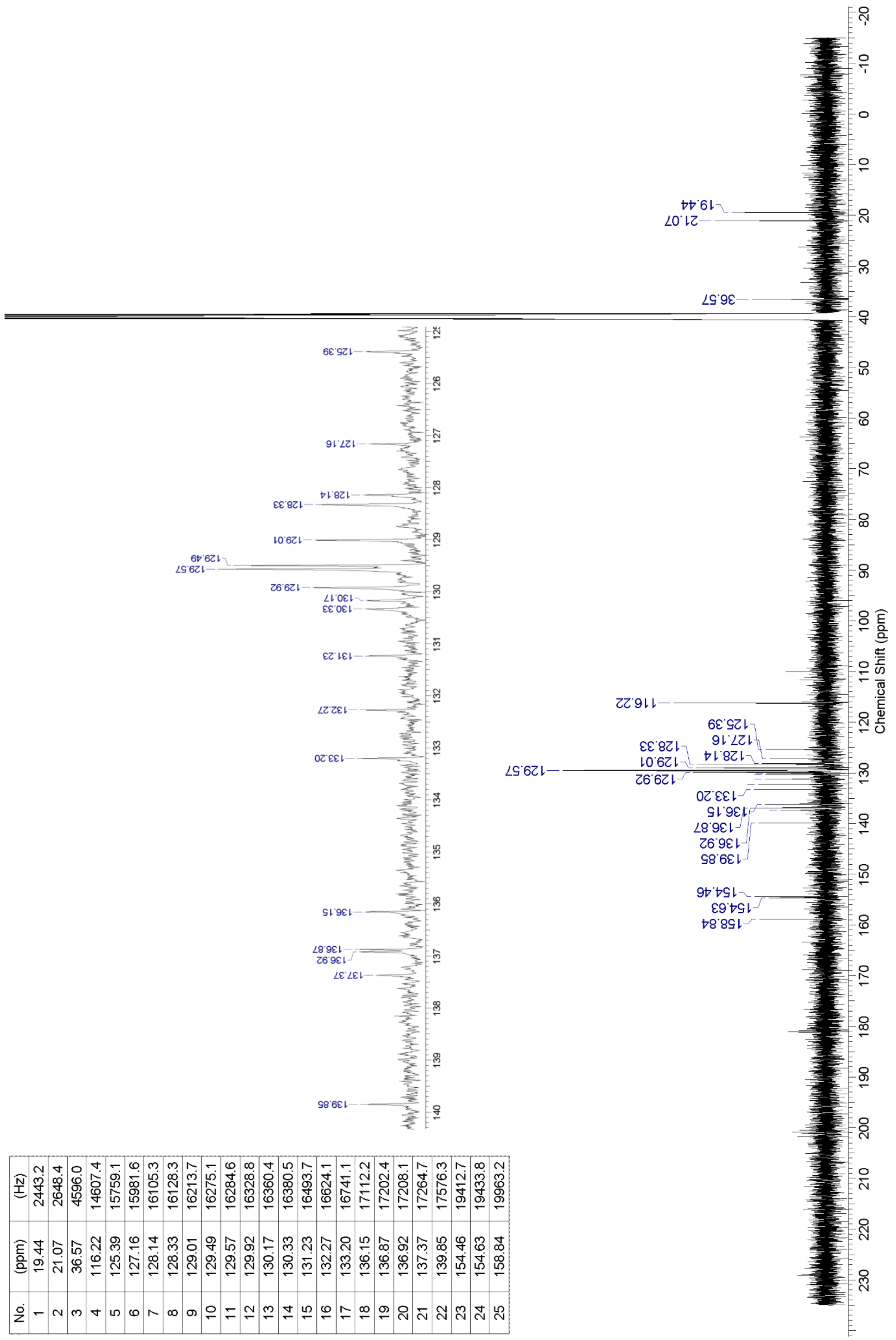

No.	(ppm)	(Hz)
1	19.44	2443.2
2	21.07	2648.4
3	36.57	4596.0
4	116.22	14607.4
5	125.39	15759.1
6	127.16	15981.6
7	128.14	16105.3
8	128.33	16128.3
9	129.01	16213.7
10	129.49	16275.1
11	129.57	16284.6
12	129.92	16328.8
13	130.17	16360.4
14	130.33	16380.5
15	131.23	16493.7
16	132.27	16624.1
17	133.20	16741.1
18	136.15	17112.2
19	136.87	17202.4
20	136.92	17208.1
21	137.37	17264.7
22	139.85	17576.3
23	154.46	19412.7
24	154.63	19433.8
25	158.84	19963.2

Spectrum 26. ^{13}C NMR of compd **20** (125 MHz, DMSO-d_6).

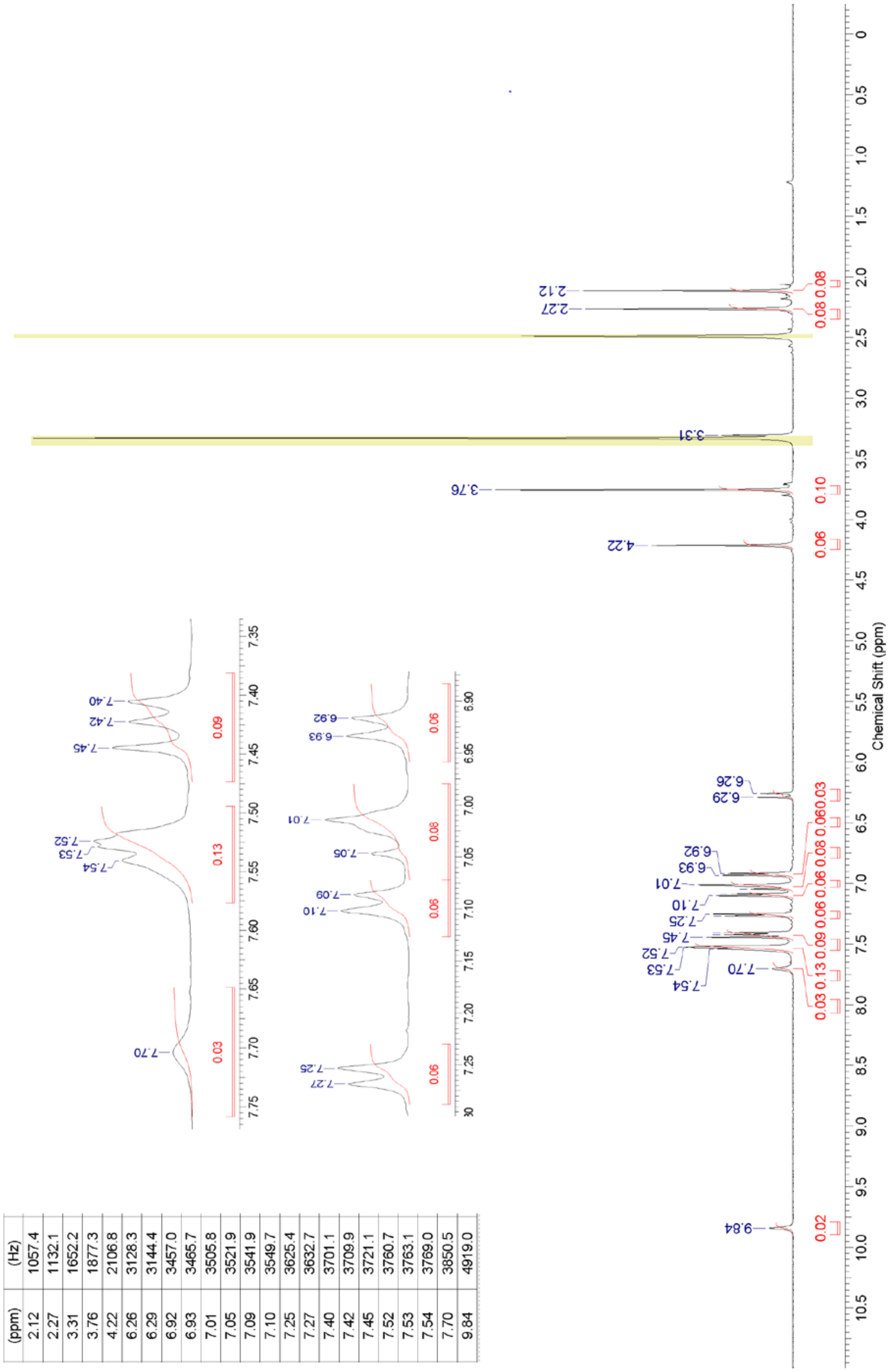

(ppm)	(Hz)
2.12	1057.4
2.27	1132.1
3.31	1652.2
3.76	1877.3
4.22	2106.8
6.26	3128.3
6.29	3144.4
6.92	3457.0
6.93	3465.7
7.01	3505.8
7.05	3521.9
7.09	3541.9
7.10	3549.7
7.25	3625.4
7.27	3632.7
7.40	3701.1
7.42	3709.9
7.45	3721.1
7.52	3760.7
7.53	3763.1
7.54	3769.0
7.70	3850.5
9.84	4919.0

Spectrum 27. ^{1}H NMR of compd **21** (500 MHz, DMSO-d_6).

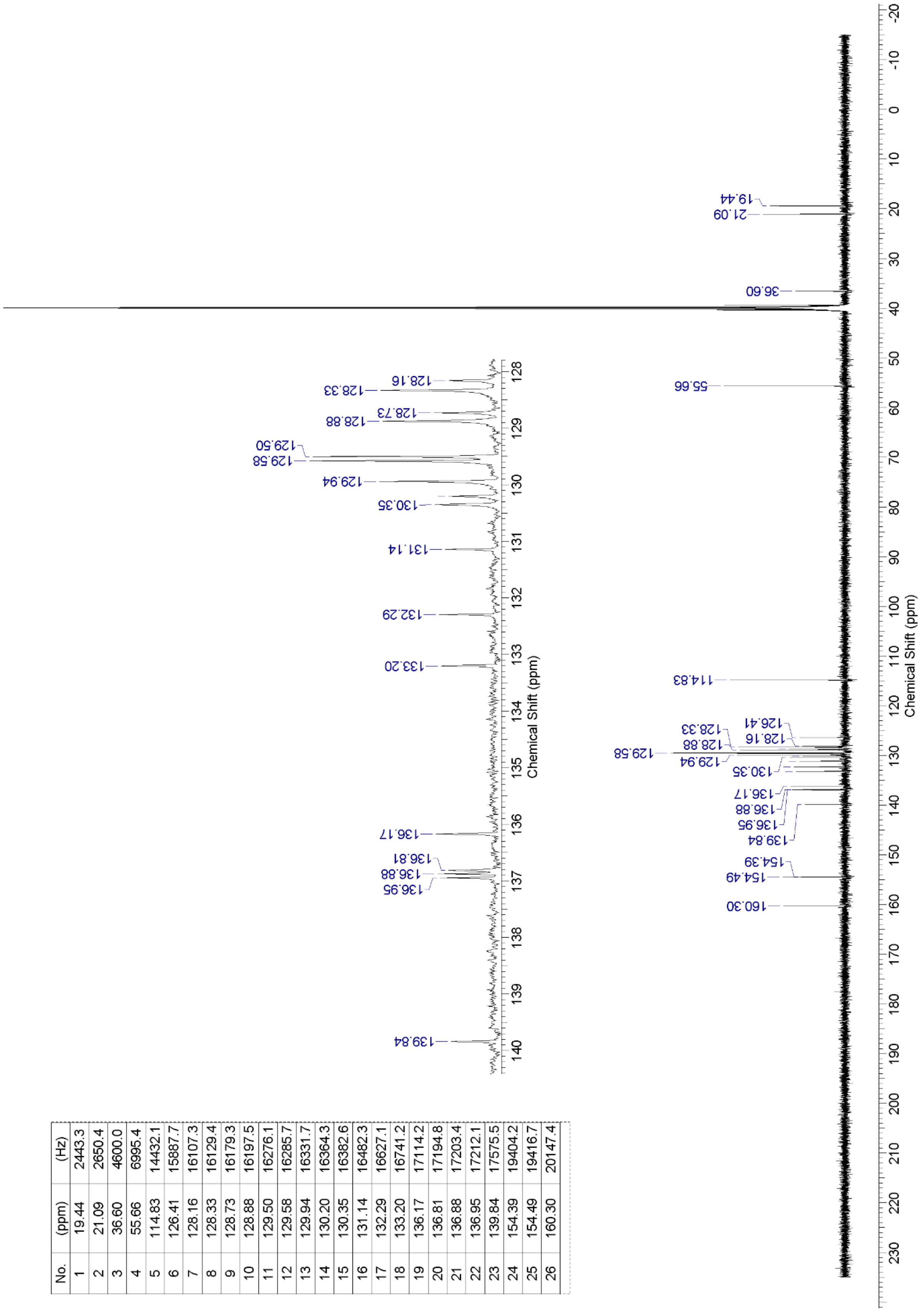

No.	(ppm)	(Hz)
1	19.44	2443.3
2	21.09	2650.4
3	36.60	4600.0
4	55.66	6995.4
5	114.83	14432.1
6	126.41	15887.7
7	128.16	16107.3
8	128.33	16129.4
9	128.73	16179.3
10	128.88	16197.5
11	129.50	16276.1
12	129.58	16285.7
13	129.94	16331.7
14	130.20	16364.3
15	130.35	16382.6
16	131.14	16482.3
17	132.29	16627.1
18	133.20	16741.2
19	136.17	17114.2
20	136.81	17194.8
21	136.88	17203.4
22	136.95	17212.1
23	139.84	17575.5
24	154.39	19404.2
25	154.49	19416.7
26	160.30	20147.4

Spectrum 28. ^{13}C NMR of compd **21** (125 MHz, DMSO-d_6).

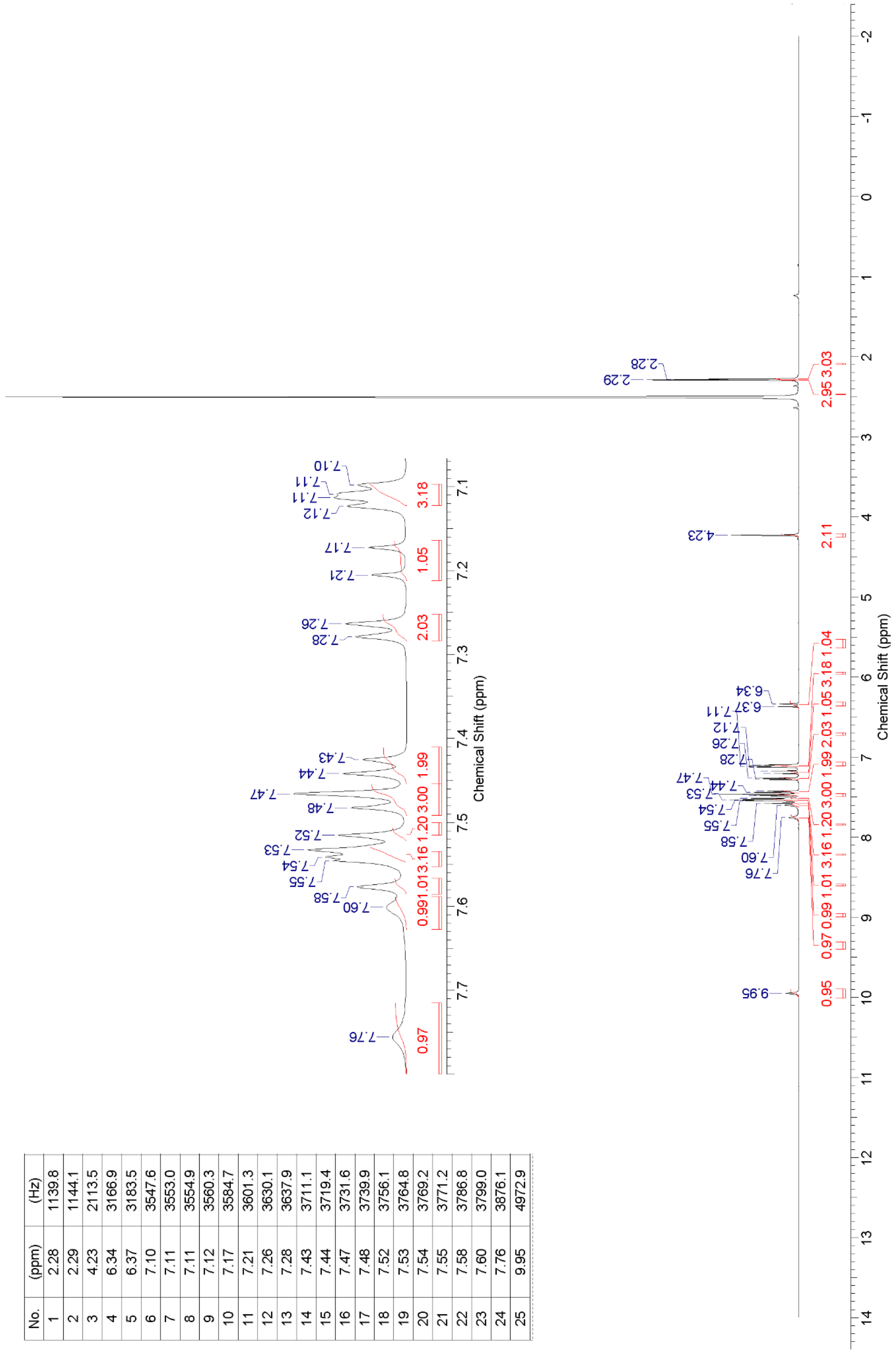

No.	(ppm)	(Hz)
1	2.28	1139.8
2	2.29	1144.1
3	4.23	2113.5
4	6.34	3166.9
5	6.37	3183.5
6	7.10	3547.6
7	7.11	3553.0
8	7.11	3554.9
9	7.12	3560.3
10	7.17	3584.7
11	7.21	3601.3
12	7.26	3630.1
13	7.28	3637.9
14	7.43	3711.1
15	7.44	3719.4
16	7.47	3731.6
17	7.48	3739.9
18	7.52	3756.1
19	7.53	3764.8
20	7.54	3769.2
21	7.55	3771.2
22	7.58	3786.8
23	7.60	3799.0
24	7.76	3876.1
25	9.95	4972.9

Spectrum 29. ^{1}H NMR of compd **22** (500 MHz, DMSO-d_6).

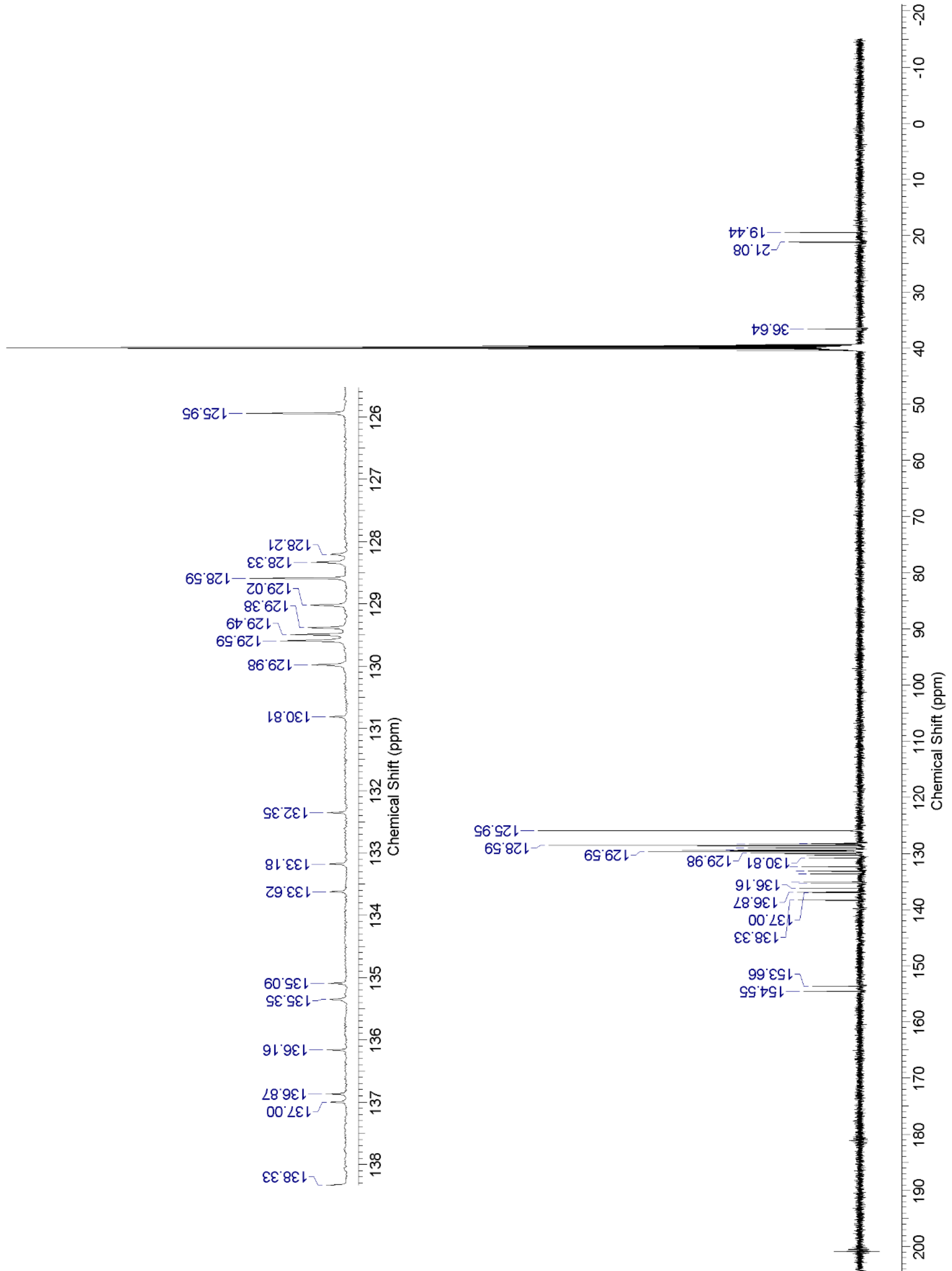

No.	(ppm)	(Hz)
1	19.44	2443.2
2	21.08	2649.4
3	36.64	4604.7
4	125.95	15829.1
5	128.21	16114.0
6	128.33	16129.3
7	128.59	16161.9
8	129.02	16215.6
9	129.38	16260.7
10	129.49	16275.1
11	129.59	16287.5
12	129.98	16336.4
13	130.81	16441.0
14	132.35	16634.7
15	133.18	16738.2
16	133.62	16793.8
17	135.09	16978.9
18	135.35	17011.5
19	136.16	17113.2
20	136.87	17202.4
21	137.00	17218.7
22	138.33	17385.5
23	153.66	19312.0
24	154.55	19424.2

Spectrum 30. ^{13}C NMR of compd **22** (125 MHz, DMSO-d_6).

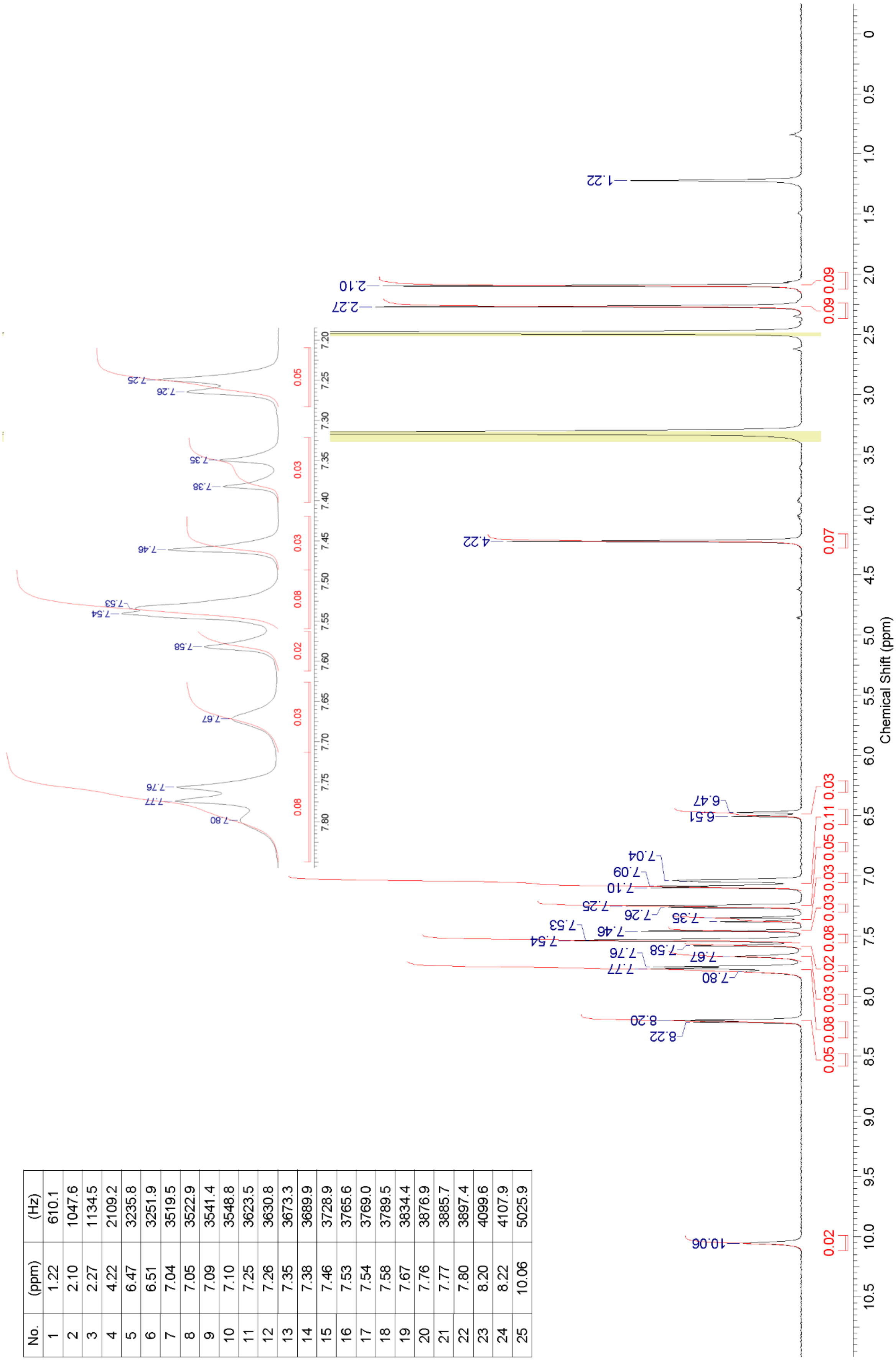

No.	(ppm)	(Hz)
1	1.22	610.1
2	2.10	1047.6
3	2.27	1134.5
4	4.22	2109.2
5	6.47	3235.8
6	6.51	3251.9
7	7.04	3519.5
8	7.05	3522.9
9	7.09	3541.4
10	7.10	3548.8
11	7.25	3623.5
12	7.26	3630.8
13	7.35	3673.3
14	7.38	3689.9
15	7.46	3728.9
16	7.53	3765.6
17	7.54	3769.0
18	7.58	3789.5
19	7.67	3834.4
20	7.76	3876.9
21	7.77	3885.7
22	7.80	3897.4
23	8.20	4099.6
24	8.22	4107.9
25	10.06	5025.9

Spectrum 31. ^{1}H NMR of compd **23** (500 MHz, DMSO-*d*$_6$).

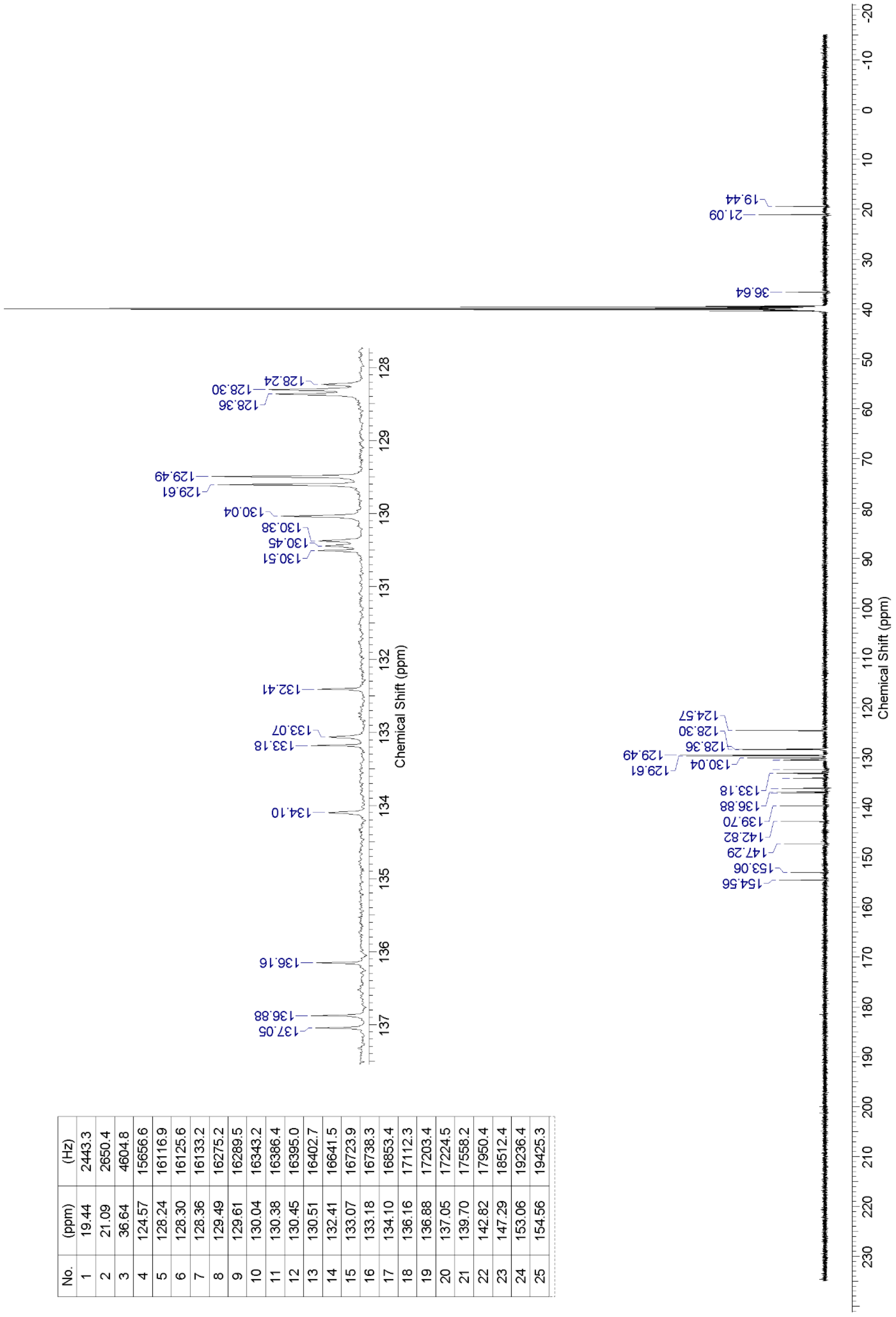

No.	(ppm)	(Hz)
1	19.44	2443.3
2	21.09	2650.4
3	36.64	4604.8
4	124.57	15656.6
5	128.24	16116.9
6	128.30	16125.6
7	128.36	16133.2
8	129.49	16275.2
9	129.61	16289.5
10	130.04	16343.2
11	130.38	16386.4
12	130.45	16395.0
13	130.51	16402.7
14	132.41	16641.5
15	133.07	16723.9
16	133.18	16738.3
17	134.10	16853.4
18	136.16	17112.3
19	136.88	17203.4
20	137.05	17224.5
21	139.70	17558.2
22	142.82	17950.4
23	147.29	18512.4
24	153.06	19236.4
25	154.56	19425.3

Spectrum 32. ^{13}C NMR of compd **23** (125 MHz, DMSO-d_6).

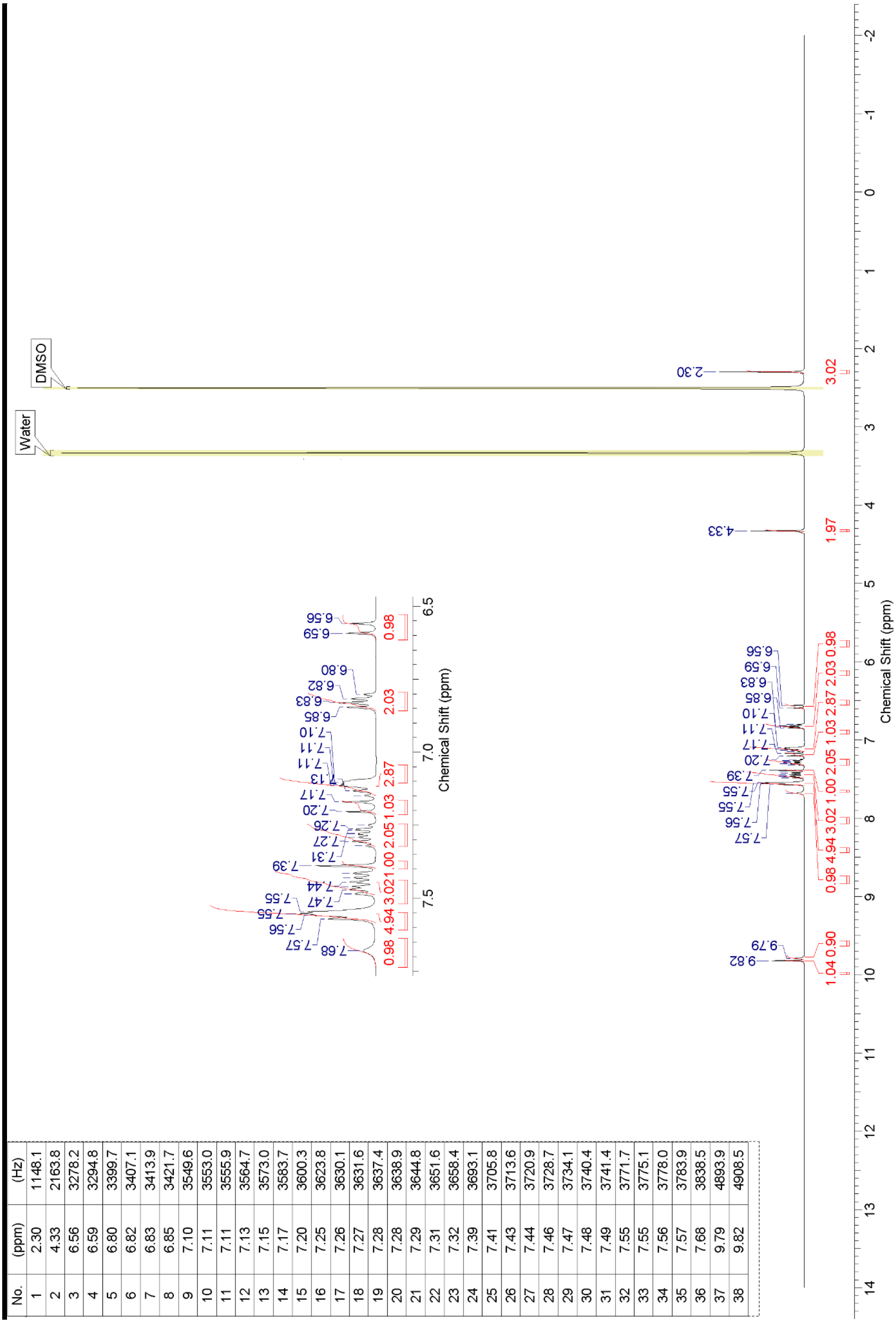

No.	(ppm)	(Hz)
1	2.30	1148.1
2	4.33	2163.8
3	6.56	3278.2
4	6.59	3294.8
5	6.80	3399.7
6	6.82	3407.1
7	6.83	3413.9
8	6.85	3421.7
9	7.10	3549.6
10	7.11	3553.0
11	7.11	3555.9
12	7.13	3564.7
13	7.15	3573.0
14	7.17	3583.7
15	7.20	3600.3
16	7.25	3623.8
17	7.26	3630.1
18	7.27	3631.6
19	7.28	3637.4
20	7.28	3638.9
21	7.29	3644.8
22	7.31	3651.6
23	7.32	3658.4
24	7.39	3693.1
25	7.41	3705.8
26	7.43	3713.6
27	7.44	3720.9
28	7.46	3728.7
29	7.47	3734.1
30	7.48	3740.4
31	7.49	3741.4
32	7.55	3771.7
33	7.55	3775.1
34	7.56	3778.0
35	7.57	3783.9
36	7.68	3838.5
37	9.79	4893.9
38	9.82	4908.5

Spectrum 33. ^{1}H NMR of compd **24** (500 MHz, DMSO-d_6).

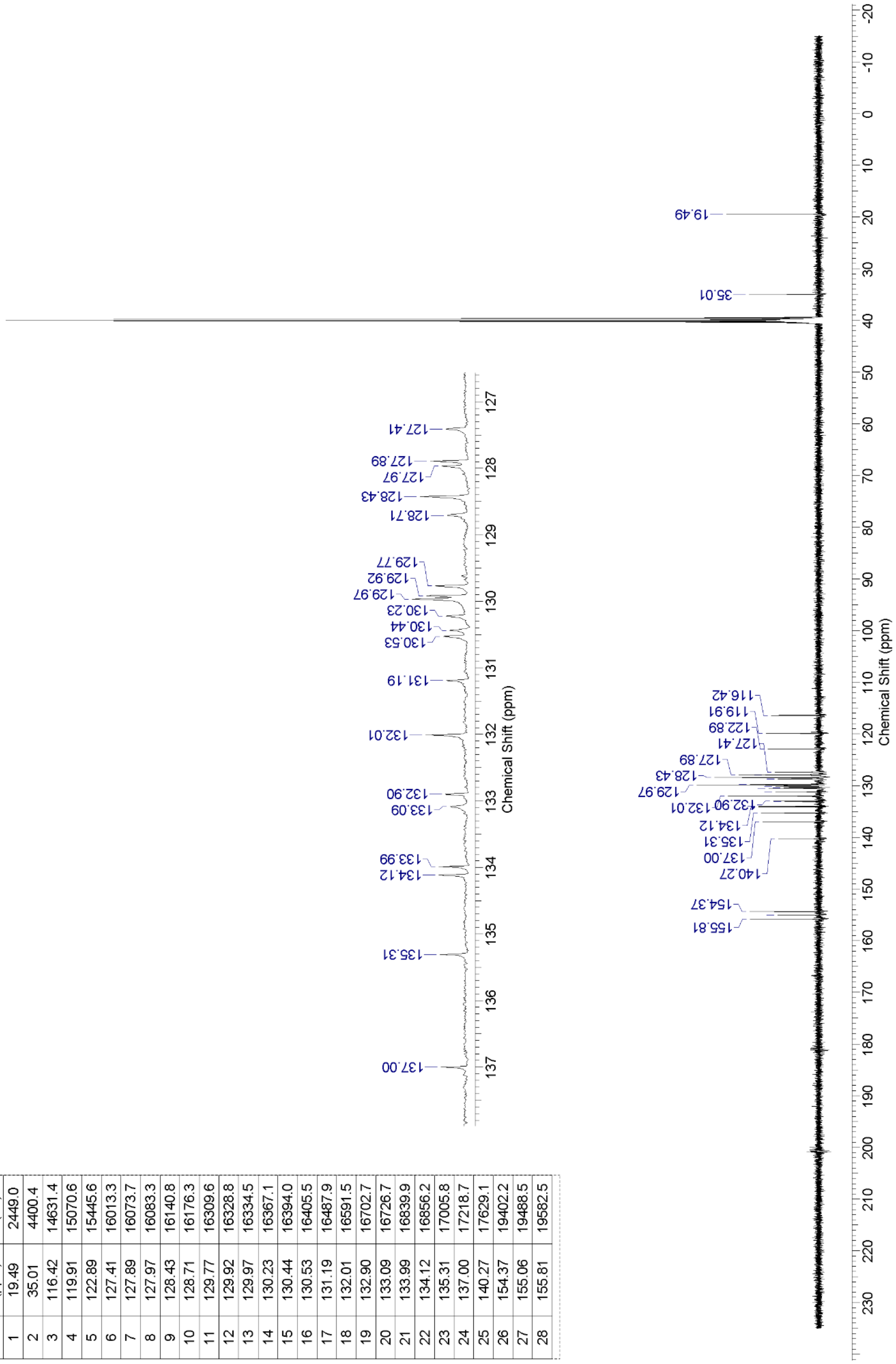

No.	(ppm)	(Hz)
1	19.49	2449.0
2	35.01	4400.4
3	116.42	14631.4
4	119.91	15070.6
5	122.89	15445.6
6	127.41	16013.3
7	127.89	16073.7
8	127.97	16083.3
9	128.43	16140.8
10	128.71	16176.3
11	129.77	16309.6
12	129.92	16328.8
13	129.97	16334.5
14	130.23	16367.1
15	130.44	16394.0
16	130.53	16405.5
17	131.19	16487.9
18	132.01	16591.5
19	132.90	16702.7
20	133.09	16726.7
21	133.99	16839.9
22	134.12	16856.2
23	135.31	17005.8
24	137.00	17218.7
25	140.27	17629.1
26	154.37	19402.2
27	155.06	19488.5
28	155.81	19582.5

Spectrum 34. ^{13}C NMR of compd **24** (125 MHz, DMSO-d_6).

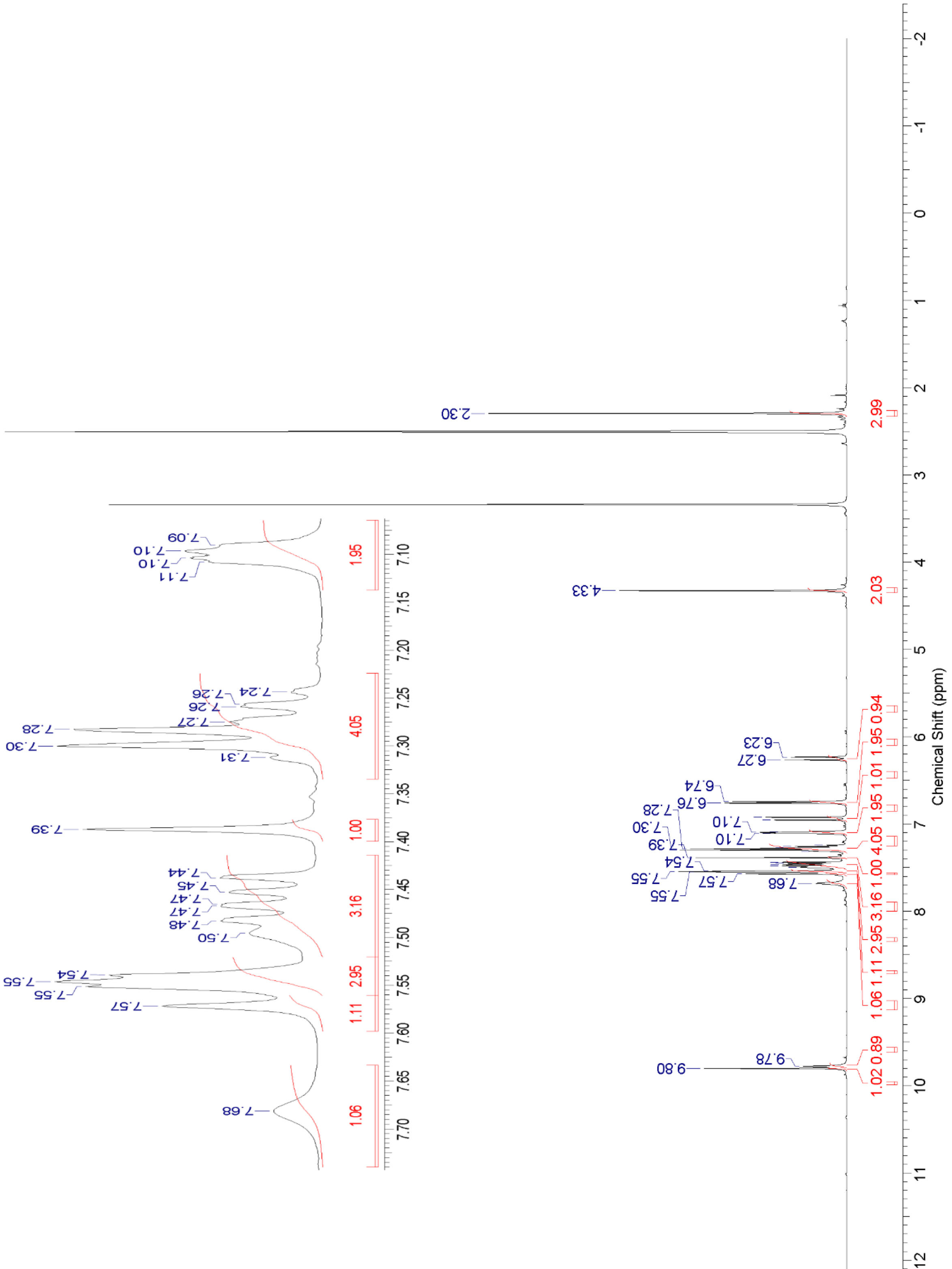

No.	(ppm)	(Hz)
1	2.30	1147.1
2	4.33	2161.9
3	6.23	3115.6
4	6.27	3132.2
5	6.74	3370.9
6	6.76	3379.2
7	6.92	3460.3
8	6.96	3476.4
9	7.09	3543.7
10	7.10	3546.7
11	7.10	3550.6
12	7.11	3552.5
13	7.24	3620.4
14	7.26	3626.7
15	7.26	3628.2
16	7.27	3636.0
17	7.28	3639.9
18	7.30	3648.7
19	7.31	3654.5
20	7.39	3692.1
21	7.44	3717.5
22	7.45	3724.8
23	7.47	3731.2
24	7.47	3732.1
25	7.48	3739.9
26	7.50	3746.3
27	7.54	3768.3
28	7.55	3771.7
29	7.55	3774.1
30	7.57	3784.4
31	7.68	3839.0
32	9.78	4887.0
33	9.80	4899.7

Spectrum 35. ^{1}H NMR of compd **25** (500 MHz, DMSO-d_6).

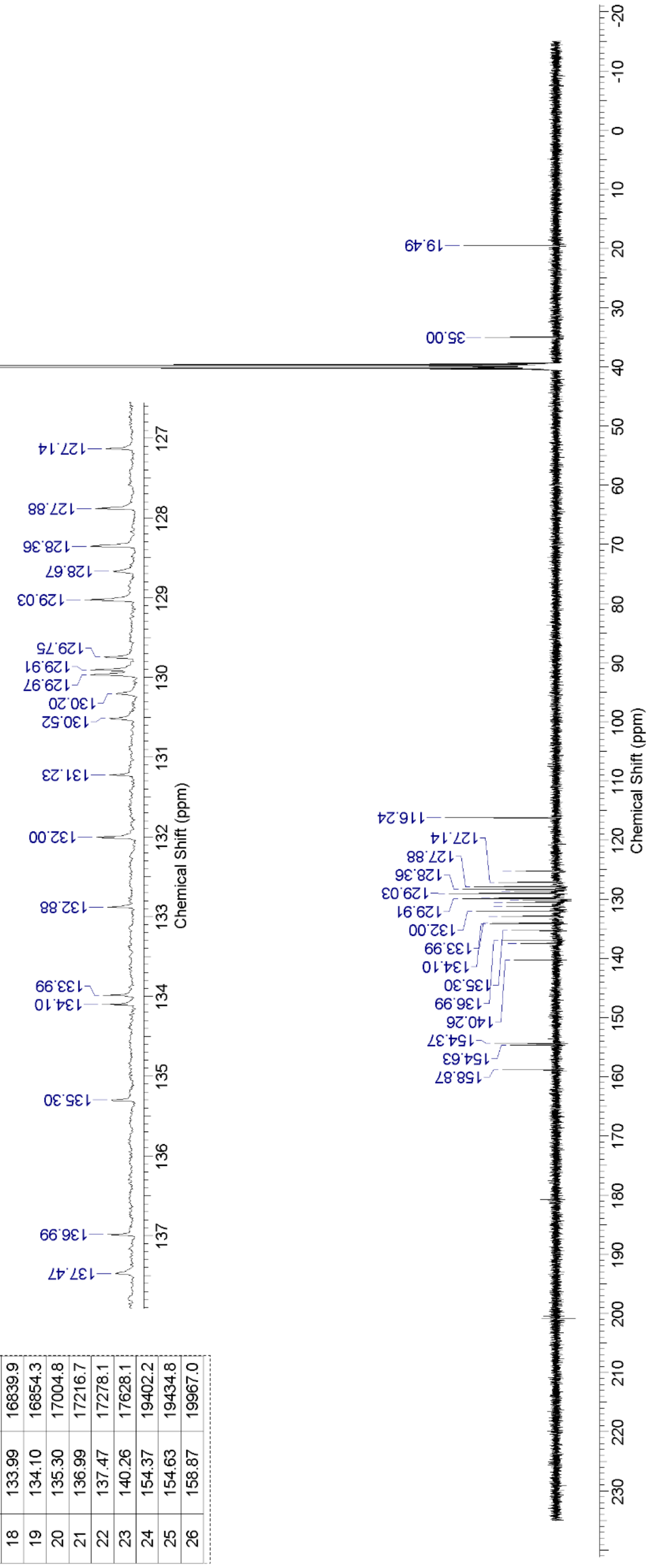

No.	(ppm)	(Hz)
1	19.49	2449.0
2	35.00	4398.5
3	116.24	14609.4
4	125.25	15741.9
5	127.14	15978.7
6	127.88	16072.7
7	128.36	16132.2
8	128.67	16171.5
9	129.03	16216.6
10	129.75	16306.7
11	129.91	16326.8
12	129.97	16334.5
13	130.20	16364.2
14	130.52	16403.6
15	131.23	16492.7
16	132.00	16590.6
17	132.88	16700.8
18	133.99	16839.9
19	134.10	16854.3
20	135.30	17004.8
21	136.99	17216.7
22	137.47	17278.1
23	140.26	17628.1
24	154.37	19402.2
25	154.63	19434.8
26	158.87	19967.0

Spectrum 36. ^{13}C NMR of compd **25** (125 MHz, DMSO-*d*$_6$).

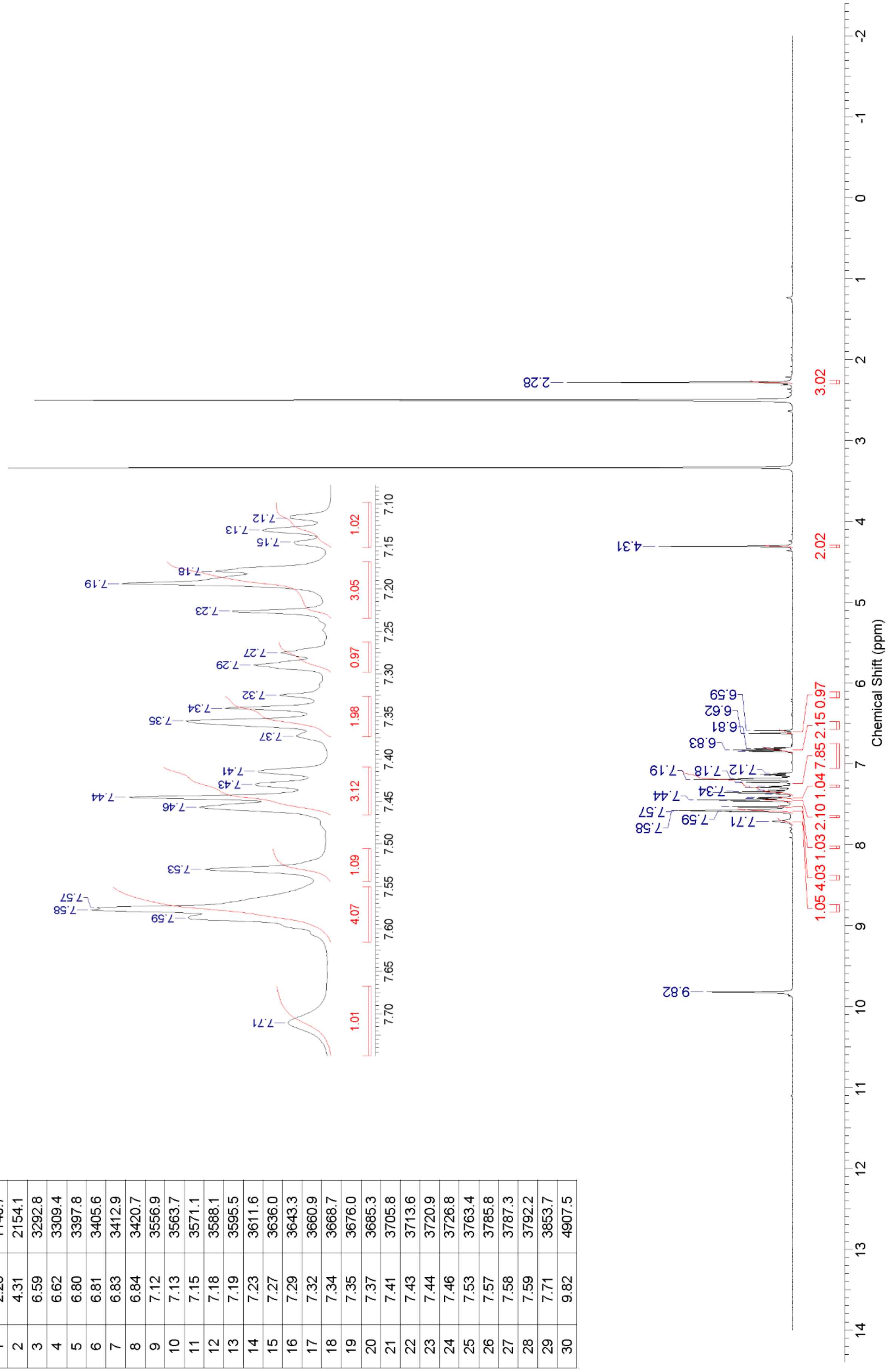

No.	(ppm)	(Hz)
1	2.28	1140.7
2	4.31	2154.1
3	6.59	3292.8
4	6.62	3309.4
5	6.80	3397.8
6	6.81	3405.6
7	6.83	3412.9
8	6.84	3420.7
9	7.12	3556.9
10	7.13	3563.7
11	7.15	3571.1
12	7.18	3588.1
13	7.19	3595.5
14	7.23	3611.6
15	7.27	3636.0
16	7.29	3643.3
17	7.32	3660.9
18	7.34	3668.7
19	7.35	3676.0
20	7.37	3685.3
21	7.41	3705.8
22	7.43	3713.6
23	7.44	3720.9
24	7.46	3726.8
25	7.53	3763.4
26	7.57	3785.8
27	7.58	3787.3
28	7.59	3792.2
29	7.71	3853.7
30	9.82	4907.5

Spectrum 37. ^{1}H NMR of compd **26** (500 MHz, DMSO-d_6).

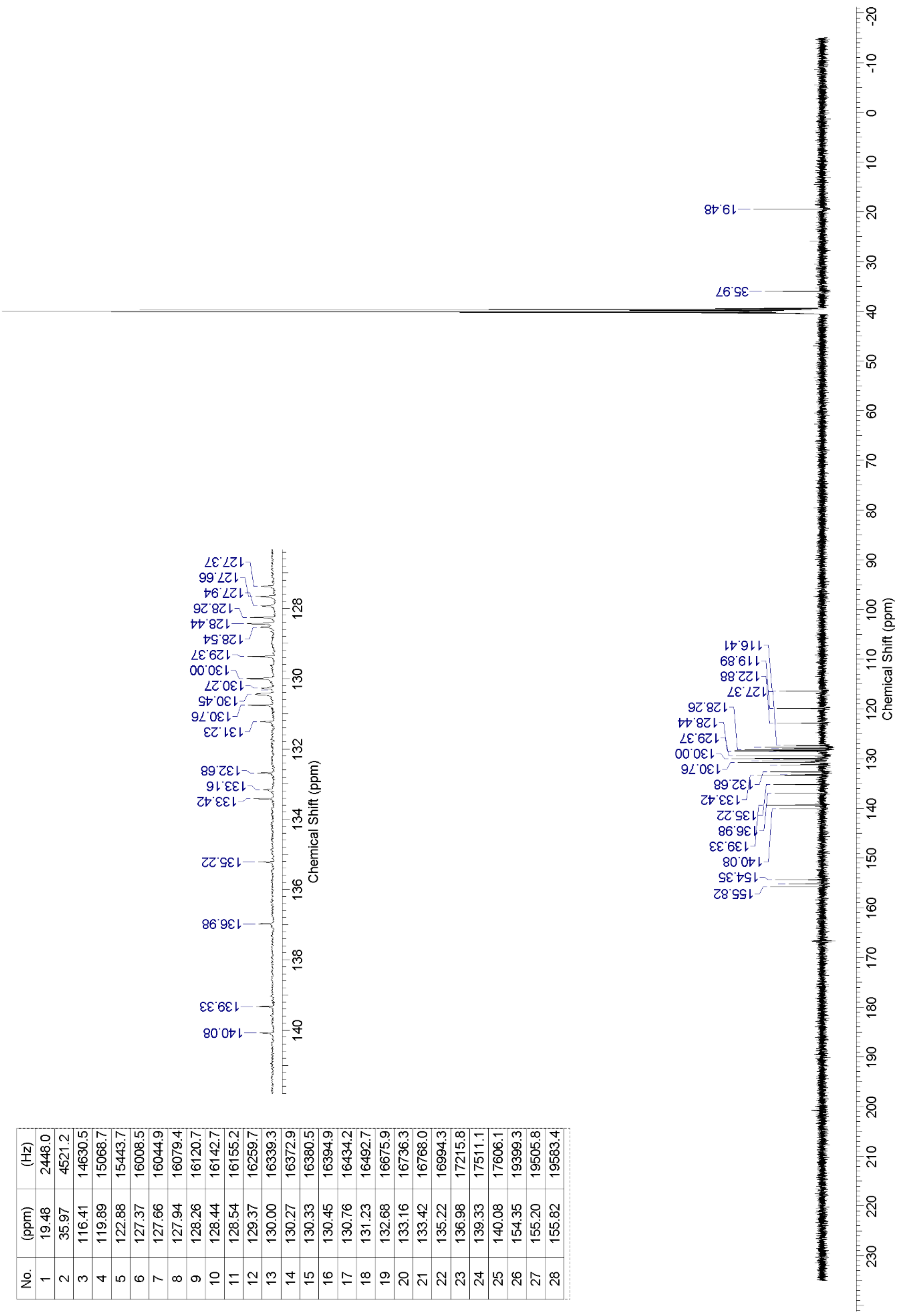

No.	(ppm)	(Hz)
1	19.48	2448.0
2	35.97	4521.2
3	116.41	14630.5
4	119.89	15068.7
5	122.88	15443.7
6	127.37	16008.5
7	127.66	16044.9
8	127.94	16079.4
9	128.26	16120.7
10	128.44	16142.7
11	128.54	16155.2
12	129.37	16259.7
13	130.00	16339.3
14	130.27	16372.9
15	130.33	16380.5
16	130.45	16394.9
17	130.76	16434.2
18	131.23	16492.7
19	132.68	16675.9
20	133.16	16736.3
21	133.42	16768.0
22	135.22	16994.3
23	136.98	17215.8
24	139.33	17511.1
25	140.08	17606.1
26	154.35	19399.3
27	155.20	19505.8
28	155.82	19583.4

Spectrum 38. ^{13}C NMR of compd **26** (125 MHz, DMSO-d_6).

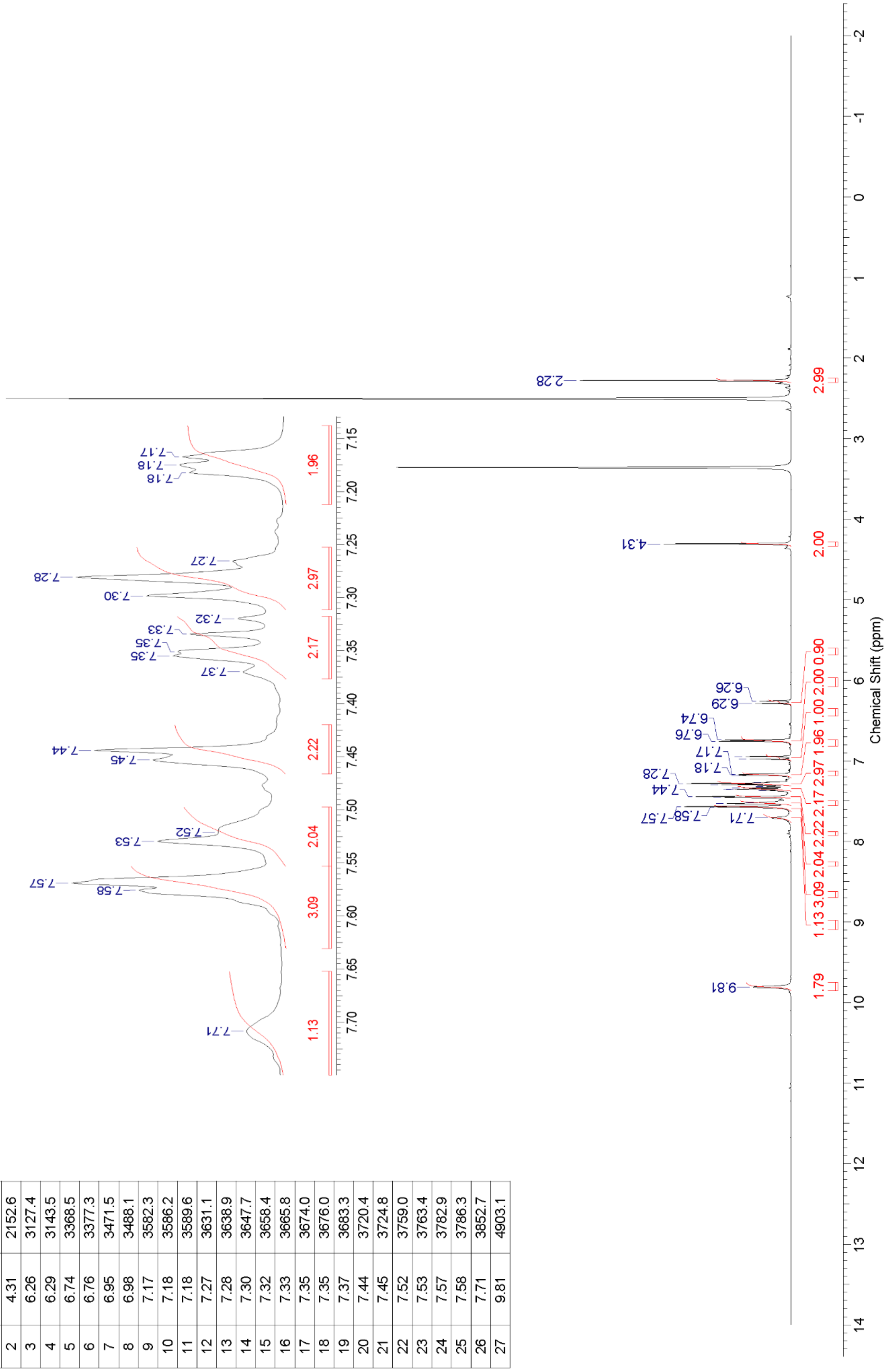

No.	(ppm)	(Hz)
1	2.28	1139.3
2	4.31	2152.6
3	6.26	3127.4
4	6.29	3143.5
5	6.74	3368.5
6	6.76	3377.3
7	6.95	3471.5
8	6.98	3488.1
9	7.17	3582.3
10	7.18	3586.2
11	7.18	3589.6
12	7.27	3631.1
13	7.28	3638.9
14	7.30	3647.7
15	7.32	3658.4
16	7.33	3665.8
17	7.35	3674.0
18	7.35	3676.0
19	7.37	3683.3
20	7.44	3720.4
21	7.45	3724.8
22	7.52	3759.0
23	7.53	3763.4
24	7.57	3782.9
25	7.58	3786.3
26	7.71	3852.7
27	9.81	4903.1

Spectrum 39. ^{1}H NMR of compd **27** (500 MHz, DMSO-d_6).

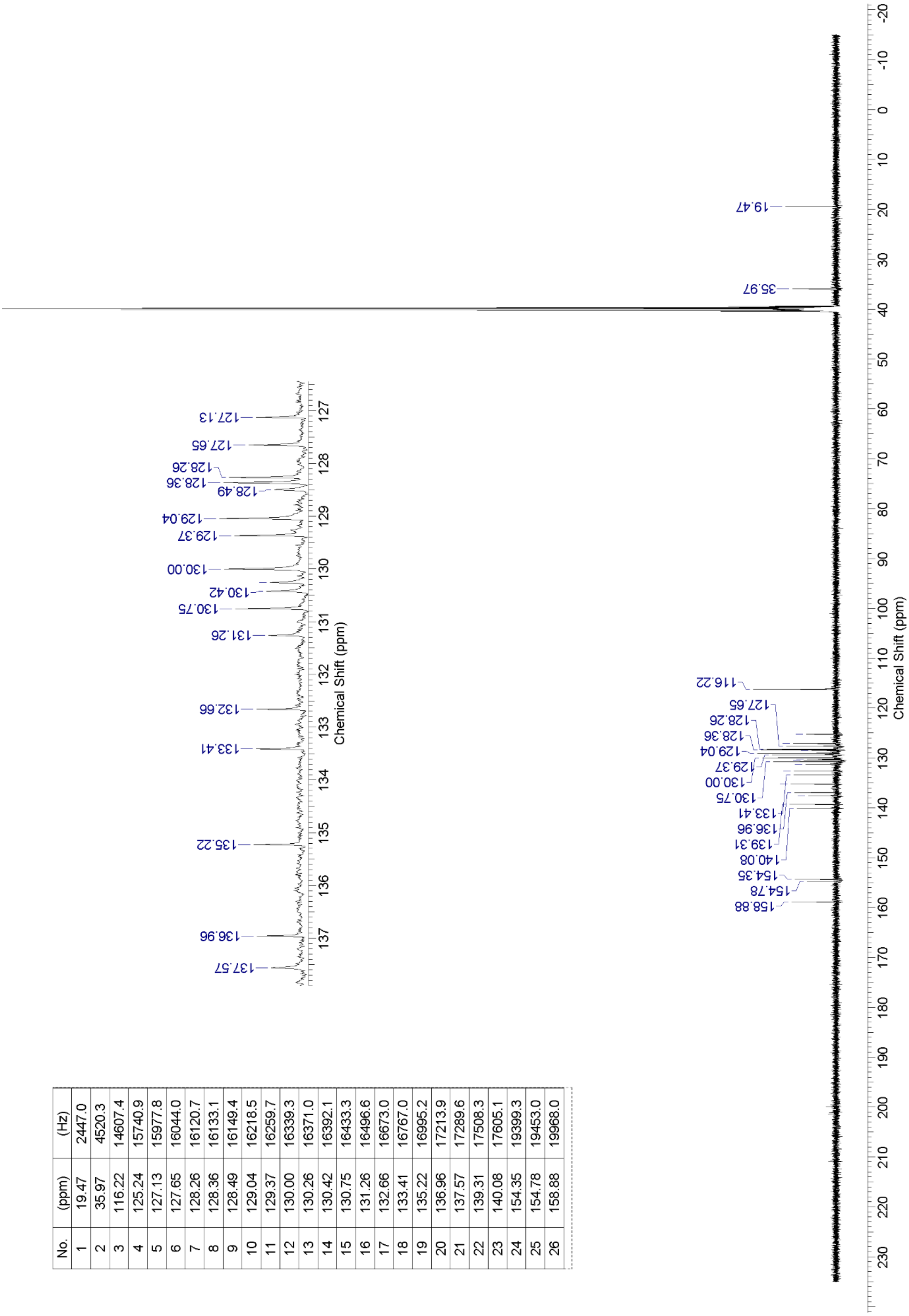

No.	(ppm)	(Hz)
1	19.47	2447.0
2	35.97	4520.3
3	116.22	14607.4
4	125.24	15740.9
5	127.13	15977.8
6	127.65	16044.0
7	128.26	16120.7
8	128.36	16133.1
9	128.49	16149.4
10	129.04	16218.5
11	129.37	16259.7
12	130.00	16339.3
13	130.26	16371.0
14	130.42	16392.1
15	130.75	16433.3
16	131.26	16496.6
17	132.66	16673.0
18	133.41	16767.0
19	135.22	16995.2
20	136.96	17213.9
21	137.57	17289.6
22	139.31	17508.3
23	140.08	17605.1
24	154.35	19399.3
25	154.78	19453.0
26	158.88	19968.0

Spectrum 40. ^{13}C NMR of compd **27** (125 MHz, DMSO-d_6).

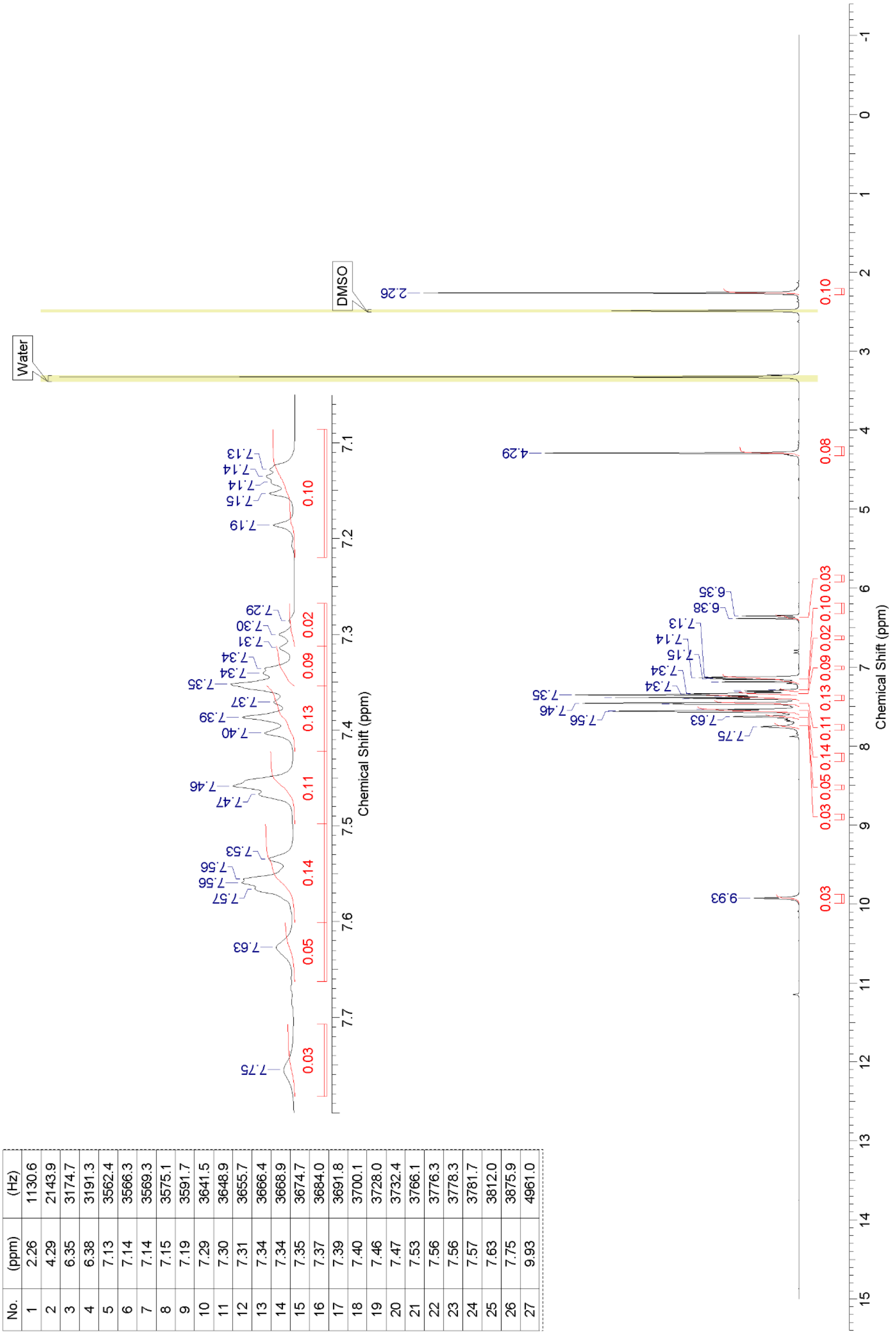

No.	(ppm)	(Hz)
1	2.26	1130.6
2	4.29	2143.9
3	6.35	3174.7
4	6.38	3191.3
5	7.13	3562.4
6	7.14	3566.3
7	7.14	3569.3
8	7.15	3575.1
9	7.19	3591.7
10	7.29	3641.5
11	7.30	3648.9
12	7.31	3655.7
13	7.34	3666.4
14	7.34	3668.9
15	7.35	3674.7
16	7.37	3684.0
17	7.39	3691.8
18	7.40	3700.1
19	7.46	3728.0
20	7.47	3732.4
21	7.53	3766.1
22	7.56	3776.3
23	7.56	3778.3
24	7.57	3781.7
25	7.63	3812.0
26	7.75	3875.9
27	9.93	4961.0

Spectrum 41. ^{1}H NMR of compd **28** (500 MHz, DMSO-d_6).

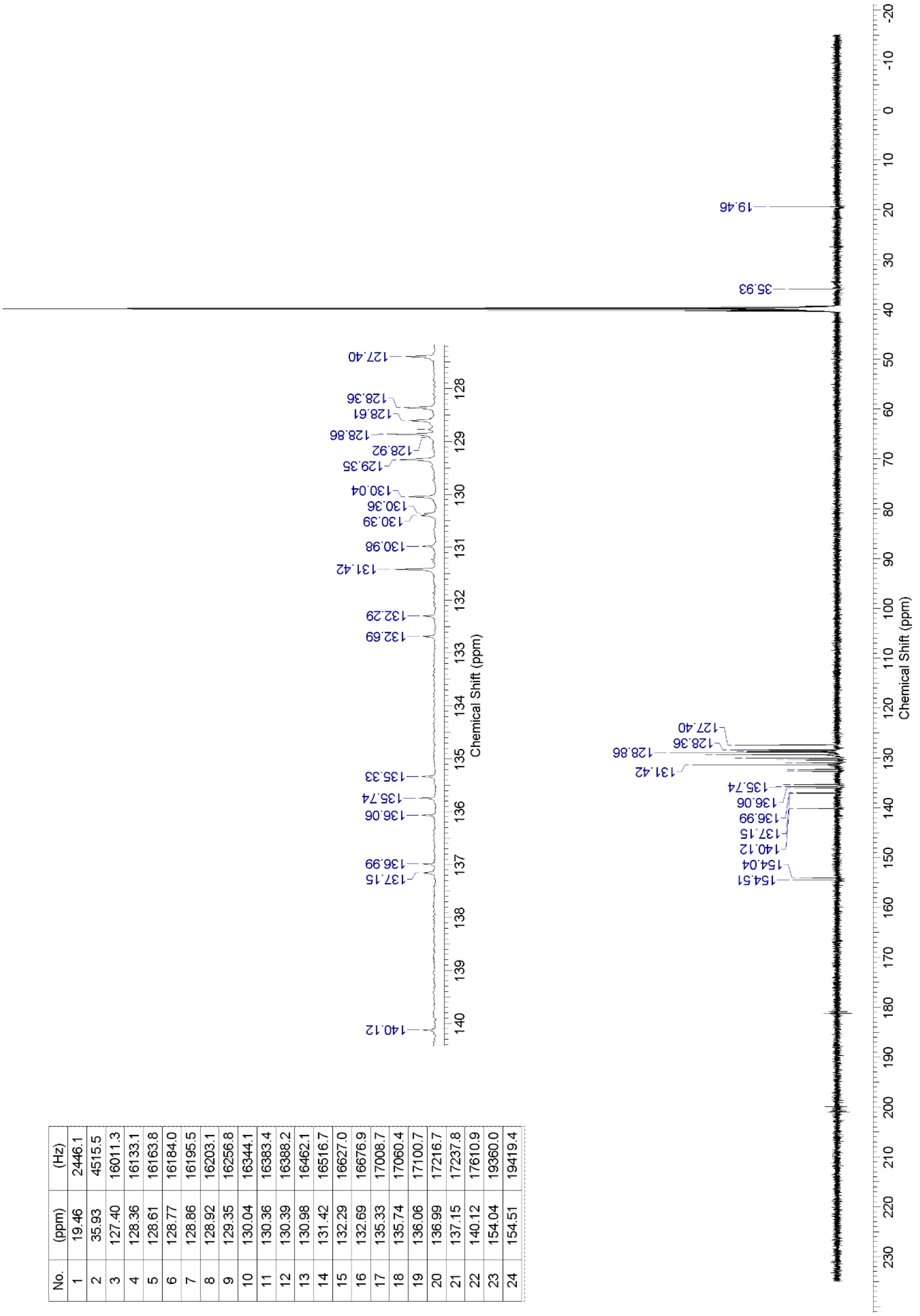

No.	(ppm)	(Hz)
1	19.46	2446.1
2	35.93	4515.5
3	127.40	16011.3
4	128.36	16133.1
5	128.61	16163.8
6	128.77	16184.0
7	128.86	16195.5
8	128.92	16203.1
9	129.35	16256.8
10	130.04	16344.1
11	130.36	16383.4
12	130.39	16388.2
13	130.98	16462.1
14	131.42	16516.7
15	132.29	16627.0
16	132.69	16676.9
17	135.33	17008.7
18	135.74	17060.4
19	136.06	17100.7
20	136.99	17216.7
21	137.15	17237.8
22	140.12	17610.9
23	154.04	19360.0
24	154.51	19419.4

Spectrum 42. ^{13}C NMR of compd **28** (125 MHz, DMSO-d_6).

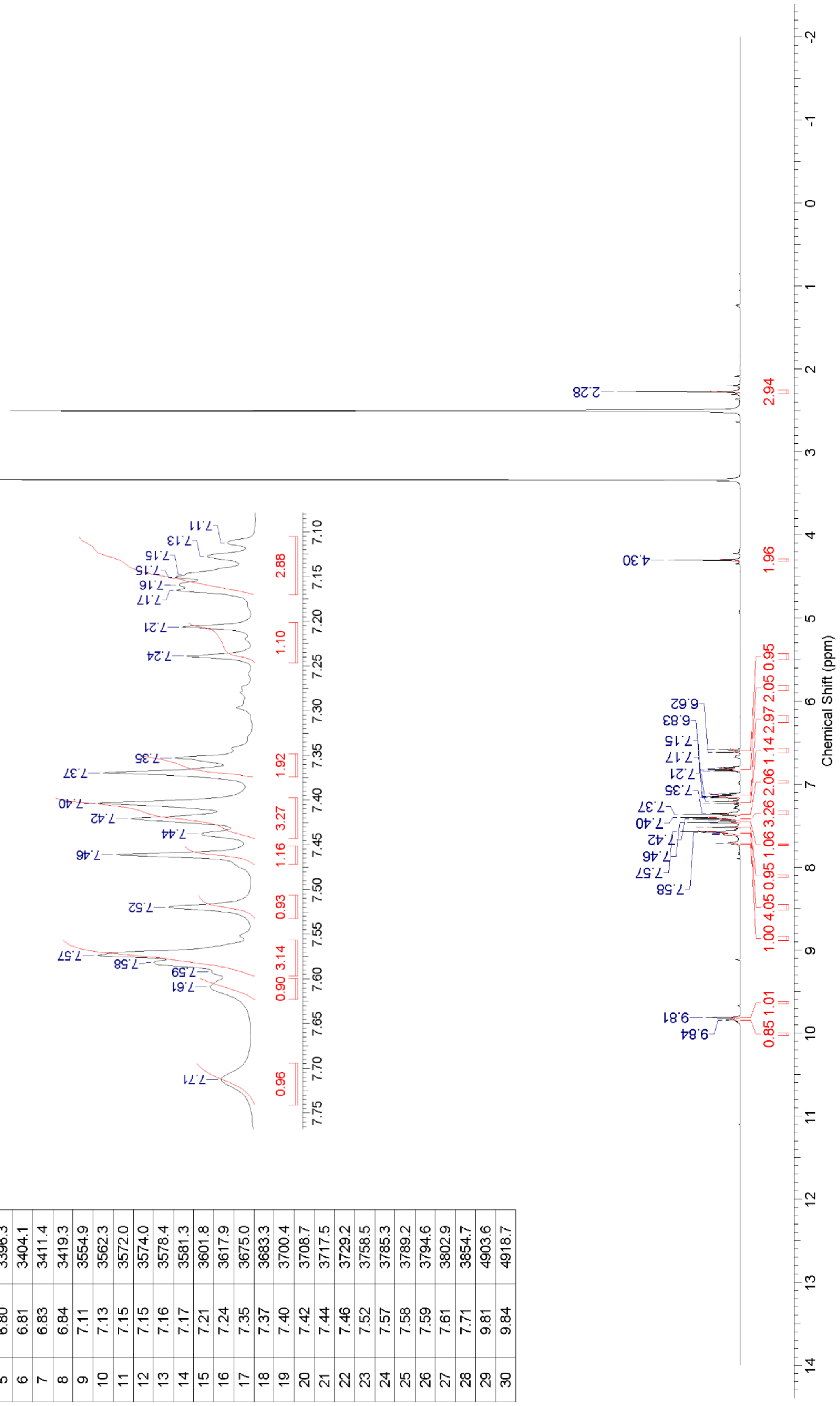

No.	(ppm)	(Hz)
1	2.28	1137.3
2	4.30	2150.2
3	6.59	3291.9
4	6.62	3308.5
5	6.80	3396.3
6	6.81	3404.1
7	6.83	3411.4
8	6.84	3419.3
9	7.11	3554.9
10	7.13	3562.3
11	7.15	3572.0
12	7.15	3574.0
13	7.16	3578.4
14	7.17	3581.3
15	7.21	3601.8
16	7.24	3617.9
17	7.35	3675.0
18	7.37	3683.3
19	7.40	3700.4
20	7.42	3708.7
21	7.44	3717.5
22	7.46	3729.2
23	7.52	3758.5
24	7.57	3785.3
25	7.58	3789.2
26	7.59	3794.6
27	7.61	3802.9
28	7.71	3854.7
29	9.81	4903.6
30	9.84	4918.7

Spectrum 43. ^{1}H NMR of compd **29** (500 MHz, DMSO-d_6).

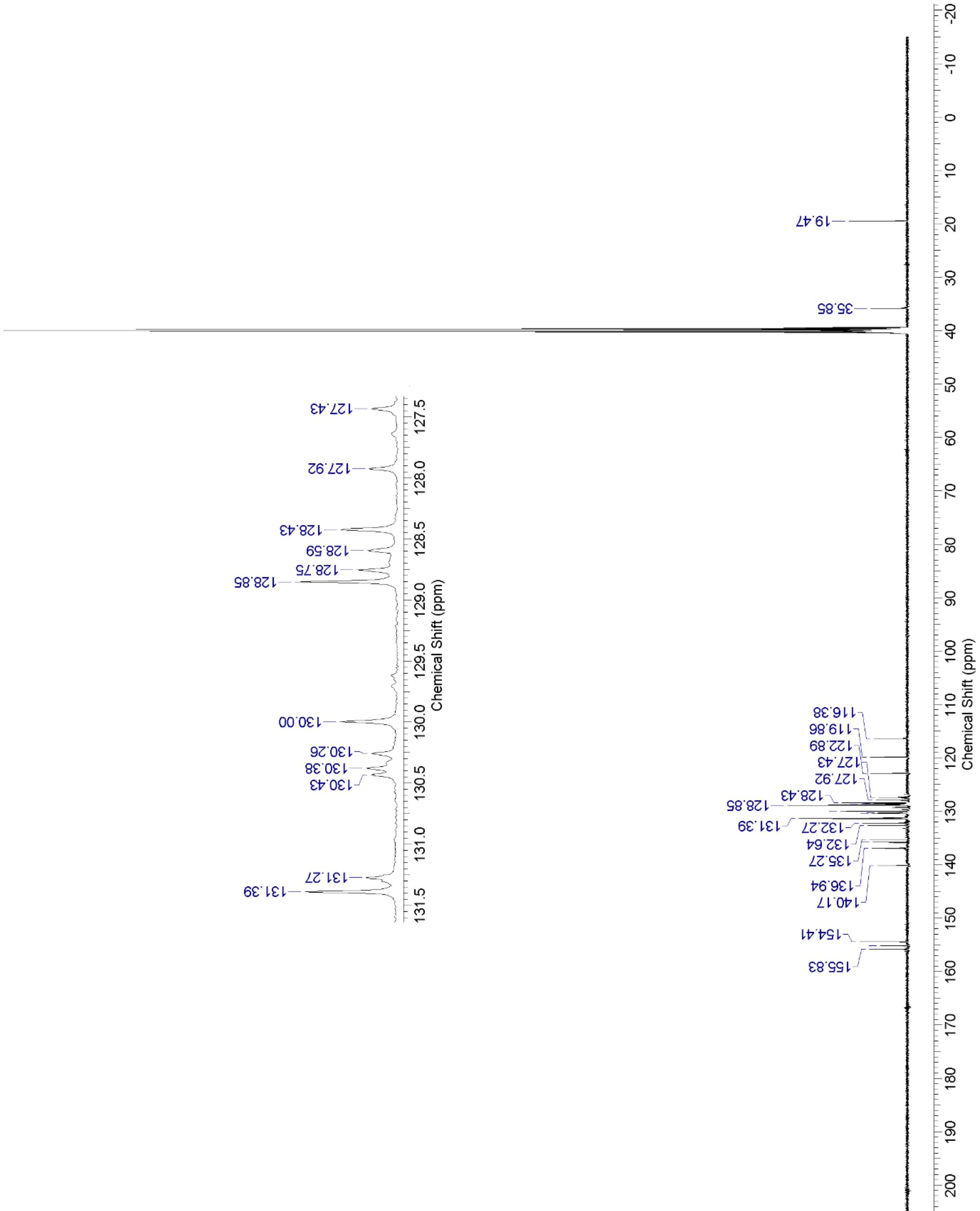

No.	(ppm)	(Hz)
1	19.47	2447.1
2	35.85	4506.0
3	116.38	14626.7
4	119.86	15064.0
5	122.89	15444.7
6	127.43	16016.2
7	127.92	16077.6
8	128.43	16140.9
9	128.59	16162.0
10	128.75	16182.1
11	128.85	16194.6
12	130.00	16338.4
13	130.26	16371.1
14	130.38	16386.4
15	130.43	16393.1
16	131.27	16498.6
17	131.39	16513.0
18	132.27	16624.2
19	132.64	16670.2
20	135.27	17001.1
21	135.80	17067.2
22	136.94	17211.1
23	140.17	17616.7
24	154.41	19407.1
25	155.17	19502.0
26	155.83	19584.5

Spectrum 44. ^{13}C NMR of compd **29** (125 MHz, DMSO-d_6).

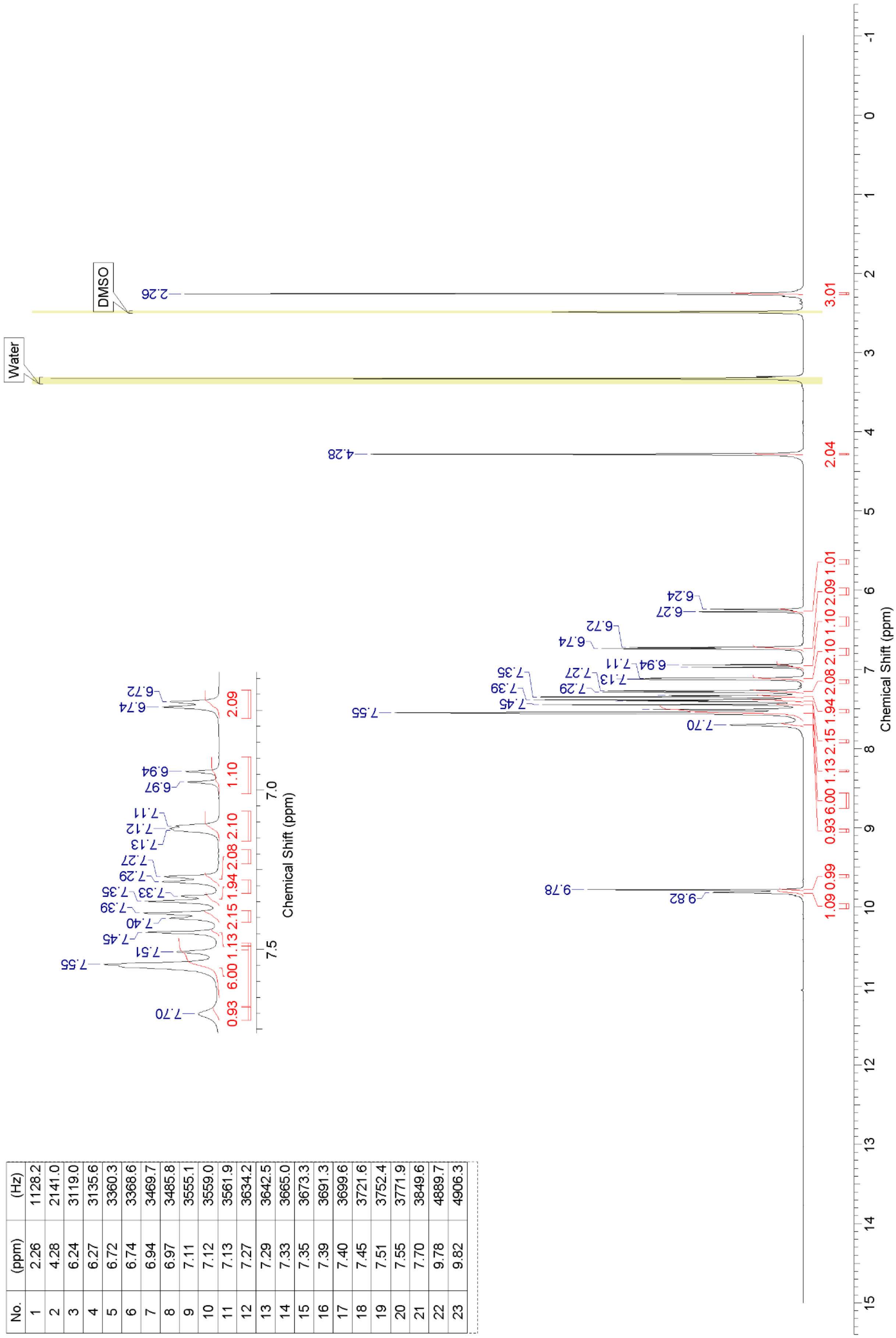

No.	(ppm)	(Hz)
1	2.26	1128.2
2	4.28	2141.0
3	6.24	3119.0
4	6.27	3135.6
5	6.72	3360.3
6	6.74	3368.6
7	6.94	3469.7
8	6.97	3485.8
9	7.11	3555.1
10	7.12	3559.0
11	7.13	3561.9
12	7.27	3634.2
13	7.29	3642.5
14	7.33	3665.0
15	7.35	3673.3
16	7.39	3691.3
17	7.40	3699.6
18	7.45	3721.6
19	7.51	3752.4
20	7.55	3771.9
21	7.70	3849.6
22	9.78	4889.7
23	9.82	4906.3

Spectrum 45. ^{1}H NMR of compd **30** (500 MHz, DMSO-d_6).

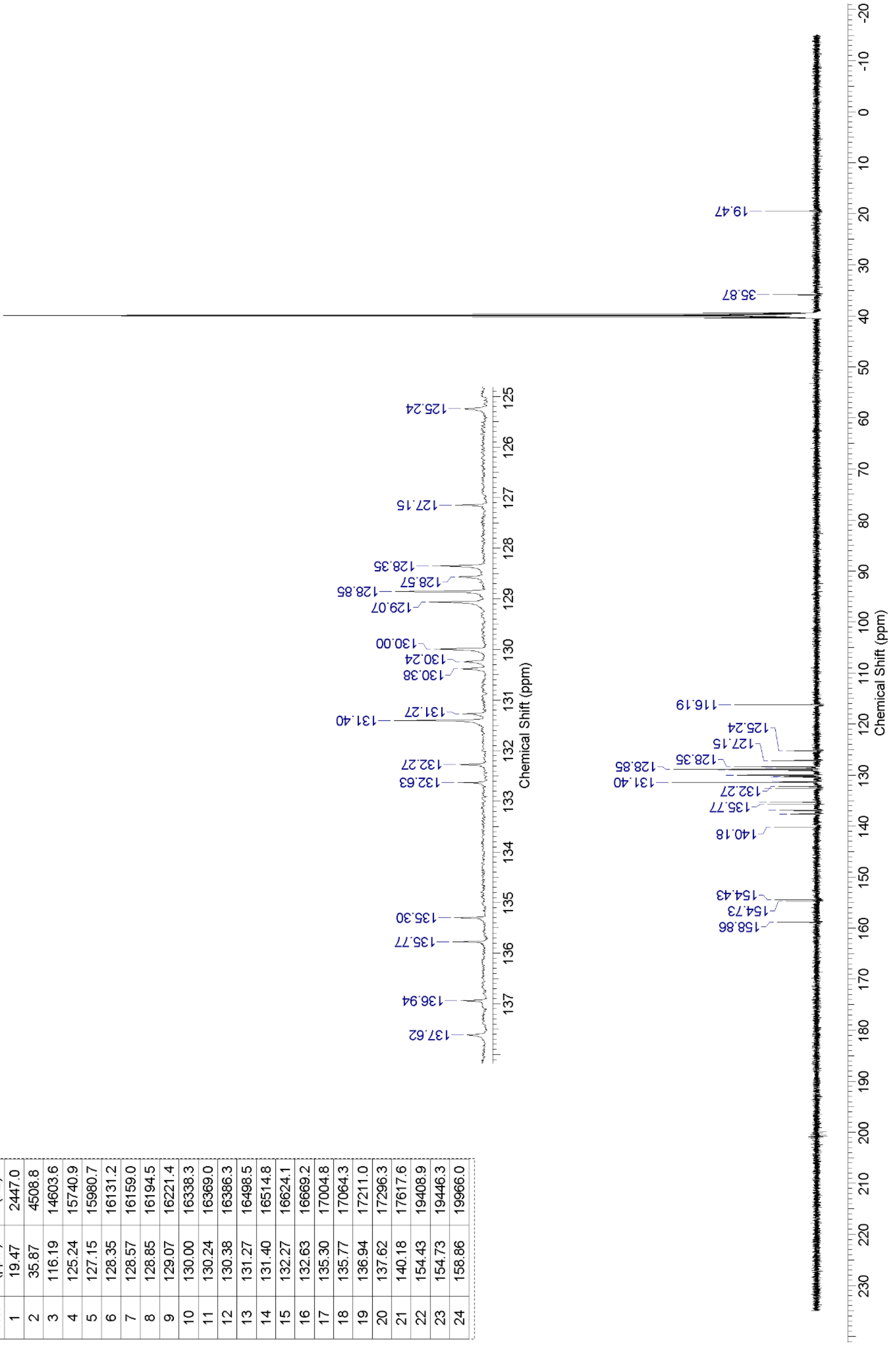

No.	(ppm)	(Hz)
1	19.47	2447.0
2	35.87	4508.8
3	116.19	14603.6
4	125.24	15740.9
5	127.15	15980.7
6	128.35	16131.2
7	128.57	16159.0
8	128.85	16194.5
9	129.07	16221.4
10	130.00	16338.3
11	130.24	16369.0
12	130.38	16386.3
13	131.27	16498.5
14	131.40	16514.8
15	132.27	16624.1
16	132.63	16669.2
17	135.30	17004.8
18	135.77	17064.3
19	136.94	17211.0
20	137.62	17296.3
21	140.18	17617.6
22	154.43	19408.9
23	154.73	19446.3
24	158.86	19966.0

Spectrum 46. ^{13}C NMR of compd **30** (125 MHz, DMSO-d_6).

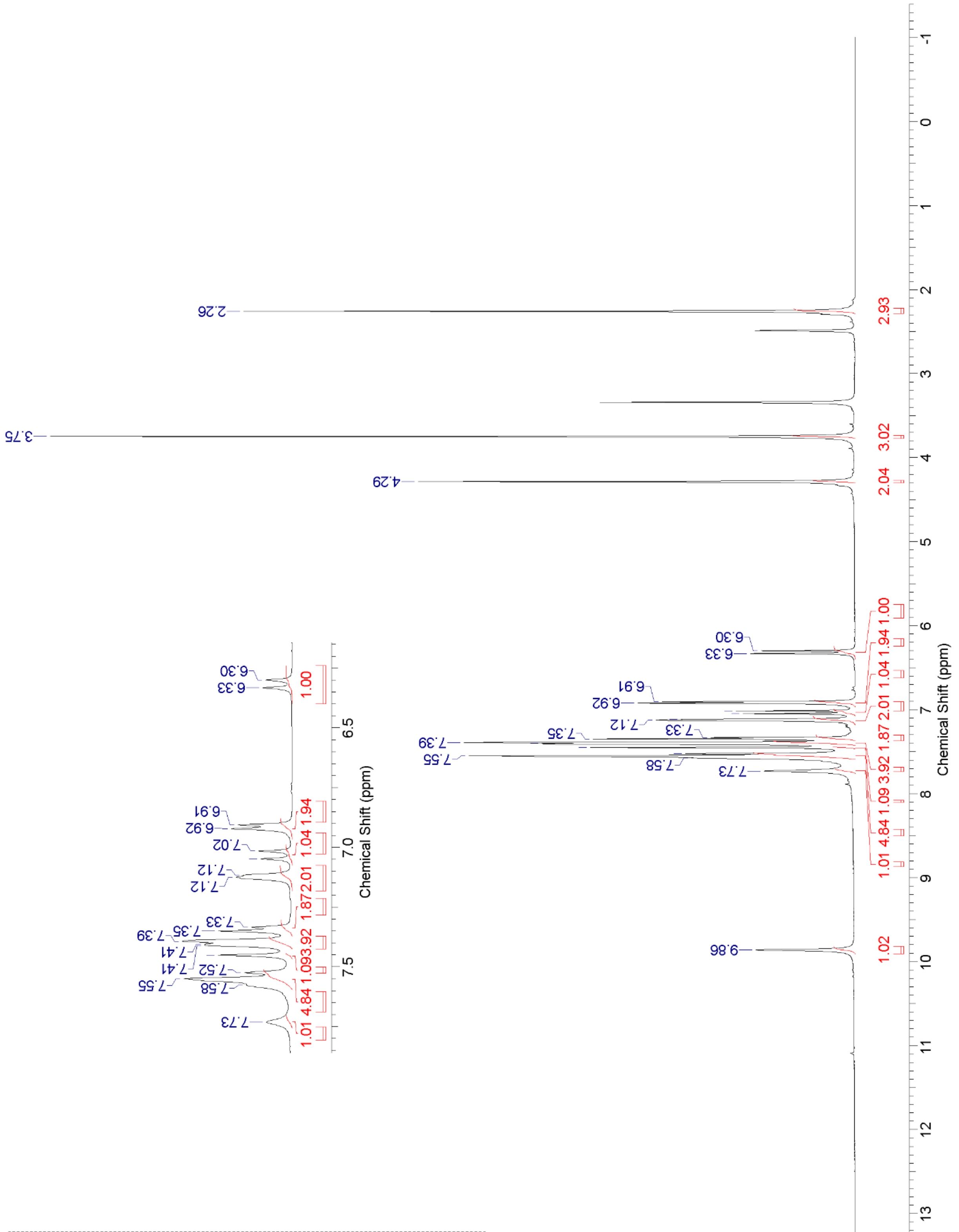

No.	(ppm)	(Hz)
1	2.26	1128.7
2	3.75	1873.4
3	4.29	2141.9
4	6.30	3148.8
5	6.33	3164.9
6	6.91	3451.6
7	6.92	3459.9
8	7.02	3506.3
9	7.05	3522.9
10	7.12	3557.1
11	7.12	3561.0
12	7.33	3665.5
13	7.35	3673.8
14	7.39	3695.3
15	7.41	3701.6
16	7.41	3703.6
17	7.45	3724.1
18	7.52	3760.7
19	7.55	3773.9
20	7.58	3786.6
21	7.73	3864.2
22	9.86	4928.7

Spectrum 47. ^{1}H NMR of compd **31** (500 MHz, DMSO-d_6).

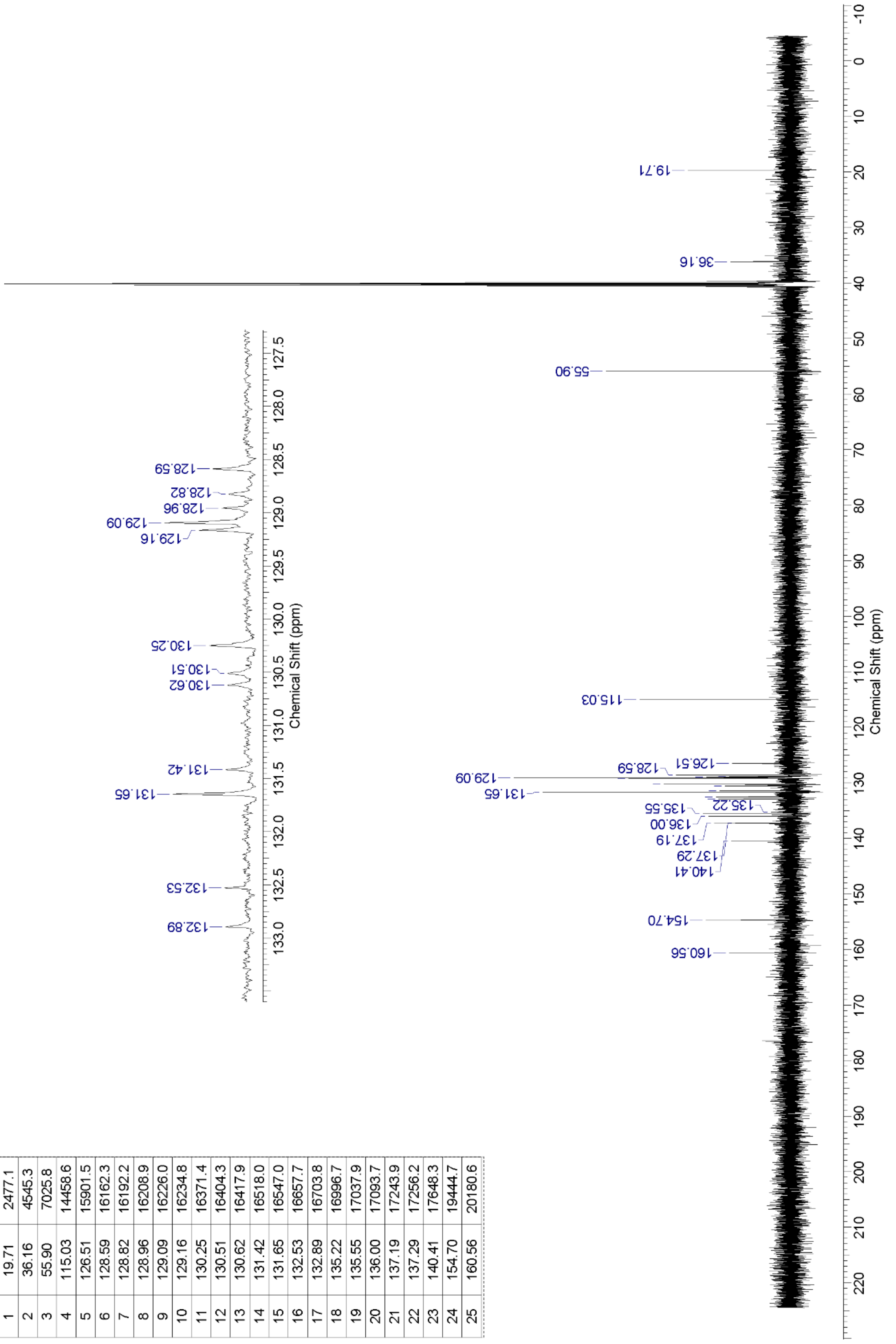

No.	(ppm)	(Hz)
1	19.71	2477.1
2	36.16	4545.3
3	55.90	7025.8
4	115.03	14458.6
5	126.51	15901.5
6	128.59	16162.3
7	128.82	16192.2
8	128.96	16208.9
9	129.09	16226.0
10	129.16	16234.8
11	130.25	16371.4
12	130.51	16404.3
13	130.62	16417.9
14	131.42	16518.0
15	131.65	16547.0
16	132.53	16657.7
17	132.89	16703.8
18	135.22	16996.7
19	135.55	17037.9
20	136.00	17093.7
21	137.19	17243.9
22	137.29	17256.2
23	140.41	17648.3
24	154.70	19444.7
25	160.56	20180.6

Spectrum 48. ^{13}C NMR of compd **31** (125 MHz, DMSO-d_6).

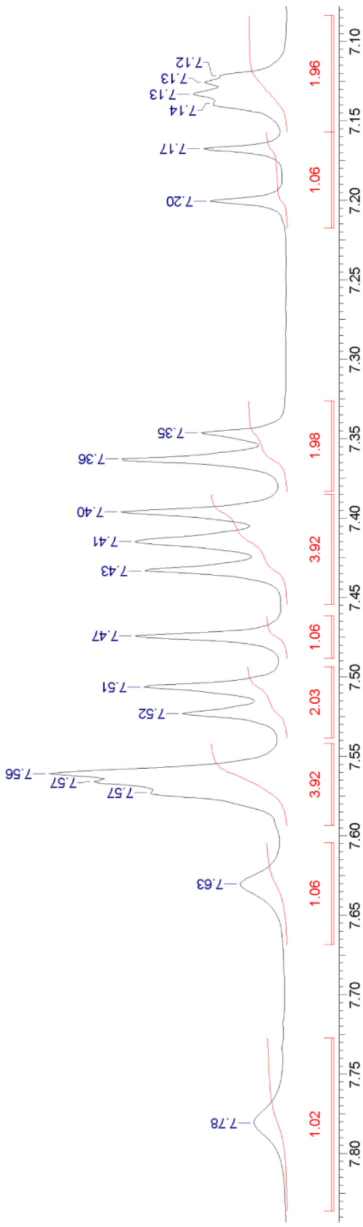

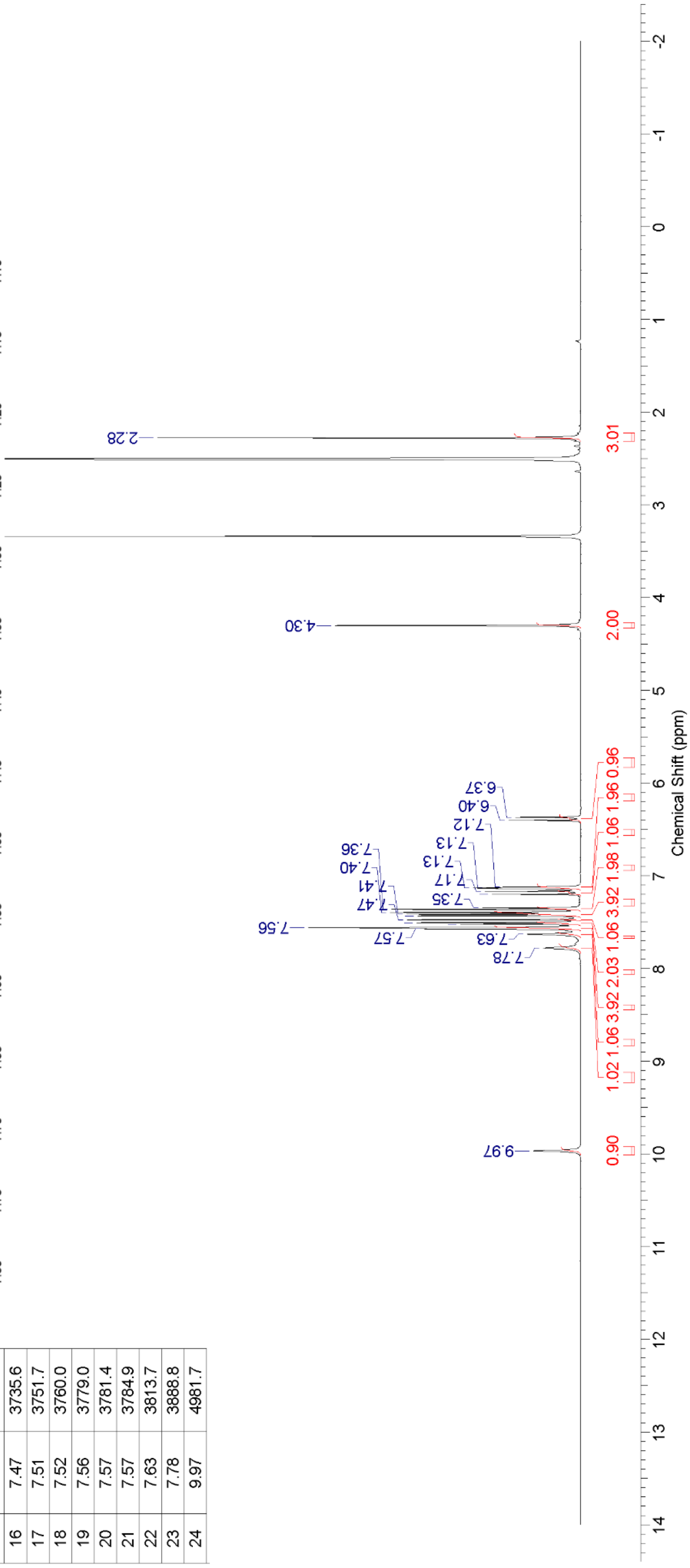

No.	(ppm)	(Hz)
1	2.28	1137.8
2	4.30	2149.7
3	6.37	3183.5
4	6.40	3200.1
5	7.12	3559.3
6	7.13	3561.3
7	7.13	3565.2
8	7.14	3568.1
9	7.17	3582.3
10	7.20	3598.9
11	7.35	3671.6
12	7.36	3679.9
13	7.40	3696.5
14	7.41	3705.8
15	7.43	3715.1
16	7.47	3735.6
17	7.51	3751.7
18	7.52	3760.0
19	7.56	3779.0
20	7.57	3781.4
21	7.57	3784.9
22	7.63	3813.7
23	7.78	3888.8
24	9.97	4981.7

Spectrum 49. ^{1}H NMR of compd **32** (500 MHz, DMSO-d_6).

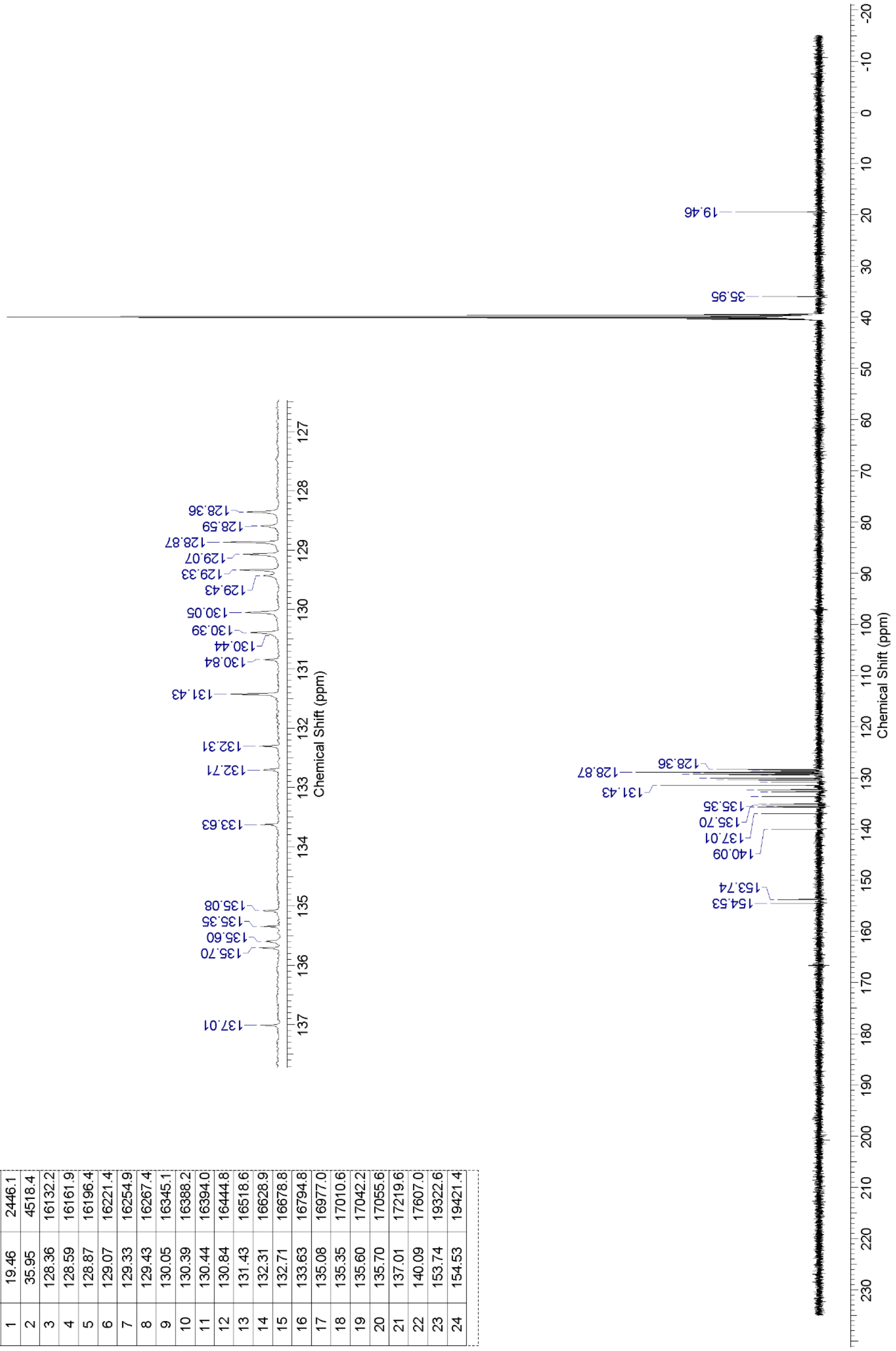

No.	(ppm)	(Hz)
1	19.46	2446.1
2	35.95	4518.4
3	128.36	16132.2
4	128.59	16161.9
5	128.87	16196.4
6	129.07	16221.4
7	129.33	16254.9
8	129.43	16267.4
9	130.05	16345.1
10	130.39	16388.2
11	130.44	16394.0
12	130.84	16444.8
13	131.43	16518.6
14	132.31	16628.9
15	132.71	16678.8
16	133.63	16794.8
17	135.08	16977.0
18	135.35	17010.6
19	135.60	17042.2
20	135.70	17055.6
21	137.01	17219.6
22	140.09	17607.0
23	153.74	19322.6
24	154.53	19421.4

Spectrum 50. ^{13}C NMR of compd **32** (125 MHz, DMSO-d_6).

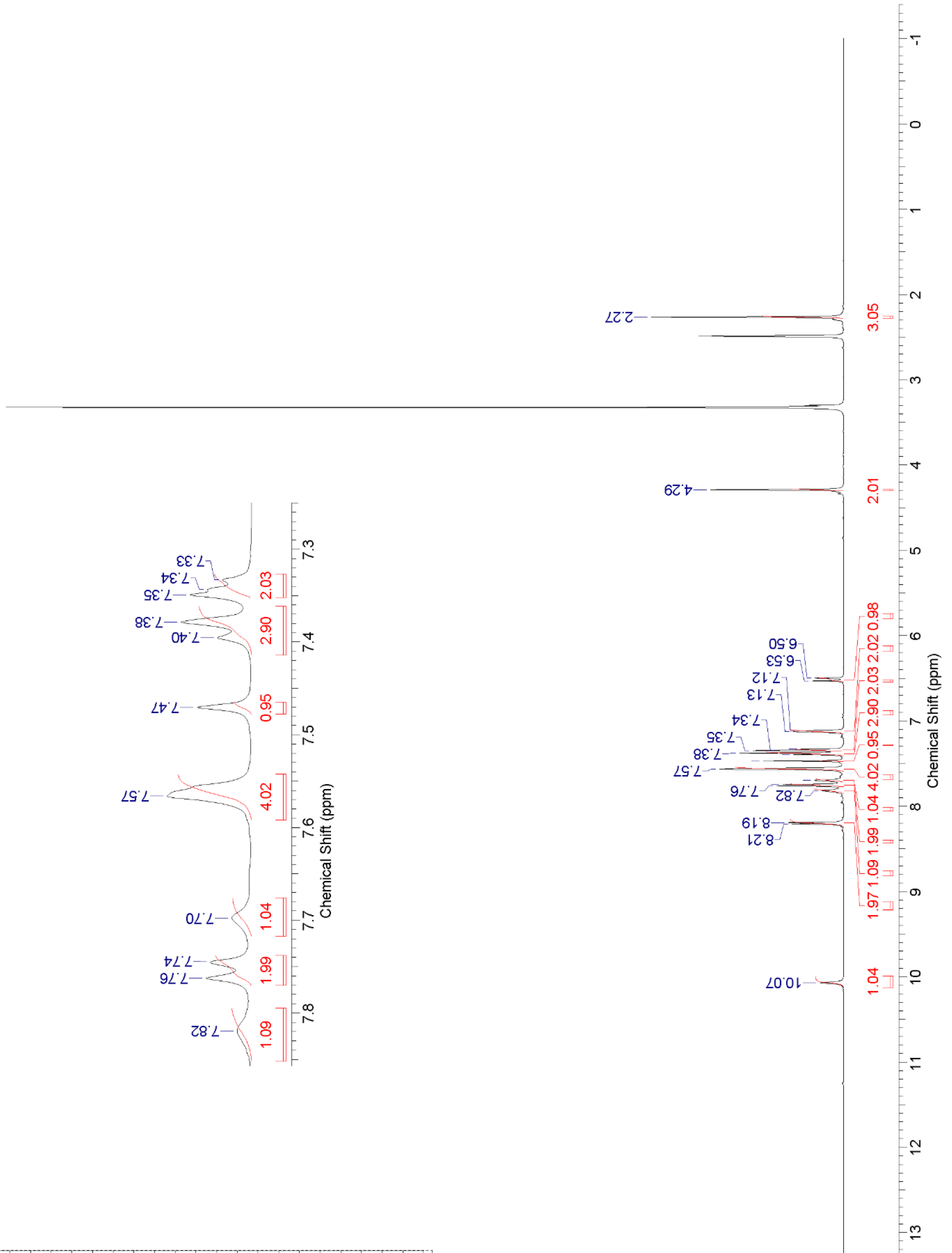

No.	(ppm)	(Hz)
1	2.27	1132.1
2	4.29	2143.9
3	6.50	3248.0
4	6.53	3264.6
5	7.12	3558.0
6	7.13	3561.5
7	7.33	3665.0
8	7.34	3670.3
9	7.35	3673.3
10	7.38	3687.9
11	7.40	3696.2
12	7.47	3733.8
13	7.57	3781.7
14	7.70	3847.6
15	7.74	3871.0
16	7.76	3879.8
17	7.82	3908.2
18	8.19	4094.7
19	8.21	4103.0
20	10.07	5033.2

Spectrum 51. ^{1}H NMR of compd **33** (500 MHz, DMSO-d_6).

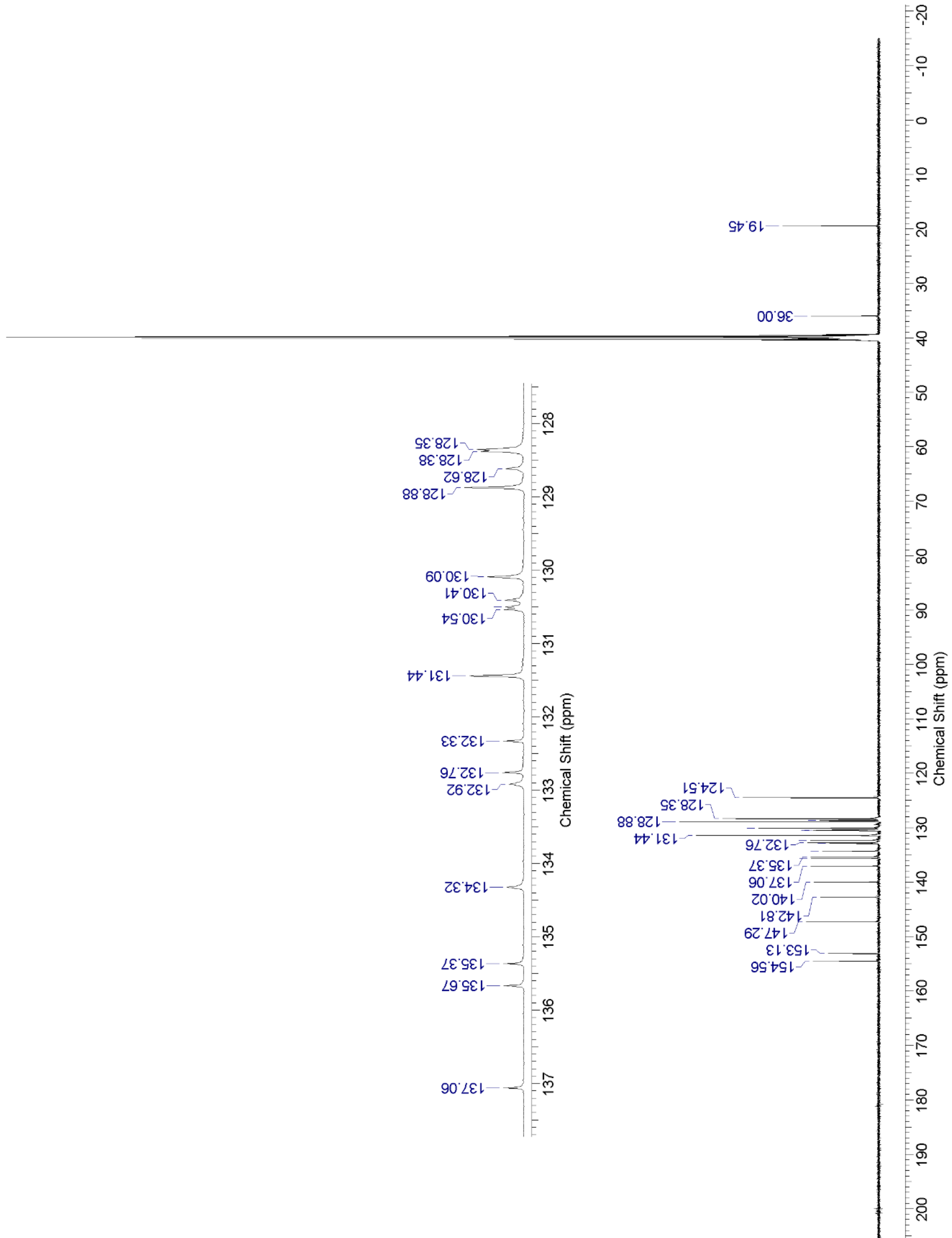

No.	(ppm)	(Hz)
1	19.45	2445.1
2	36.00	4524.1
3	124.51	15648.9
4	128.35	16131.2
5	128.38	16135.1
6	128.62	16164.8
7	128.88	16197.4
8	130.09	16349.9
9	130.41	16390.1
10	130.50	16401.6
11	130.54	16406.4
12	131.44	16519.6
13	132.33	16631.8
14	132.76	16685.5
15	132.92	16705.6
16	134.32	16882.1
17	135.37	17013.4
18	135.67	17050.8
19	137.06	17226.3
20	140.02	17597.4
21	142.81	17948.4
22	147.29	18512.3
23	153.13	19245.9
24	154.56	19425.2

Spectrum 52. ^{13}C NMR of compd **33** (125 MHz, DMSO-d_6).

S.2. Experimental details of crystallographic analysis

	**24**	**31**
Crystal data		
Chemical formula	$C_{30}H_{26}Cl_2N_4O_3S_2 \cdot C_3H_6O$	$C_{31}H_{29}Cl_2N_4O_3S_2^{+} \cdot C_7H_7O_3S^{-}$
M_r	683.64	811.79
Crystal system, space group	Monoclinic, $P2_1/c$	Triclinic, $P\bar{1}$
Temperature (K)	120	120
a, *b*, *c* (Å)	17.587(4), 8.9968(12), 21.780(5)	10.7215(7), 11.5599(7), 17.5610(12)
α, β, γ (°)	90, 105.627 (16), 90	97.061 (5), 104.959 (5), 110.224 (5)
V ($Å^3$)	3318.7 (11)	1918.3 (2)
Z	4	2
Radiation type	Mo *K*α	Mo *K*α
◎ (mm^{-1})	0.37	0.38
Crystal size (mm)	0.34 × 0.08 × 0.05	0.24 × 0.14 × 0.02
Data collection		
Diffractometer	STOE *IPDS* 2T	STOE *IPDS* 2T
No. of measured, independent and observed [$I > 2\sigma(I)$] reflections	19834, 8881, 5409	7519, 7519, 4999
R_{int}	0.037	Merged
$(\sin \theta/\lambda)_{max}$ ($Å^{-1}$)	0.692	0.617
Refinement		
$R[F^2 > 2\sigma(F^2)]$, $wR(F^2)$, S	0.081, 0.209, 1.12	0.179, 0.471, 1.12
No. of reflections	8881	7519
No. of parameters	457	491
H-atom treatment	H-atom parameters constrained	H-atom parameters constrained
	$w = 1/[\sigma^2(F_o^2) + (0.0514P)^2 + 4.7394P]$ where $P = (F_o^2 + 2F_c^2)/3$	$w = 1/[\sigma^2(F_o^2) + (0.1041P)^2 + 38.4383P]$ where $P = (F_o^2 + 2F_c^2)/3$
Δ〉$_{max}$, Δ〉$_{min}$ (e $Å^{-3}$)	0.30, -0.34	0.91, -0.97

S.3. Hydrogen-bond geometry (Å, º) for 24 and 31

**24**				
D—H···*A*	*D*—H	H···*A*	*D*···*A*	*D*—H···*A*
O3—H3···O4	0.84	1.86	2.701 (4)	176
N3—H3*A*···O1	0.88	2.17	2.813 (4)	129
N2—H2*A*···N1^{i}	0.88	2.15	2.997 (4)	163
Symmetry code: (i) -x, -y+1, -z+1.				
**21**				
D—H···A	D—H	H···A	D···A	D—H···A
N1—H1···O6^{i}	0.88	1.85	2.721 (17)	171
N2—H2A···N4	0.89	2.20	2.654 (19)	111
N2—H2A···O4A	0.89	2.42	2.89 (5)	113
N2—H2A···O5	0.89	2.41	3.02 (2)	126
N2—H2B···O4^{i}	0.89	2.22	2.68 (2)	111
N2—H2B···O4A^{i}	0.89	2.39	2.98 (5)	124
N3—H3···O2	0.88	1.97	2.659 (17)	134
C4—H4···O5^{ii}	0.95	2.52	3.41 (2)	155
C4—H4···O5A^{ii}	0.95	2.37	3.31 (5)	168
C7—H7···O1^{iii}	0.95	2.53	3.253 (18)	133
C31—H31A···O5^{iv}	0.98	2.38	2.94 (2)	115
C31—H31A···O5A^{iv}	0.98	2.33	2.85 (5)	112
C33—H33···O5A	0.95	2.01	2.52 (5)	112
Symmetry codes: (i) -x, -y+1, -z; (ii) x, y-1, z; (iii) -x-1, -y, -z; (iv) -x+1, -y+2, -z+1.				
